# Nutraceutical, Nutritional and Phytotherapeutic Interventions for Neuropsychiatric Disorders Associated with Intellectual Disability: Mechanisms and Clinical Evidence

**DOI:** 10.3390/nu18213543

**Published:** 2026-10-27

**Authors:** Federica Cernigliaro, Alessandra Giliberti, Rosaria Nardello

**Affiliations:** 1 Child and Adolescent Neuropsychiatry Unit, Provincial Health Authority of Palermo, 90129 Palermo, Italy; fede.cernigliaro@gmail.com; 2 Department of Health Promotion, Mother and Child Care, Internal Medicine and Medical Specialities “G. D’Alessandro”, University of Palermo, 90127 Palermo, Italy; rosaria.nardello@unipa.it

**Keywords:** intellectual disability, phytotherapy, saffron, *Bacopa monnieri*, curcumin, autism spectrum disorder, ADHD, neuroinflammation

## Abstract

Intellectual disability (ID) is frequently accompanied by a range of neuropsychiatric and neurological comorbidities, including anxiety, depression, autism spectrum disorder (ASD), attention-deficit/hyperactivity disorder (ADHD), sleep disturbances, and epilepsy. Conventional pharmacological treatments often provide partial relief and may be associated with adverse effects, prompting interest in adjunctive strategies such as phytotherapy. This review examines the current evidence on selected plant-derived compounds—particularly *Crocus sativus* (saffron), *Bacopa monnieri*, *Curcuma longa* (curcumin), and other botanicals—in the management of disorders associated with ID. Mechanistic pathways, clinical evidence, and safety considerations are critically discussed. Although some phytotherapeutic interventions have shown preliminary evidence of potential benefit and may modulate pathways involving neuroinflammation, oxidative stress, and neurotransmitter function, direct clinical evidence in individuals with ID remains limited. Further well-designed randomized controlled trials, particularly in ID-specific populations, are needed to establish efficacy, safety, optimal dosing, and clinical applicability.

## 1. Introduction

### 1.1. Background

Intellectual disability (ID) is characterized by significant limitations in intellectual functioning and adaptive behavior, with onset during the developmental period. Beyond cognitive impairment, individuals with ID frequently present with a high prevalence of comorbid psychiatric and neurological conditions, including autism spectrum disorder (ASD), attention-deficit/hyperactivity disorder (ADHD), mood disorders, and epilepsy. These associated conditions may contribute substantially to functional impairment and clinical complexity rather than cognitive deficits alone [1].

Current management of these comorbidities often relies on conventional pharmacological treatments (e.g., antipsychotics, stimulants, or antidepressants). Although these treatments can be effective for specific symptoms and indications, their use may be associated with adverse effects, including metabolic syndrome, sedation, and movement disorders, which may be particularly detrimental in vulnerable pediatric and neurodevelopmental populations. These limitations have contributed to growing clinical and research interest in adjunctive approaches, including phytotherapeutic, nutraceutical, and nutritional interventions. These interventions—defined as plant-derived compounds, specialized nutritional supplements, and micronutrients—may exert effects through multiple biological pathways. Several compounds have demonstrated antioxidant, anti-inflammatory, or neuromodulatory properties in experimental or clinical studies. These mechanisms may be relevant to biological processes implicated in neurodevelopmental disorders, including oxidative stress, neuroinflammation, mitochondrial dysfunction, and alterations of the gut–brain axis. However, the extent to which modulation of these pathways translates into clinically meaningful benefits remains uncertain, particularly in individuals with ID. Therefore, these approaches should currently be regarded primarily as potential adjunctive strategies rather than established alternatives to conventional pharmacological treatment [2].

In this review, we provide a comprehensive analysis of the current evidence for these interventions in the context of neurodevelopmental disorders associated with ID. To clarify the methodological framework underpinning this work, it should be noted that while our primary focus is on disorders associated with ID, the available clinical evidence is largely drawn from research conducted on autism spectrum disorder (ASD) and attention-deficit/hyperactivity disorder (ADHD) cohorts. This approach is justified by the significant clinical and biological overlap between these conditions. Indeed, ID is frequently comorbid with ASD and ADHD, and its symptomatic spectrum often includes core behavioral features—such as irritability, hyperactivity, anxiety, and cognitive dysregulation—that define these disorders. Nevertheless, evidence obtained from ASD and ADHD populations cannot be directly generalized to individuals with ID. Differences in cognitive functioning, adaptive abilities, underlying etiologies, comorbidity profiles, and treatment response may substantially affect clinical outcomes. Accordingly, studies conducted in ASD and ADHD populations are considered here as a source of indirect evidence that may inform hypotheses regarding potential applications in ID, rather than as direct evidence of efficacy in individuals with ID. Shared biological processes reported across these neurodevelopmental conditions, including oxidative stress, neuroinflammation, mitochondrial dysfunction, and alterations in gut–brain signaling, provide an additional rationale for examining these interventions across diagnostic boundaries. However, the clinical significance of these mechanistic similarities remains incompletely established. Thus, extrapolation of findings from ASD and ADHD cohorts to individuals with ID should be approached cautiously and requires validation in specifically characterized ID populations [3,4,5,6]. The primary aim of this review is to critically examine the clinical evidence and biological mechanisms of selected phytotherapeutic agents, nutraceuticals, and micronutrients in managing neuropsychiatric comorbidities in ID, while providing a critical appraisal of their safety, potential drug interactions, and practical clinical applicability.

### 1.2. Methodology

To ensure the transparency, reproducibility, and clinical relevance of this work, the present review was conducted following a structured protocol inspired by international guidelines for narrative syntheses. A comprehensive literature search was performed across major biomedical databases, including PubMed/MEDLINE, Scopus, Embase, and Web of Science, covering contributions published up to August 2026. The search strategy employed a targeted combination of Medical Subject Headings (MeSH) and free-text terms, specifically designed to capture the breadth of our research scope. These search queries systematically combined terms related to our target population—specifically intellectual disability and associated neurodevelopmental disorders, such as autism spectrum disorder, ADHD, and related neuropsychiatric comorbidities—with terms encompassing the phytotherapeutic, nutraceutical, and nutritional interventions under investigation. Regarding our selection criteria, we adopted rigorous standards to prioritize clinical evidence. We included randomized controlled trials, systematic reviews, meta-analyses, and open-label clinical studies that assessed the efficacy and safety of the selected compounds within pediatric and adolescent populations. In addition to clinical data, we integrated preclinical studies—involving both animal models and mechanistic assays—which we deemed essential for establishing the biological rationale and identifying the molecular targets through which these compounds may exert their neurobiological effects. Conversely, we excluded studies focused exclusively on adult neurodegenerative conditions, unless they provided direct, relevant evidence regarding shared pathogenic pathways, such as neuroinflammation or oxidative stress. Furthermore, opinion pieces lacking robust clinical data were excluded from our analysis. The data extraction process was managed by two independent reviewers (F.C. and A.G.) who screened titles, abstracts, and full texts for eligibility, with the final arbitration of any methodological or interpretative discrepancies provided by a third senior author (R.N.). The extracted data included study design, demographic and clinical characteristics of the participants, specific intervention protocols—such as dosages, formulations, and treatment duration—primary clinical endpoints, and reported adverse events. Finally, we performed a critical qualitative synthesis of the collected evidence. Despite the narrative nature of this review, we maintained a rigorous hierarchy, consistently prioritizing evidence from controlled clinical trials over preclinical observations. We placed particular emphasis on highlighting the intrinsic limitations of the current literature, such as methodological heterogeneity, significant variability in supplementation protocols, and the lack of standardization in diagnostic criteria across different studies. These factors remain the primary challenges for the widespread clinical implementation of these therapeutic approaches and have been addressed throughout our critical appraisal.

## 2. Biological Rationale for Integrative Nutritional and Phytotherapeutic Approaches in ID

Several biological processes implicated in ID-associated disorders include oxidative stress, neuroinflammation, neurotransmitter dysregulation, mitochondrial dysfunction, epigenetic dysregulation, and gut–brain axis alterations [7,8,9,10]. These pathways provide a mechanistic rationale for investigating phytotherapeutic, nutraceutical, and nutritional interventions as potential adjunctive strategies. However, their contribution varies across disorders and individuals, and evidence that targeting these pathways translates into clinically meaningful benefits in ID populations remains limited.

Oxidative stress has been repeatedly reported in autism spectrum disorder (ASD) and in some other neurodevelopmental conditions associated with ID. Studies have described increased reactive oxygen species (ROS) production, lipid and protein oxidation, alterations in glutathione redox balance, and changes in endogenous antioxidant systems, including superoxide dismutase and catalase activity [11,12,13]. Excessive oxidative stress may impair neuronal maturation, synaptic plasticity, and mitochondrial bioenergetics while simultaneously increasing vulnerability to neuroinflammation and excitotoxicity. Importantly, oxidative imbalance appears particularly relevant during critical periods of neurodevelopment, potentially contributing to altered neuronal connectivity, behavioral dysregulation, and cognitive impairment. These findings provide a biological rationale for antioxidant-based interventions such as N-acetylcysteine, coenzyme Q10, curcumin, resveratrol, omega-3 fatty acids, and vitamins with antioxidant properties.

Neuroinflammation has also emerged as an increasingly investigated pathophysiological component in neurodevelopmental disorders associated with ID. Increasing evidence suggests that chronic low-grade activation of microglia and astrocytes contributes to abnormal synaptic pruning, altered neuronal connectivity, and behavioral dysfunction. Elevated concentrations of pro-inflammatory cytokines, including interleukin-6 (IL-6), tumor necrosis factor-alpha (TNF-α), and interleukin-1β (IL-1β), have been identified in both peripheral blood and central nervous system tissues of individuals with ASD [14,15,16,17,18]. Neuroinflammatory processes may additionally interact with oxidative stress, mitochondrial dysfunction, and gut dysbiosis, potentially contributing to interconnected disturbances in neuronal and neurodevelopmental homeostasis. Experimental studies further demonstrated that neuroinflammation may disrupt synaptic plasticity, neurotransmitter signaling, and blood–brain barrier integrity, potentially contributing to irritability, repetitive behaviors, anxiety, and cognitive dysfunction. Consequently, several phytotherapeutics, nutraceuticals, and dietary interventions—including curcumin, omega-3 fatty acids, saffron, resveratrol, probiotics, and N-Acetylcysteine (NAC)—have attracted interest because of their anti-inflammatory and immunomodulatory properties.

Alterations in neurotransmitter systems represent another major biological hallmark of ASD and related neurodevelopmental disorders. Abnormalities involving serotonergic, dopaminergic, glutamatergic, and GABAergic signaling have been extensively described and may contribute to impaired social communication, hyperactivity, repetitive behaviors, emotional dysregulation, and cognitive deficits. Dysregulation of the excitatory/inhibitory balance, particularly involving altered glutamatergic and GABAergic signaling, has been proposed as a key mechanism underlying cortical hyperexcitability and behavioral symptoms in ASD. Altered serotonergic and dopaminergic neurotransmission may additionally contribute to anxiety, reward-processing abnormalities, impulsivity, and mood dysregulation. Several phytotherapeutic and nutraceutical interventions appear capable of modulating these pathways. For example, saffron may influence serotonergic and dopaminergic signaling, magnesium physiologically regulates N-methyl-D-aspartate (NMDA) receptor activity through voltage-dependent channel blockade, NAC modulates glutamatergic neurotransmission through the cystine–glutamate antiporter, while *bacopa* and phosphatidylserine may influence cholinergic and dopaminergic pathways associated with cognition and executive functioning [15,16,17,19,20,21,22,23].

Mitochondrial dysfunction is increasingly recognized in subsets of ASD and genetic syndromes associated with intellectual disability. Abnormalities in oxidative phosphorylation, ATP production, mitochondrial respiration, fatty acid oxidation, and acylcarnitine metabolism have been documented in a proportion of individuals with ASD, Fragile X syndrome, Rett syndrome, and other neurodevelopmental conditions. Mitochondrial dysfunction may impair neuronal energy supply, increase oxidative stress, and disrupt calcium homeostasis, thereby contributing to synaptic dysfunction and neurodevelopmental abnormalities. Clinically, mitochondrial impairment has been associated with fatigue, developmental regression, cognitive impairment, seizures, sleep disturbances, and gastrointestinal dysfunction. These findings provide a biological rationale for investigating mitochondria- and bioenergetics-targeted interventions such as L-carnitine, acetyl-L-carnitine, coenzyme Q10, NAC, selected micronutrients involved in mitochondrial metabolism and redox homeostasis, and resveratrol. Importantly, several studies have raised the hypothesis that metabolically defined subgroups may differ in their responsiveness to mitochondria-targeted interventions; however, predictive biomarkers remain insufficiently validated [24,25,26,27,28].

Emerging evidence also implicates epigenetic mechanisms, including alterations in DNA methylation, histone modifications, and chromatin regulation, in several genetic and neurodevelopmental disorders associated with ID. Such mechanisms can influence the expression of genes involved in neuronal differentiation, synaptic plasticity, neuroimmune signaling, and cellular homeostasis without altering the underlying DNA sequence. Experimental studies indicate that selected dietary and plant-derived bioactive compounds, including curcumin and epigallocatechin-3-gallate (EGCG), may influence epigenetic regulatory pathways such as histone acetylation and DNA methylation-related processes. However, these effects are predominantly supported by preclinical or mechanistic evidence, and whether nutritionally achievable exposures can produce clinically meaningful epigenetic modulation in individuals with neurodevelopmental disorders remains uncertain [29,30,31,32].

The gut–brain axis has also become an area of intense interest in neurodevelopmental research. Alterations in gut microbiota composition, intestinal permeability, short-chain fatty acid production, and immune signaling have been repeatedly reported in ASD and related neurodevelopmental disorders. Dysbiosis may influence neurodevelopment through multiple mechanisms, including modulation of immune activation, tryptophan–serotonin metabolism, vagal signaling, microbial metabolite production, and systemic inflammation. Gastrointestinal symptoms such as constipation and abdominal pain are frequently reported in ASD, while alterations in gut microbial composition have also been described. Increasing evidence suggests that gut microbiota alterations may contribute not only to gastrointestinal dysfunction but also to irritability, anxiety, sleep disturbances, and cognitive impairment. Consequently, probiotics, omega-3 fatty acids, curcumin, and dietary interventions have emerged as promising strategies for modulating gut–brain communication and reducing neuroinflammatory burden. Although the precise causal relationships remain incompletely understood, the gut–brain axis represents an important emerging area of mechanistic and therapeutic investigation in neurodevelopmental disorders associated with intellectual disability [33,34,35,36,37,38].

**Figure 1:**
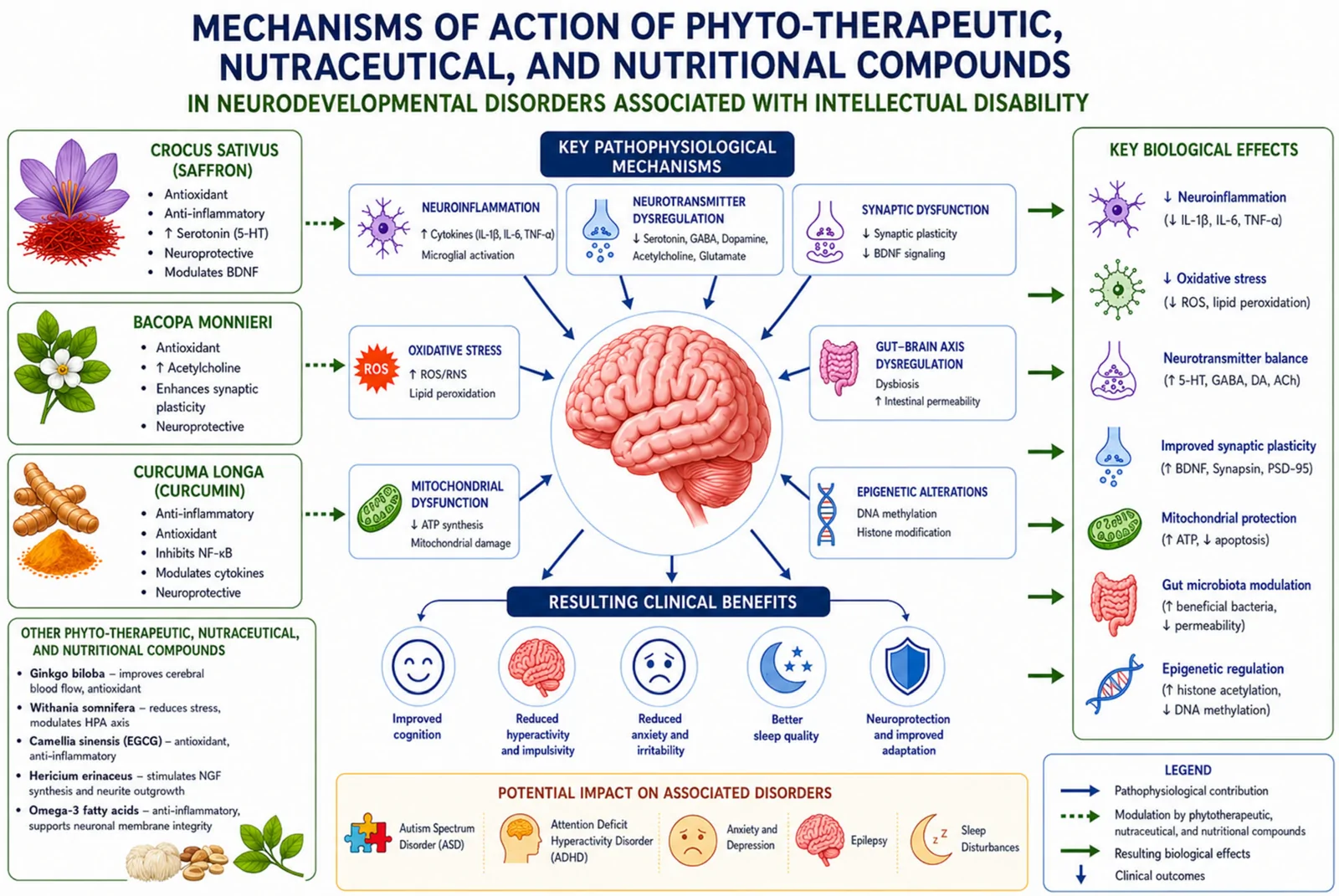
Mechanism of action of phyto-therapeutic, nutraceutical and nutritional compounds in ID-associated disorders.

Phytotherapeutic, nutraceutical, and nutritional compounds frequently act on multiple biological pathways simultaneously, supporting their investigation as potential adjunctive interventions in ID-associated neurodevelopmental disorders. Rather than acting through a single molecular target, many compounds exert pleiotropic effects across redox regulation, inflammatory signaling, mitochondrial function, neurotransmission, neurotrophic pathways, and gut–brain communication. For instance, polyphenols and related bioactive compounds may simultaneously influence oxidative and inflammatory pathways, neuronal signaling, and synaptic plasticity, whereas omega-3 fatty acids can affect membrane composition, inflammatory signaling, and synaptic function. This mechanistic convergence is potentially relevant to the biological heterogeneity of neurodevelopmental disorders; however, pleiotropic activity should not be interpreted as evidence of clinical efficacy. Establishing whether modulation of these pathways translates into meaningful functional improvement will require biomarker-informed clinical studies capable of identifying biologically defined responder subgroups [39,40]. The principal interconnected pathways are summarized in Figure 1.

## 3. Key Phytotherapeutic, Nutraceutical, and Nutritional Agents

The interventions discussed in this section have been categorized based on their primary therapeutic nature, ranging from plant-derived phytotherapeutic agents and specific nutraceuticals to essential micronutrients and dietary supplements. This classification aims to provide a clearer framework for understanding their clinical application and biological mechanisms in the context of neurodevelopmental disorders associated with intellectual disability.

### 3.1. Plant-Derived Phytotherapeutic Agents

#### 3.1.1. *Crocus sativus* (Saffron)

**Figure 2:**
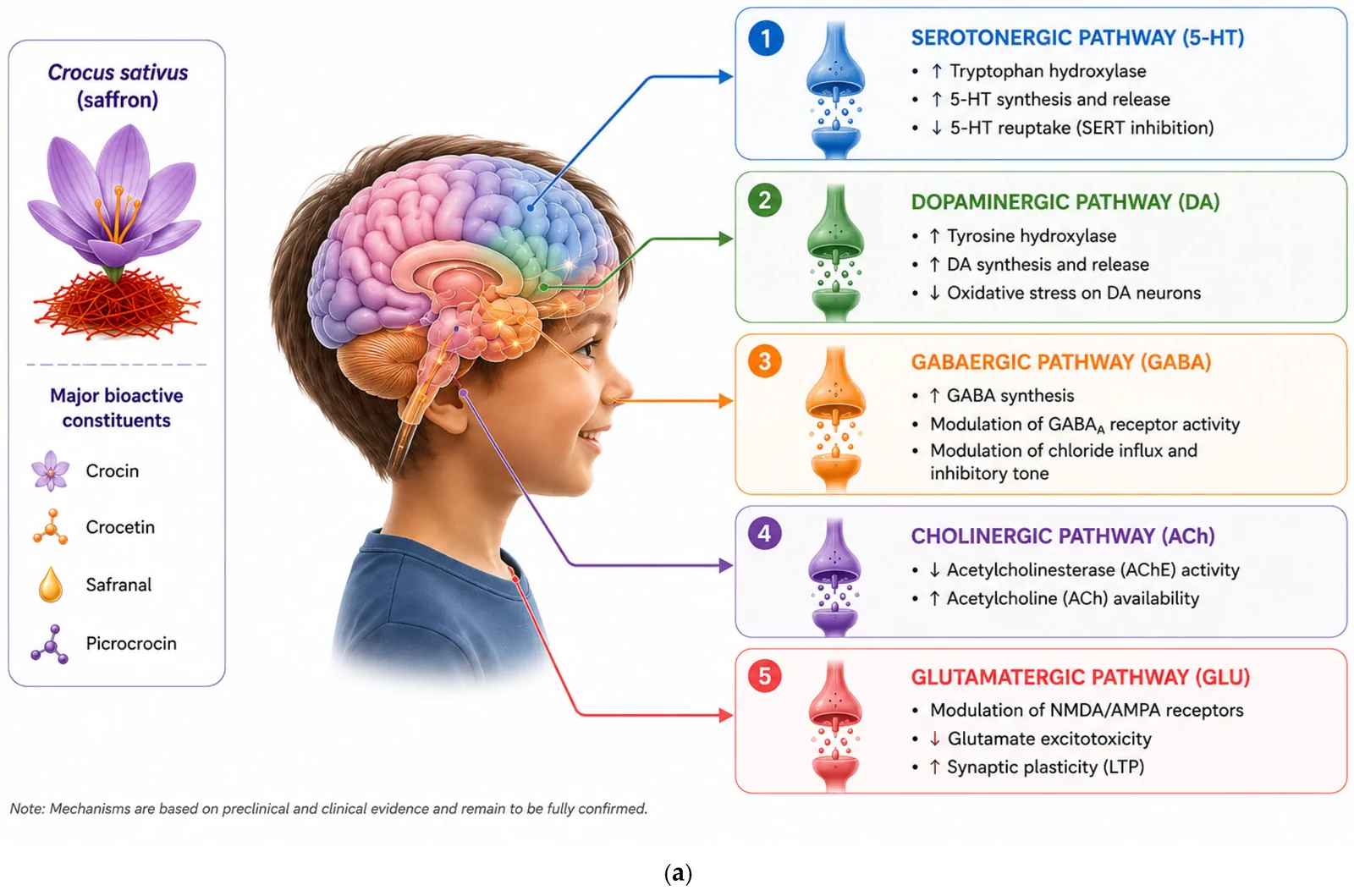

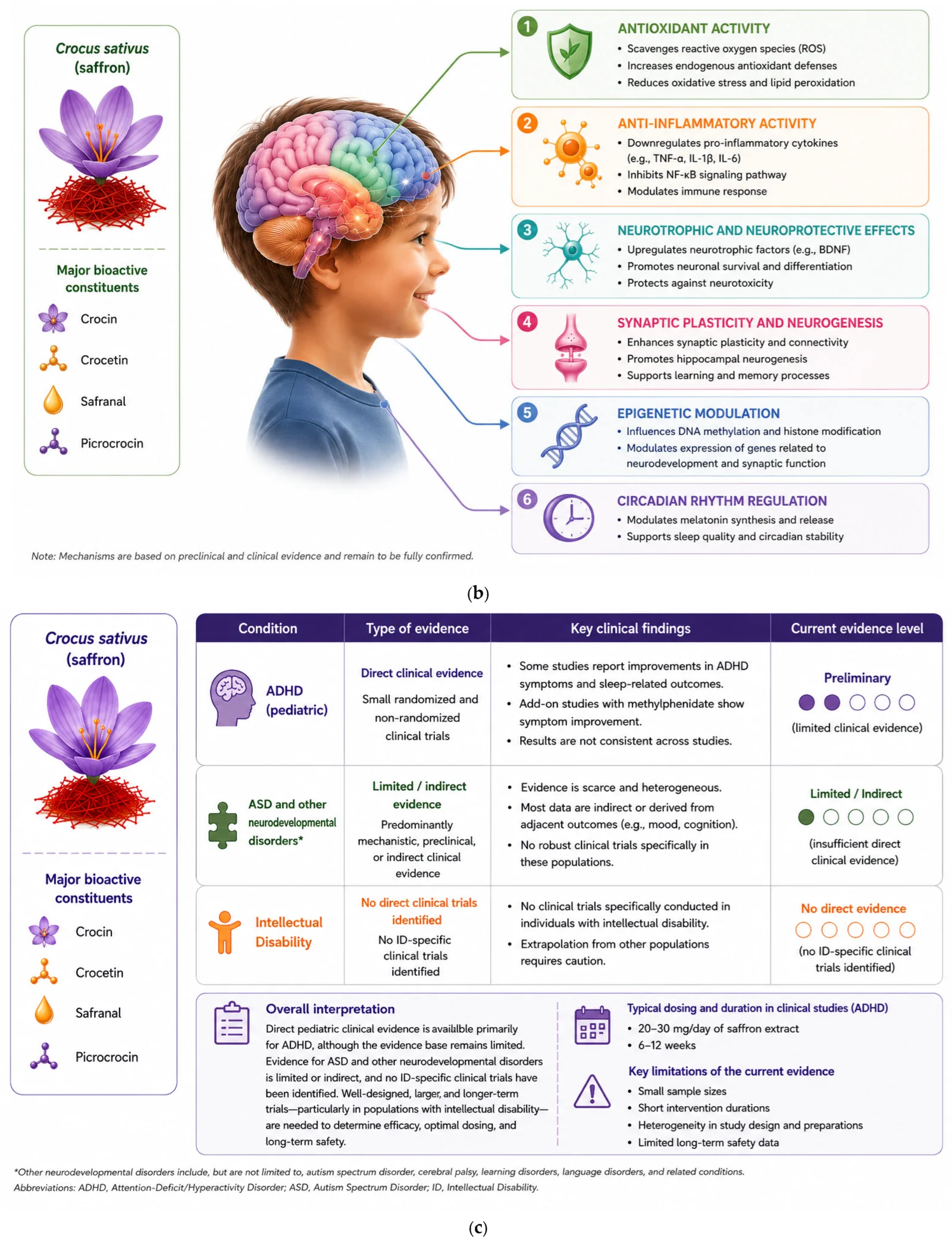
Neurobiological mechanisms and clinical evidence for *Crocus sativus* (saffron) [41,42,43,44,45,46,47,48,49,50,51,52,53,54,55,56,57,58,59,60,61,62,63,64,65,66,67,68,69,70,71,72,73,74,75,76,77,78,79,80,81,82,83,84,85,86,87,88,89,90,91,92,93]. (**a**) Major neurotransmitter pathways potentially modulated by saffron. (**b**) Principal non-neurotransmitter mechanisms, including antioxidant, anti-inflammatory, neurotrophic, epigenetic, synaptic-plasticity, and circadian pathways. (**c**) Summary of current clinical evidence in neurodevelopmental conditions.

*Crocus sativus* L. (saffron) and its major bioactive constituents, including crocin, crocetin, and safranal, have been investigated for potential neuropsychological effects. Proposed mechanisms include modulation of serotonergic and dopaminergic neurotransmission, antioxidant and anti-inflammatory activity, and effects on neuroplasticity. These neurobiological mechanisms and the available clinical evidence are summarized in Figure 2a–c [41,42,43,44,45,46,47,48,49,50,51,52,53,54,55,56,57,58,59,60,61,62,63,64,65,66,67,68,69,70,71,72,73,74,75,76,77,78,79,80,81,82,83,84,85,86,87,88,89,90,91,92,93]. Specifically, Figure 2a illustrates the major neurotransmitter pathways potentially modulated by saffron, while Figure 2b highlights the principal non-neurotransmitter mechanisms, including antioxidant, anti-inflammatory, neurotrophic, epigenetic, synaptic-plasticity, and circadian pathways. Figure 2c provides an overview of the current clinical evidence regarding the potential therapeutic applications of *Crocus sativus* in neurodevelopmental conditions. However, much of the mechanistic evidence derives from experimental models, and its direct relationship to clinical efficacy remains incompletely established. Clinical interest in saffron for attention-deficit/hyperactivity disorder (ADHD) has increased in recent years, although the evidence base remains limited. (Table 1 and Table 2). An early narrative review by Ross (2020) [42] discussed preliminary evidence regarding saffron in pediatric ADHD, including findings suggesting potentially similar effects to stimulant medication in selected outcomes. However, the review emphasized the limited evidence base and the need for larger controlled trials. Subsequent studies have investigated saffron either as monotherapy or in combination with methylphenidate, with some reporting improvements in ADHD symptoms and sleep-related outcomes. Blasco-Fontecilla et al. (2022) [43], for example, reported improvements in ADHD symptoms and sleep parameters following saffron supplementation, although the open-label design and absence of a placebo control limit causal interpretation. In 2021, Khaksarian et al. [44] conducted a randomized double-blind add-on study evaluating saffron combined with methylphenidate, and reported symptomatic improvement. More recently, Seyedi-Sahebari et al. (2024) [45] synthesized findings from randomized controlled trials in a systematic review and concluded that saffron supplementation at doses of 20–30 mg/day may produce improvements across ADHD rating scales while maintaining generally favorable tolerability and safety profiles. Rigillo et al. (2025) [46] reviewed saffron use in pediatric mental disorders and reported potentially similar effects to methylphenidate in the limited available studies. Taken together, the available evidence supports further investigation of saffron as a potential adjunctive intervention in ADHD; however, the small number of studies, limited sample sizes, methodological heterogeneity, and short treatment durations substantially limit the certainty and generalizability of these findings. Larger long-term clinical trials are still necessary to establish definitive efficacy, optimal dosing, and long-term safety [41,42,43,44,45,46].

In addition to its effects in pediatric ADHD, clinical research on saffron has investigated potential antidepressant and neuropsychological effects across multiple randomized controlled trials and meta-analyses. Evidence concerning the effects of saffron on depressive symptoms is more extensive than that available for ADHD but derives predominantly from adult populations and should therefore be interpreted as indirect evidence in the context of ID-associated neuropsychiatric disorders (Table 3 and Table 4). Akhondzadeh et al. (2004) [47] conducted one of the earliest controlled studies comparing saffron supplementation (30 mg/day for 6 weeks) with the tricyclic antidepressant imipramine in adults with major depressive disorder, reporting short-term reductions in depressive symptoms, while showing generally favorable tolerability. In a subsequent placebo-controlled trial, Akhondzadeh et al. (2005) [48] reported greater reductions in HAM-D scores with saffron than with placebo, providing preliminary evidence of an antidepressant effect. During the same year, Noorbala et al. (2005) [49] reported similar short-term improvement to those observed with fluoxetine, one of the most commonly prescribed selective serotonin reuptake inhibitors (SSRIs). These early trials generally involved small samples, short treatment periods, and geographically restricted populations; moreover, active-comparator studies were not necessarily designed or adequately powered to establish formal therapeutic equivalence or non-inferiority. As evidence accumulated, Hausenblas et al. (2013) [50] performed a meta-analysis of randomized controlled trials and reported moderate-to-large effect sizes for saffron in reducing depression severity, concluding that findings were consistent across studies despite relatively small sample sizes. Lopresti and Drummond (2014) [51] subsequently conducted a systematic review and meta-analysis that further supported saffron’s antidepressant activity, highlighting its potential mechanisms involving serotonergic modulation, antioxidant activity, anti-inflammatory effects, and regulation of neuroendocrine pathways associated with mood disorders. Expanding the therapeutic scope to specific clinical populations, Talaei et al. (2015) [52] investigated saffron supplementation using crocin, a major bioactive constituent of saffron, in major depression and observed significant improvements in depressive symptoms compared with placebo, providing additional preliminary evidence of potential antidepressant effects in psychologically vulnerable populations. Overall, these early studies reported short-term improvements in depressive symptoms; however, their small samples, short treatment durations, and restricted study populations preclude firm conclusions regarding comparative efficacy with conventional antidepressants. Larger multicenter trials with longer follow-up periods remain necessary to confirm long-term efficacy, optimal dosing strategies, and broader applicability across psychiatric populations [47,48,49,50,51,52].

More recent clinical trials and quantitative syntheses have further evaluated saffron in mood-related outcomes (Table 5 and Table 6). In a randomized controlled trial, Lopresti and Drummond [53] investigated a standardized curcumin–saffron combination in adults with major depressive disorder and reported short-term improvements in depressive and anxiety symptoms compared with placebo. However, because saffron was administered in combination with curcumin, the independent contribution of saffron cannot be determined. As the volume of clinical data expanded, rigorous statistical syntheses further consolidated these findings. Tóth [54] et al. conducted a meta-analysis of randomized controlled trials and reported reductions in depressive symptom severity with oral saffron compared with placebo, while no significant differences were observed relative to antidepressant comparators in the included trials. However, these findings should not be interpreted as establishing therapeutic equivalence or non-inferiority. Similarly, Dai et al. [55] conducted a systematic review and meta-analysis assessing clinical trials on mild-to-moderate depressive disorders; their findings indicated improvements relative to placebo and no significant differences relative to antidepressant comparators in the included trials, maintaining a generally good safety and tolerability profile with no significant differences in adverse effect incidence compared to controls, while emphasizing the limited duration, sample sizes, and geographical diversity of the available trials. Finally, to resolve conflicting findings across the literature, Musazadeh et al. [56] published an umbrella meta-analysis aggregating data from multiple prior meta-analyses. Their overview confirmed that adjunctive saffron consumption leads to a reduction in standardized depression severity scores, such as the Beck Depression Inventory (BDI), proposing saffron as a natural intervention for mood modulation in psychiatric and neurodevelopmental research; however, effects were not consistent across all depression measures. In 2026, Mahmoudi et al. [57] published a GRADE-assessed systematic review and meta-analysis of 34 randomized controlled trials involving 1769 adults. Significant reductions were observed in self-reported Beck Depression Inventory (BDI) and Beck Anxiety Inventory (BAI) scores following saffron supplementation. In contrast, significant effects were not observed for other measures, including the Hamilton Depression Rating Scale (HDRS), Hamilton Anxiety Rating Scale (HARS), and Profile of Mood States (POMS), and the overall certainty of evidence was rated as moderate. Collectively, these high-tier syntheses indicate that saffron may provide antidepressant and anxiolytic benefits, although multicenter trials with even longer follow-up periods remain necessary to optimize standardized dosing protocols across diverse clinical populations [53,54,55,56,57]. Interpretation remains constrained by heterogeneity in saffron preparations and standardization, relatively short intervention periods, small samples in several primary studies, geographical concentration of some of the evidence, and limited long-term safety data. Importantly, direct clinical evidence in individuals with intellectual disability remains lacking. Consequently, the available findings should be regarded as indirect and hypothesis-generating with respect to ID-associated neuropsychiatric disorders, and adequately powered trials in well-characterized ID populations are required before clinical recommendations can be made.

Preclinical studies have additionally suggested that constituents of *Crocus sativus*, particularly safranal, may exert anticonvulsant effects through mechanisms involving GABAergic neurotransmission and modulation of inflammatory and oxidative pathways. However, these observations derive predominantly from experimental models, and clinical evidence supporting anticonvulsant efficacy in humans remains insufficient. These findings should therefore be considered mechanistic rather than therapeutic evidence [41,42,43,44,45,46,47,48,49,50,51,52,53,54,55,56,57,58,59,60,61,62,63,64,65,66,67,68,69,70,71,72,73,74,75,76,77,78,79,80,81,82,83,84,85,86,87,88,89,90,91,92,93].

#### 3.1.2. *Bacopa monnieri*

**Figure 3:**
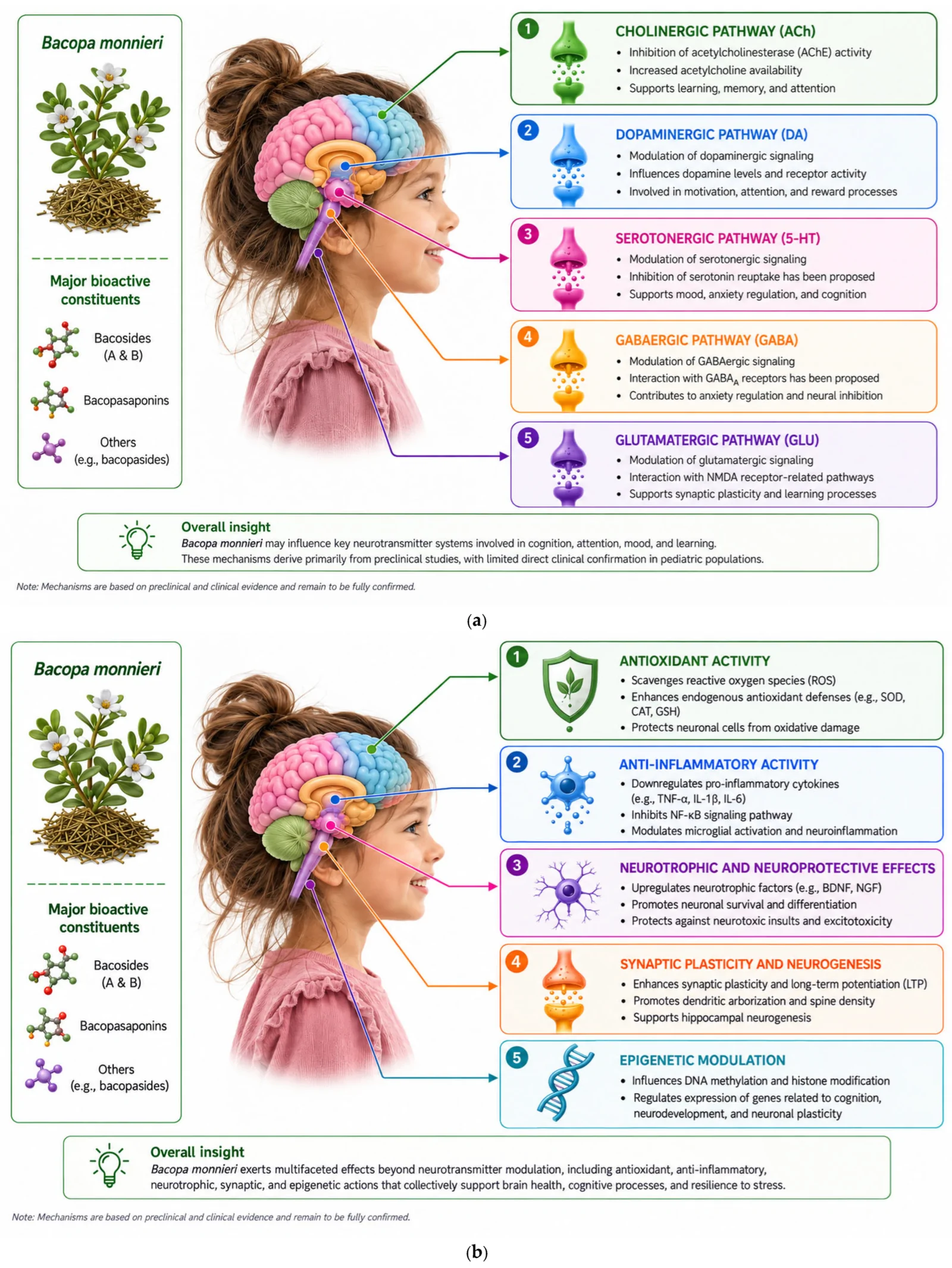

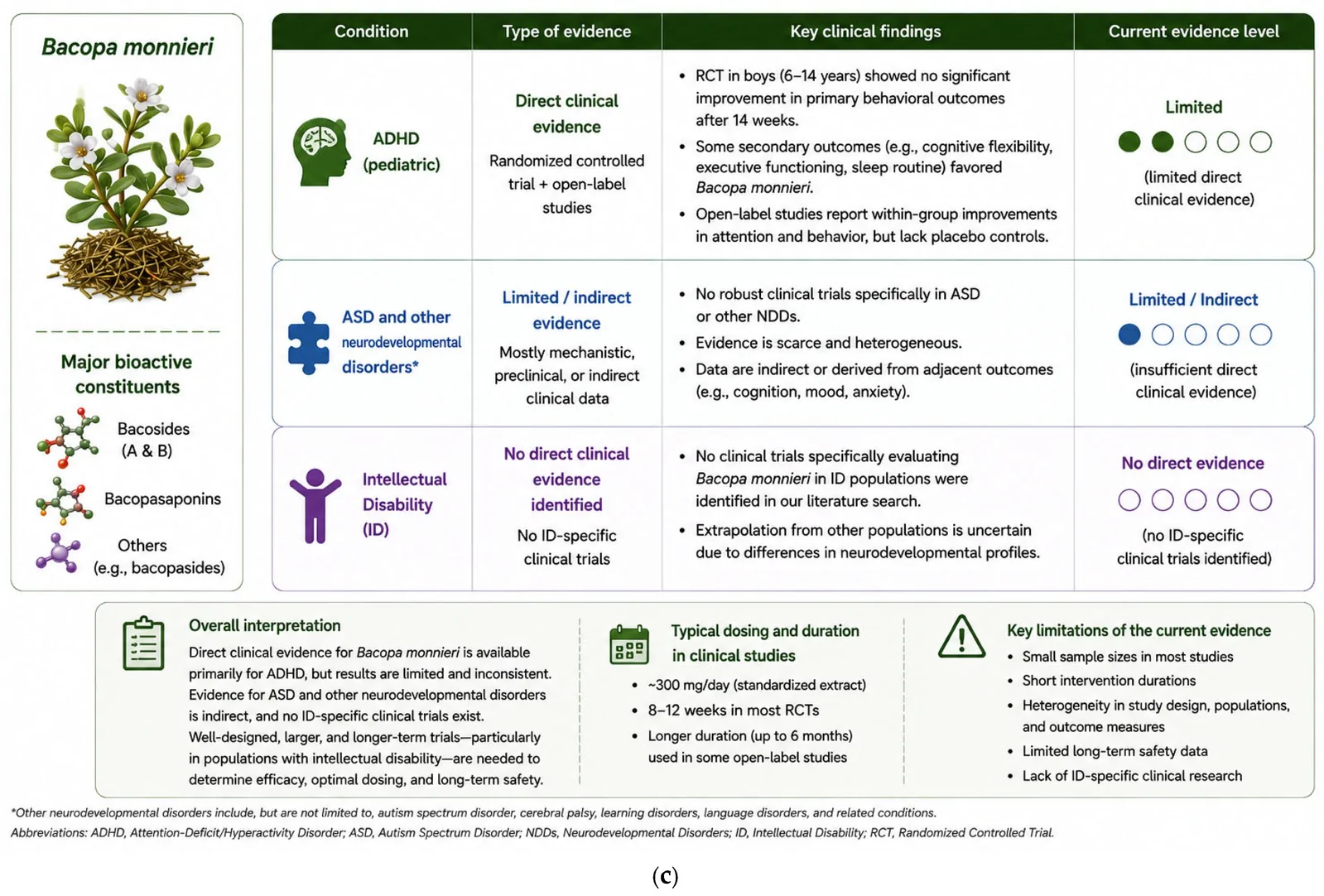
Neurobiological mechanisms and clinical evidence for *Bacopa monnieri* [94,95,96,97,98,99,100,101,102,103,104,105,106,107,108,109,110,111,112,113,114,115,116,117,118,119,120,121,122,123,124,125,126,127,128,129]. (**a**) Major neurotransmitter pathways potentially modulated by *Bacopa monnieri*. (**b**) Principal non-neurotransmitter mechanisms, including antioxidant, anti-inflammatory, neurotrophic, epigenetic, synaptic-plasticity, and potential epigenetic modulation. (**c**) Summary of current clinical evidence in neurodevelopmental conditions.

*Bacopa monnieri* has a long history of use in Ayurvedic medicine, particularly in preparations traditionally intended to support memory and cognitive function. Its principal bioactive constituents include bacosides, which have been investigated for potential effects on cholinergic signaling, synaptic plasticity, oxidative pathways, and neuronal function. These neurobiological mechanisms and the available clinical evidence are summarized in Figure 3a–c [94,95,96,97,98,99,100,101,102,103,104,105,106,107,108,109,110,111,112,113,114,115,116,117,118,119,120,121,122,123,124,125,126,127,128,129]. Specifically, Figure 3a illustrates the major neurotransmitter pathways potentially modulated by *Bacopa monnieri*, while Figure 3b highlights the principal non-neurotransmitter mechanisms, including antioxidant, anti-inflammatory, neurotrophic, synaptic-plasticity, and potential epigenetic pathways. Figure 3c provides an overview of the current clinical evidence regarding the potential therapeutic applications of *Bacopa monnieri* in neurodevelopmental conditions. Clinical research on *B. monnieri* has predominantly focused on cognitive outcomes in healthy adults and older populations rather than individuals with neurodevelopmental disorders. Several controlled trials have investigated standardized extracts, commonly administered at approximately 300 mg/day for 8–12 weeks, and have reported improvements in selected cognitive outcomes, particularly memory and attention. (Table 7 and Table 8) In a double-blind randomized, placebo-controlled study, in 2002, Roodenrys et al. [102] reported improvement with Brahmi *Bacopa monnieri* on a test for the retention of new information. Follow-up tests showed that the rate of learning was unaffected, suggesting that Brahmi decreases the rate of forgetting of newly acquired information. Tasks assessing attention, verbal and visual short-term memory and the retrieval of pre-experimental knowledge were unaffected. Questionnaire measures of everyday memory function and anxiety levels were also unaffected. Raghav et al. [103] similarly reported improvements in selected memory measures among adults with age-associated memory impairment, while Calabrese et al. [104] observed improvements in delayed recall and changes in anxiety and depression scores in healthy older adults. Peth-Nui et al. [105]. reported changes in selected cognitive measures together with reduced acetylcholinesterase activity in healthy older volunteers. Although these findings support potential effects on specific cognitive domains, their relevance to pediatric neurodevelopmental disorders and, particularly, to intellectual disability (ID) remains indirect because of substantial differences in age, baseline cognitive function, and clinical characteristics. In the pre-clinical domain, Uabundit et al. [106] utilized an AF64A-induced cholinergic degeneration model of Alzheimer’s disease to demonstrate that *Bacopa* administration mitigates neuronal loss and oxidative stress within the hippocampus, providing a neuroprotective rationale for its cognitive effects. Such findings may contribute to understanding potential neurobiological mechanisms but cannot establish cognitive or therapeutic efficacy in humans. Importantly, subsequent randomized placebo-controlled evidence has provided a more qualified picture. Kean et al. [107]. conducted a randomized, double-blind, placebo-controlled trial of a standardized *B. monnieri* extract (CDRI 08^®^) in 112 boys aged 6–14 years with elevated levels of inattention and hyperactivity, with 93 participants included in the final analysis. After 14 weeks, no significant between-group differences were observed in the primary behavioral outcomes. Some secondary findings favored *B. monnieri*, including measures of cognitive flexibility, executive functioning, interpersonal problems, sleep routine, and reduced error rates, whereas reaction-time speed favored placebo. Given the absence of improvement in the principal behavioral outcomes and the multiplicity of secondary measures, these findings should be considered exploratory rather than evidence of established efficacy for ADHD symptoms [108]. A meta-analysis by Kongkeaw et al. (2014) [109], which included nine randomized controlled trials, found evidence of improvement in some cognitive outcomes, particularly measures related to attention speed. However, the authors emphasized the need for larger, well-designed clinical trials using standardized preparations before definitive conclusions regarding efficacy could be drawn. Evidence in pediatric populations is substantially more limited and heterogeneous. Dave et al. [110] conducted an open-label pilot study suggesting potential therapeutic benefits, noting improvements in attention, learning capacity, and behavioral regulation in children with attention-deficit/hyperactivity disorder (ADHD); however, these preliminary findings are constrained by small sample sizes and a lack of placebo controls. Similarly, a systematic review by Kean et al. [111] concluded that while *Bacopa* shows some improvement in long-term memory retrieval, substantial methodological heterogeneity persists across the pediatric literature. The more recent secondary literature supports similarly cautious interpretation. A 2025 systematic review examining nutritional supplements for ADHD identified potentially favorable findings for *B. monnieri* across the limited available studies but emphasized that the current evidence is insufficient to replace established stimulant treatment and that further studies are required to determine efficacy, optimal dosing, and safety [112]. Moreover, a 2026 systematic review and network meta-analysis of cognitive effects in healthy adults reported potential benefits of *B. monnieri* for some memory domains, particularly at higher doses, but found no significant differences in sustained attention, selective attention, or processing speed [113]. Because this evidence derives from healthy adult populations, its applicability to children with ADHD or individuals with ID is limited. No clinical trials specifically evaluating *Bacopa monnieri* in populations with intellectual disability were identified in our literature search. Therefore, the available evidence is derived primarily from healthy adult, older adult, and ADHD populations, and its applicability to the heterogeneous neurodevelopmental profiles associated with ID remains uncertain [94,95,96,97,98,99,100,101,102,103,104,105,106,107,108,109,110,111,112,113,114,115,116,117,118,119,120,121,122,123,124,125,126,127,128,129].

#### 3.1.3. *Curcuma longa* (Curcumin)

**Figure 4:**
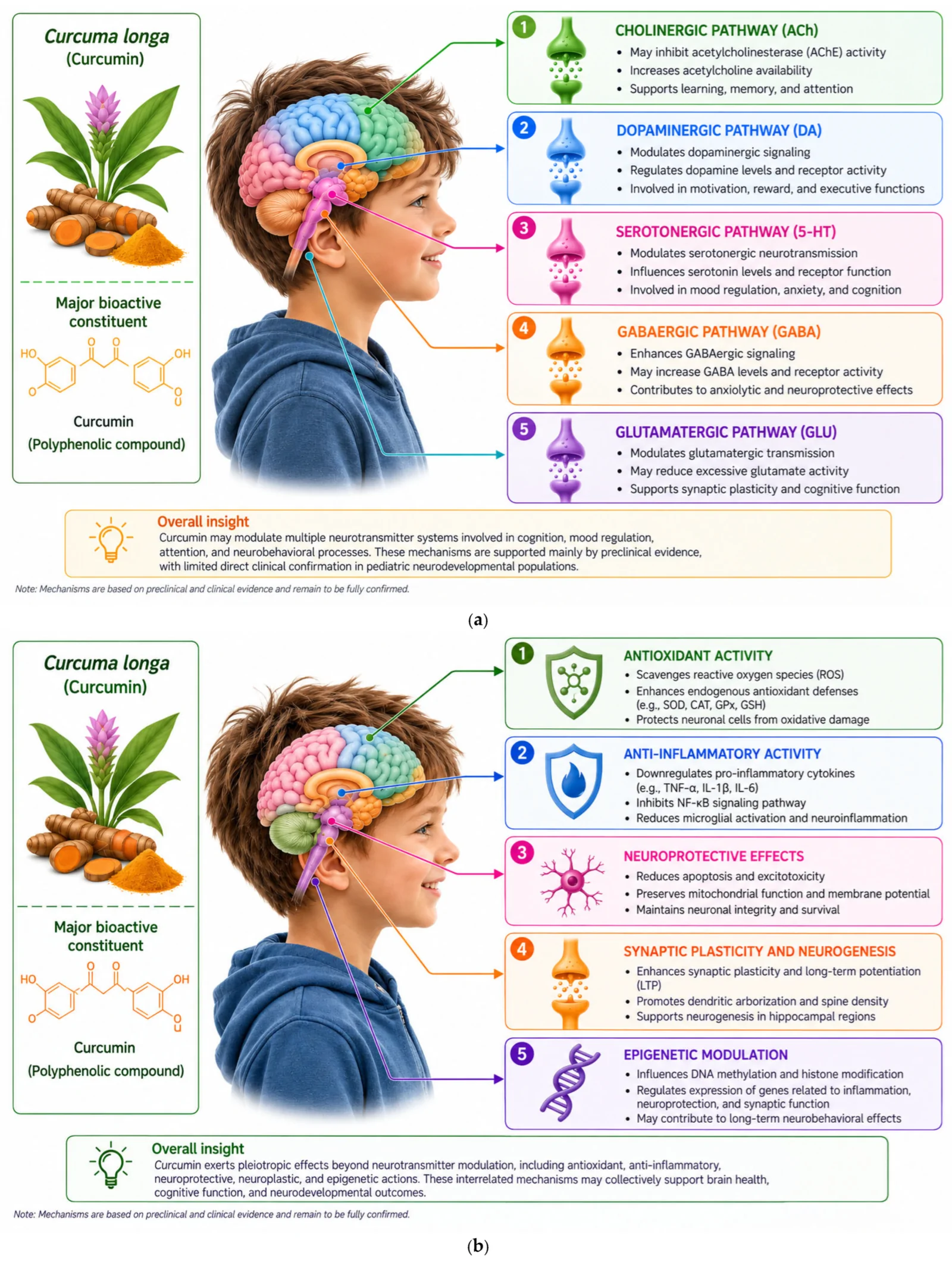

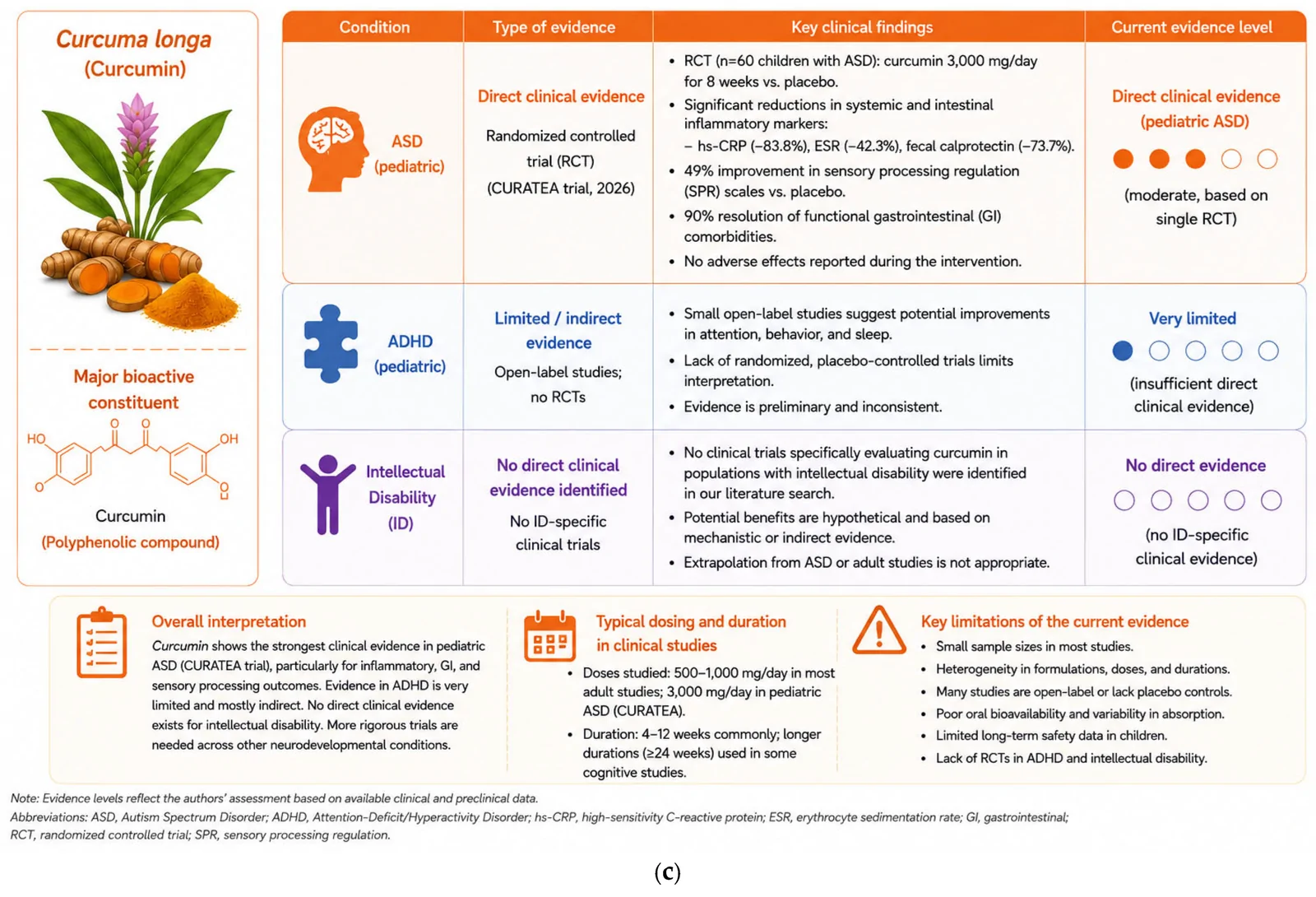
Neurobiological mechanisms and clinical evidence for *Curcuma longa* (*curcumin*) [39,130,131,132,133,134,135,136,137,138,139,140,141,142,143,144,145,146,147,148,149,150,151,152,153,154,155,156,157,158,159,160,161,162]. (**a**) Major neurotransmitter pathways potentially modulated by *Curcuma longa*. (**b**) Principal non-neurotransmitter mechanisms, including antioxidant, anti-inflammatory, neurotrophic, epigenetic, and synaptic plasticity. (**c**) Summary of current clinical evidence in neurodevelopmental conditions.

Curcumin, the principal polyphenolic constituent of *Curcuma longa*, has been extensively investigated for its antioxidant, anti-inflammatory, and neuroprotective properties. These neurobiological mechanisms and the available clinical evidence are summarized in Figure 4a–c [39,130,131,132,133,134,135,136,137,138,139,140,141,142,143,144,145,146,147,148,149,150,151,152,153,154,155,156,157,158,159,160,161]. Specifically, Figure 4a illustrates the major neurotransmitter pathways potentially modulated by *Curcuma longa* (curcumin), while Figure 4b highlights the principal non-neurotransmitter mechanisms, including antioxidant, anti-inflammatory, neurotrophic, epigenetic, and synaptic-plasticity pathways. Figure 4c provides an overview of the current clinical evidence regarding the potential therapeutic applications of *Curcuma longa* (curcumin) in neurodevelopmental conditions. Clinical studies in neurological and psychiatric populations have employed heterogeneous formulations and dosing regimens, while direct evidence in pediatric neurodevelopmental disorders remains comparatively limited (Table 9 and Table 10). Preclinical studies have provided evidence for several neurobiological mechanisms potentially relevant to neurodevelopmental conditions. Bharal et al. [131] reported anticonvulsant effects of curcumin in experimental seizure models, including increased seizure threshold and attenuation of seizure-related activity. These findings have been attributed, at least in part, to antioxidant effects and modulation of excitatory neurotransmission. Similarly, the mechanistic and experimental literature has described anti-inflammatory, antioxidant, anti-apoptotic, and mitochondrial effects that may contribute to neuronal protection, although their clinical relevance to neurodevelopmental disorders remains to be established. Clinical evidence has predominantly been derived from adult psychiatric populations. In a randomized controlled trial, Lopresti et al. [132] evaluated curcumin supplementation (1000 mg/day for 8 weeks) in adults with major depressive disorder and reported improvements in depressive symptoms and selected associated outcomes. Subsequent meta-analytic evidence, including the analysis by Ng et al. [133]., reported reductions in depressive symptom severity associated with curcumin supplementation, although heterogeneity in formulations, dosing regimens, and study populations limits direct comparison across trials. Hewlings and Kalman [134] further summarized the broad antioxidant and anti-inflammatory actions of curcumin, including modulation of NF-κB-related signaling and inflammatory mediators, while emphasizing its relatively poor oral bioavailability as an important translational limitation. Evidence relevant to ASD initially emerged predominantly from experimental models. Bhandari and Kuhad [135] evaluated curcumin in a propionic acid-induced rat model of ASD and reported attenuation of behavioral abnormalities together with reductions in neuroinflammatory and oxidative-stress markers and improvements in mitochondrial-related parameters. These findings provide mechanistic support for investigating curcumin in ASD but cannot, by themselves, establish clinical efficacy. The more recent literature continues to identify oxidative stress, mitochondrial dysfunction, and neuroinflammatory signaling as potential targets of curcumin in experimental ASD models. Importantly, direct pediatric evidence has recently become available from the CURATEA [136] randomized, double-blind, placebo-controlled trial. The study included 60 children with ASD who received curcumin (3000 mg/day) or placebo for 8 weeks. Compared with placebo, curcumin supplementation was associated with significant reductions in systemic and intestinal inflammatory markers, including hs-CRP, ESR, and fecal calprotectin. Improvements were also reported in sensory-processing regulation, together with resolution of functional gastrointestinal symptoms in a substantial proportion of participants. No adverse effects were reported during the intervention. These findings provide direct preliminary clinical evidence linking curcumin supplementation with inflammatory, gastrointestinal, and sensory outcomes in pediatric ASD; however, replication in independent and larger cohorts, evaluation of longer treatment periods, and additional assessment of safety and clinically relevant neurobehavioral outcomes are required. Recent quantitative evidence has also examined the effects of curcumin on cognition. A 2025 systematic review [137] and meta-analysis including nine randomized controlled trials (501 participants) reported an improvement in global cognitive function compared with placebo. However, subgroup analyses indicated that significant effects were primarily observed in studies lasting at least 24 weeks and in participants aged ≥60 years, substantially limiting extrapolation to pediatric neurodevelopmental populations.

Overall, the available evidence supports further investigation of curcumin as a potential adjunctive intervention targeting inflammatory, oxidative, and neurobiological pathways relevant to neurodevelopmental disorders [138,139,140,141,142,143,144,145,146,147,148,149,150,151,152,153,154,155,156,157,158,159,160,161]. The CURATEA trial provides direct clinical evidence in pediatric ASD, but evidence remains insufficient to establish efficacy across the broader spectrum of neurodevelopmental conditions. In particular, no clinical trials specifically evaluating curcumin in populations with intellectual disability as the primary target population were identified in our literature search. Consequently, extrapolation to individuals with ID should remain cautious until ID-specific clinical studies become available [130,131,132,133,134,135,136,137,138,139,140,141,142,143,144,145,146,147,148,149,150,151,152,153,154,155,156,157,158,159,160,161,162].

### 3.2. Nutraceuticals and Bioactive Compounds

#### 3.2.1. N-Acetylcysteine

**Figure 5:**
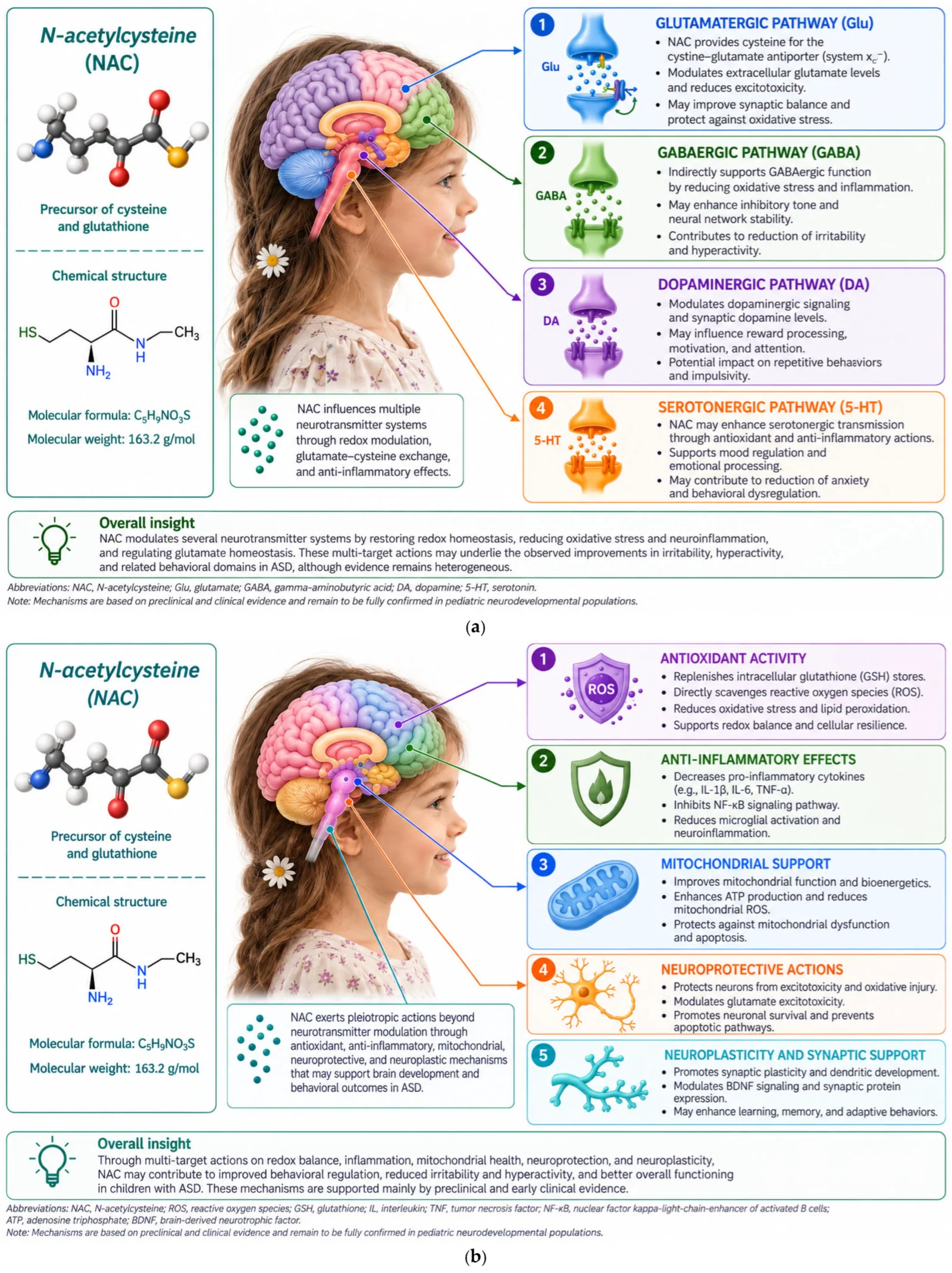

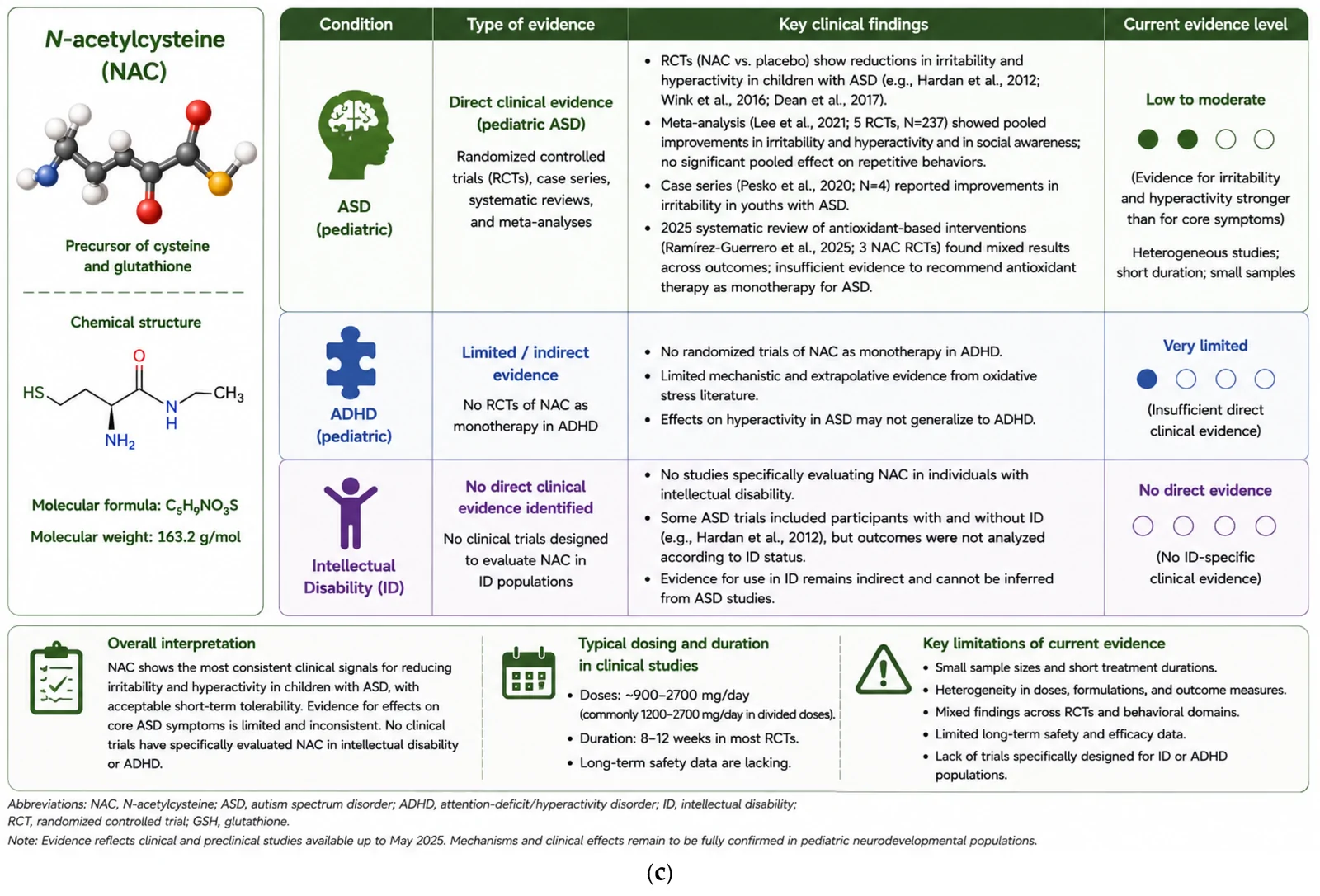
Neurobiological mechanisms and clinical evidence for N-acetylcysteine [163,164,165,166,167,168,169,170,171,172,173,174,175,176,177,178,179,180,181,182,183,184,185,186,187,188]. (**a**) Major neurotransmitter pathways potentially modulated by N-acetylcysteine. (**b**) Principal non-neurotransmitter mechanisms, including glutathione/redox regulation, anti-inflammatory and immune modulation, mitochondrial protection, and cellular homeostasis. (**c**) Summary of current clinical evidence in neurodevelopmental conditions.

N-acetylcysteine (NAC) is a precursor of glutathione with antioxidant and redox-modulating properties that has been investigated as a potential adjunctive intervention in neurodevelopmental disorders, particularly autism spectrum disorder (ASD). Oxidative stress, altered glutathione metabolism, glutamatergic dysregulation, neuroinflammation, and mitochondrial dysfunction have all been implicated to varying degrees in ASD pathophysiology, providing a biological rationale for investigating NAC in this population. NAC may influence several of these pathways through replenishment of intracellular glutathione, modulation of glutamatergic neurotransmission through the cystine–glutamate antiporter, and effects on oxidative and inflammatory processes. These neurobiological mechanisms and the available clinical evidence are summarized in Figure 5a–c [163,164,165,166,167,168,169,170,171,172,173,174,175,176,177,178,179,180,181,182,183,184,185,186,187,188]. Specifically, Figure 5a illustrates the major neurotransmitter pathways potentially modulated by N-acetylcysteine (*NAC*), while Figure 5b highlights the principal non-neurotransmitter mechanisms, including glutathione and redox regulation, anti-inflammatory and immune modulation, mitochondrial protection, and cellular homeostasis. Figure 5c provides an overview of the current clinical evidence regarding the potential therapeutic applications of NAC in neurodevelopmental conditions.

One of the earliest clinical reports was described by Ghanizadeh and Derakhshan (2012) [165], who documented behavioral improvements in a child with autism treated with oral NAC at approximately 800 mg/day. Subsequently, Hardan et al. (2012) [166], in a randomized controlled pilot trial, reported a greater reduction in irritability with NAC than with placebo in children with autistic disorder after 12 weeks of treatment, supporting its potential therapeutic utility in ASD-related behavioral dysregulation. Notably, participants with and without intellectual disability were eligible for inclusion; however, the study was not designed to evaluate treatment effects according to ID status.

Subsequent studies produced mixed but informative findings. Nikoo et al. (2015) [167] later evaluated NAC as an adjunct to risperidone in children and adolescents with ASD and reported greater reductions in irritability with combined NAC–risperidone treatment than with risperidone plus placebo. Because NAC was administered as adjunctive therapy, however, its independent contribution cannot be fully established. Wink et al. (2016) [168] demonstrated that NAC administration increased glutathione production and was generally well tolerated in youth with ASD, although no significant improvement in core social impairment was observed. Despite these mixed findings, NAC continued to attract interest because of its generally favorable safety profile and mechanistic rationale.

Additional clinical evidence has been reported in small observational studies. Pesko et al. (2020) [169] described NAC supplementation in a case series of four youths with ASD and severe irritability in a clinically complex inpatient population. Improvements in irritability were reported during treatment; however, the retrospective, uncontrolled design and very small sample preclude conclusions regarding efficacy. Quantitative evidence has provided cautiously favorable findings for selected behavioral outcomes. Lee et al. (2021) [170], in an updated systematic review and meta-analysis of five randomized controlled trials, reported improvements in overall Aberrant Behavior Checklist scores and pooled improvements in hyperactivity and irritability after 8–12 weeks of NAC supplementation. A significant improvement in social awareness was also observed, whereas no significant pooled effects were identified for repetitive behaviors. The authors considered NAC generally safe and tolerable but emphasized that additional evidence was required before its routine clinical use could be recommended.

More recent evidence reinforces the need for a balanced interpretation. A 2025 systematic review of antioxidant-based pharmacological interventions in ASD identified three randomized controlled trials evaluating NAC. Although improvements were reported for selected outcomes in some studies, including irritability, repetitive behavior, social cognition, and hyperactivity, results were inconsistent across trials; other controlled studies did not demonstrate significant differences in major behavioral outcomes. The review concluded that the available evidence remains insufficient to support antioxidant interventions as monotherapy for ASD [171].

Preclinical studies provide additional mechanistic support but should be distinguished from clinical evidence. Experimental work has reported effects of NAC on oxidative balance, inflammatory signaling, and behavioral phenotypes in animal models relevant to ASD. Zhang et al. (2023) [172], for example, reported attenuation of autism-like behaviors and changes in molecular pathways in a valproic acid-induced model, whereas Pangrazzi et al. (2024) [173] investigated NAC in a Shank3b-deficient model of syndromic ASD and reported effects on cerebellar immune and inflammatory alterations together with behavioral changes. Such findings may help identify potential biological targets and support investigation of NAC-mediated modulation of oxidative stress, glutamatergic signaling, mitochondrial dysfunction, immune activation, and neuroinflammation; however, they cannot establish clinical efficacy in humans. Typical NAC supplementation regimens in ASD studies generally range between approximately 900 and 2700 mg/day, usually administered in divided doses, over relatively short treatment periods of 8–12 weeks. NAC is generally safe and well tolerated, with the most commonly reported adverse effects including gastrointestinal discomfort, appetite changes, agitation, and mild behavioral activation, although available studies are insufficient to characterize long-term safety in neurodevelopmental populations. Moreover, the hypothesis that individuals with greater oxidative or glutathione abnormalities might preferentially respond to NAC remains of interest but requires prospective biomarker-stratified clinical validation [165,166,167,168,169,170,171,172,173].

Overall, the available evidence indicates that NAC has been clinically investigated in pediatric ASD, with the most consistent signals involving irritability and hyperactivity; however, findings across individual trials and behavioral domains remain heterogeneous, and current evidence does not establish NAC as a treatment for core ASD symptoms. Importantly, some ASD trials included participants with intellectual disability, but no clinical trials specifically designed to evaluate NAC efficacy in an ID-defined population were identified in our literature search. Consequently, the evidence should not be interpreted as establishing efficacy for intellectual disability itself or for neuropsychiatric manifestations across the broader ID population. Larger, adequately powered trials with standardized outcome measures, longer follow-up, and prospective evaluation of potential responder subgroups are warranted (Table 11 and Table 12).

#### 3.2.2. L-Carnitine and Acetyl-L-Carnitine

**Figure 6:**
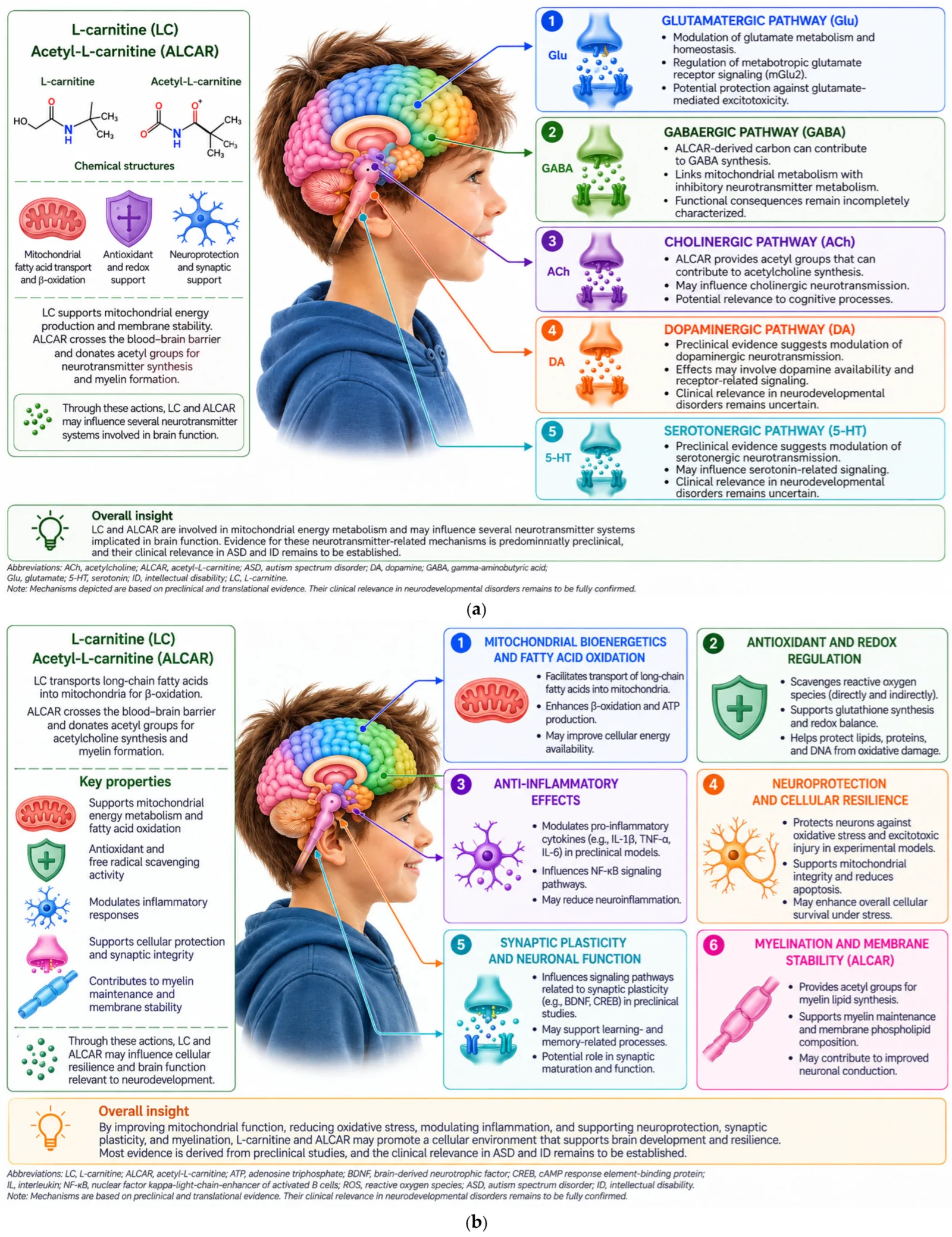

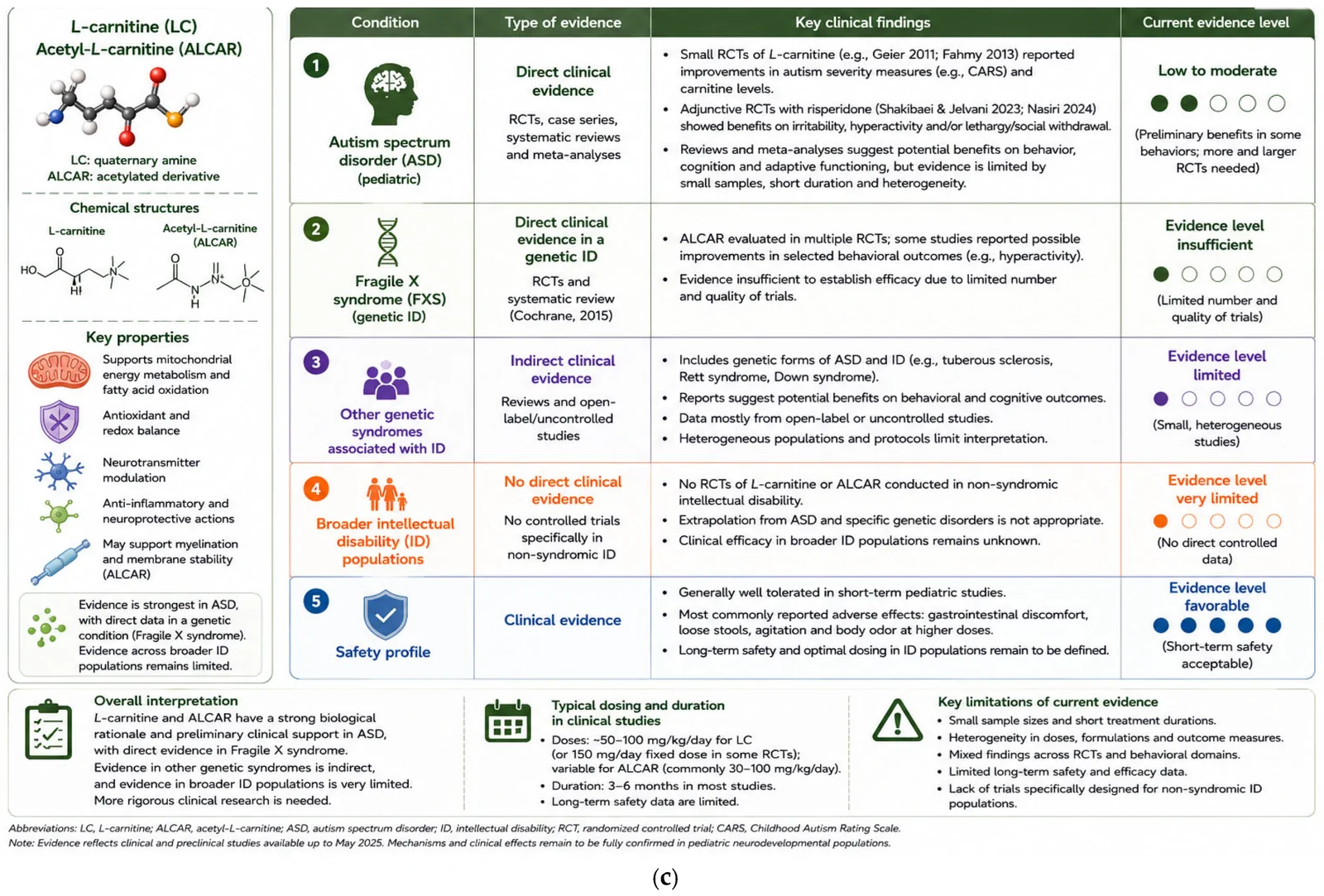
Neurobiological mechanisms and clinical evidence for L-Carnitine and ALCAR [189,190,191,192,193,194,195,196,197,198,199,200,201,202,203,204,205,206,207,208,209,210,211,212,213,214,215,216,217,218,219,220,221,222,223]. (**a**) Major neurotransmitter pathways potentially modulated by L-Carnitine and ALCAR. (**b**) Principal non-neurotransmitter mechanisms, including glutathione/redox regulation, anti-inflammatory and immune modulation, mitochondrial protection, and cellular homeostasis. (**c**) Summary of current clinical evidence in neurodevelopmental conditions.

L-carnitine and acetyl-L-carnitine (ALCAR) have been investigated in neurodevelopmental disorders, particularly autism spectrum disorder (ASD), because of their involvement in mitochondrial metabolism, fatty acid oxidation, cellular energy production, and redox homeostasis. L-carnitine facilitates the transport of long-chain fatty acids into mitochondria for β-oxidation, whereas ALCAR crosses the blood–brain barrier and may additionally influence acetyl-group availability, neurotransmitter metabolism, and neuronal function. These neurobiological mechanisms and the available clinical evidence are summarized in Figure 6a–c [189,190,191,192,193,194,195,196,197,198,199,200,201,202,203,204,205,206,207,208,209,210,211,212,213,214,215,216,217,218,219,220,221,222,223]. Specifically, Figure 6a illustrates the major neurotransmitter pathways potentially modulated by L-carnitine and acetyl-L-carnitine (ALCAR), while Figure 6b highlights the principal non-neurotransmitter mechanisms, including glutathione and redox regulation, anti-inflammatory and immune modulation, mitochondrial protection, and cellular homeostasis. Figure 6c provides an overview of the current clinical evidence regarding the potential therapeutic applications of L-carnitine and ALCAR in neurodevelopmental conditions. Altered mitochondrial function, fatty acid metabolism, and acylcarnitine profiles have been reported in subsets of individuals with ASD, providing a biological rationale for investigating *carnitine* supplementation, although these metabolic abnormalities are heterogeneous and are not present in all individuals with ASD (Table 13 and Table 14).

Clinical evidence in pediatric ASD includes several relatively small randomized controlled trials. Geier et al. (2011) [189] evaluated L-carnitine at 50 mg/kg/day for 3 months in children with ASD and reported improvements in selected measures of autism-related symptoms compared with placebo, including Childhood Autism Rating Scale (CARS) scores. However, the trial was limited by its small sample size, with 27 participants completing the study. Fahmy et al. (2013) [190] subsequently evaluated L-carnitine at 100 mg/kg/day for 6 months in 30 children with ASD and reported improvement in CARS scores together with increases in free and total carnitine levels. The relatively small sample and use of autism severity scales as the principal clinical outcome nevertheless limit the generalizability of these findings.

Evidence relevant to intellectual disability is also available from studies of Fragile X syndrome (FXS). Rueda et al. (2015) [191], in a Cochrane systematic review of randomized controlled trials of ALCAR in individuals with FXS, evaluated its effects on psychological, intellectual, and social functioning. Although some findings suggested possible effects on selected behavioral outcomes, including hyperactivity, the available evidence was insufficient to establish efficacy because of the limited number and methodological quality of the included studies. These findings are particularly relevant because FXS represents a well-established genetic cause of intellectual disability; however, they should not be generalized to the broader heterogeneous ID population. Ferreira and McKenna (2017) [192] later reviewed the neuroprotective mechanisms of acetyl-L-carnitine, supporting its role in mitochondrial bioenergetics, antioxidant defense, and modulation of glutamatergic neurotransmission. Adams et al. (2018) [193] evaluated a multicomponent nutritional intervention that included *carnitine* and reported improvements in several clinical and metabolic outcomes; however, the independent contribution of carnitine cannot be determined from this study.

Interest in *carnitine* metabolism increased following recognition of abnormal *acylcarnitine* profiles and mitochondrial dysfunction in ASD. Demarquoy et al. (2019) [194] summarized evidence showing reduced plasma L-carnitine levels and altered fatty acid metabolism in ASD populations, while Malaguarnera et al. (2019) [195], in a comprehensive review, concluded that both randomized trials and open-label studies suggest beneficial effects of carnitine administration on behavioral symptoms, cognition, and adaptive functioning in ASD, including forms associated with genetic syndromes and intellectual disability, although the evidence remained limited by small samples, heterogeneous populations, and differences in supplementation protocols.

Kępka et al. (2021) [196] further emphasized the relevance of altered carnitine metabolism, mitochondrial dysfunction, and *acylcarnitine* abnormalities in ASD, proposing serum/plasma carnitine and dried blood spot *acylcarnitine* analysis as potentially useful biomarkers for metabolic stratification and therapeutic monitoring, although their clinical utility for treatment selection or monitoring remains to be established. Nabi et al. (2023) [197] subsequently reviewed mitochondrial-targeted nutraceutical interventions in ASD and identified L-carnitine and acetyl-L-carnitine as promising metabolic therapies because of their antioxidant and neuroprotective effects. 

More recent randomized evidence has evaluated L-carnitine as an adjunct to risperidone. Shakibaei and Jelvani (2023) [198], in a randomized, double-blind, placebo-controlled adjunctive trial involving 50 children and adolescents with ASD, reported greater improvement in total Aberrant Behavior Checklist (ABC) scores and the lethargy/social-isolation subscale after 8 weeks of L-carnitine plus risperidone compared with risperidone plus placebo. Although within-group improvements were observed across additional ABC domains, significant between-group differences were more limited. Because L-carnitine was administered as adjunctive therapy, its independent therapeutic contribution cannot be established from this study. Nasiri et al. [199] subsequently conducted a randomized, double-blind trial involving 68 children with ASD, of whom 60 completed treatment. L-carnitine (150 mg/day) added to risperidone was associated with greater reductions in irritability and hyperactivity than risperidone plus placebo over 10 weeks, whereas differences were not observed for several other ABC-C domains. The authors themselves considered these findings preliminary and emphasized the need for larger and longer trials.

Preclinical evidence provides additional mechanistic support for ALCAR but should be interpreted separately from clinical findings. Experimental studies, including valproic acid-induced models of autism-like behavior, have investigated potential effects on oxidative balance, neuroinflammatory pathways, mitochondrial function, and behavioral phenotypes. Such observations may contribute to understanding the biological actions of carnitine derivatives but cannot establish efficacy in human neurodevelopmental disorders.

Recent secondary evidence continues to support a cautious interpretation. Persico et al. (2025) [200], in a systematic review of pediatric psychopharmacological interventions for ASD, identified L-carnitine among a small group of compounds for which controlled clinical evidence was considered sufficiently promising to warrant clinical interest. Nevertheless, the authors emphasized the need for caution, and the available evidence remains substantially less extensive than that supporting established pharmacological interventions.

Dosing has varied considerably across studies. Early ASD trials evaluated L-carnitine at approximately 50–100 mg/kg/day, whereas more recent adjunctive trials have used substantially lower fixed doses. L-carnitine has generally been reported as well tolerated in short-term studies, although gastrointestinal symptoms and other mild adverse effects have been described. Available evidence remains insufficient to characterize long-term safety and optimal dosing across heterogeneous neurodevelopmental populations. Overall, current evidence provides preliminary clinical support for L-carnitine in selected behavioral outcomes in pediatric ASD, while evidence for ALCAR in Fragile X syndrome remains insufficient to establish efficacy. Importantly, the latter represents direct investigation in a genetic disorder strongly associated with intellectual disability, but these findings should not be extrapolated to intellectual disability of other etiologies. Across the broader ID population, evidence remains limited, and larger adequately powered randomized controlled trials are required to determine clinical efficacy, optimal dosing, long-term safety, and whether metabolic or genetic subgroups are more likely to respond [189,190,191,192,193,194,195,196,197,198,199,200,201,202,203,204,205,206,207,208,209,210,211,212,213,214,215,216,217,218,219,220,221,222,223].

#### 3.2.3. Resveratrol

**Figure 7:**
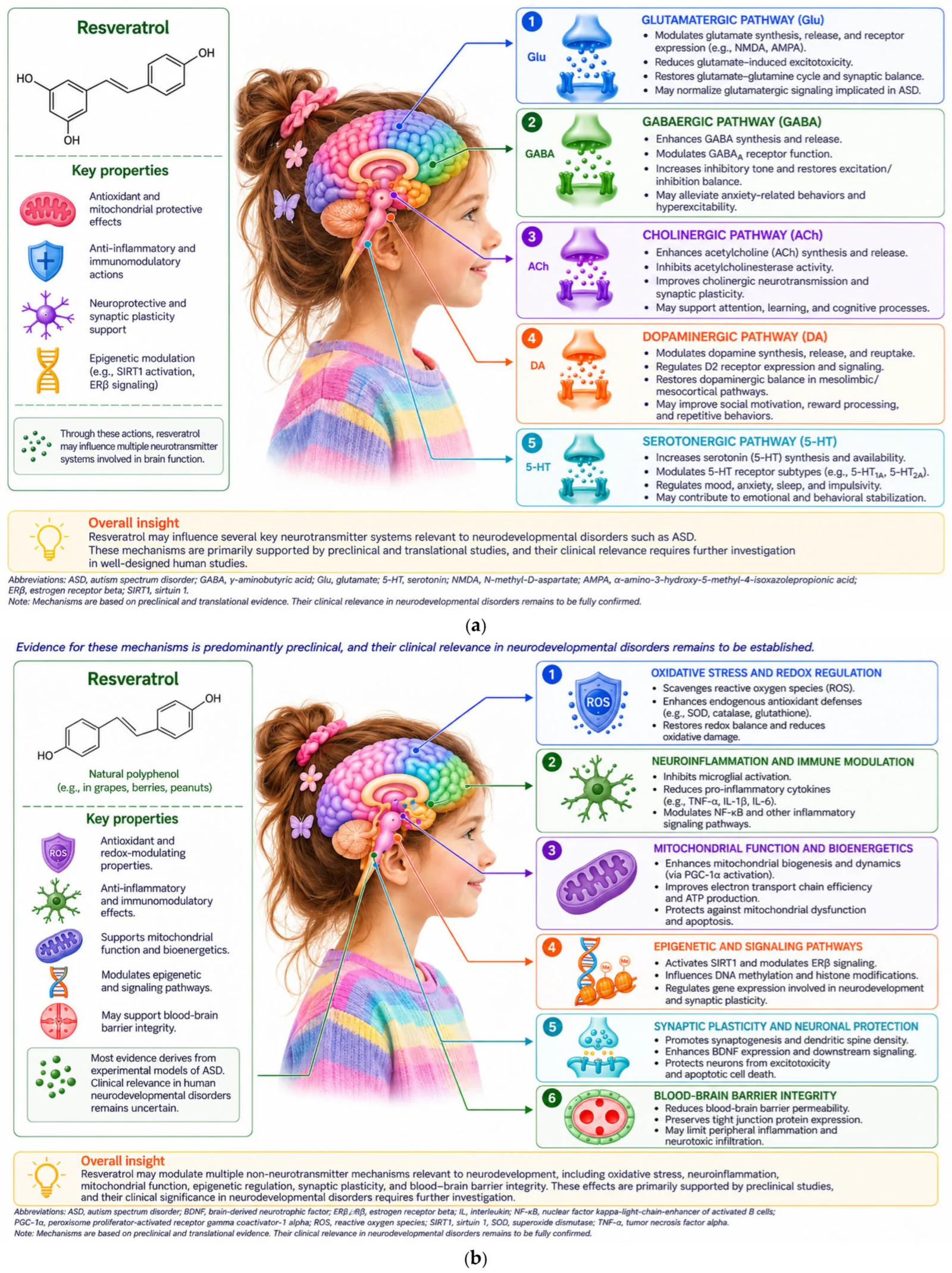

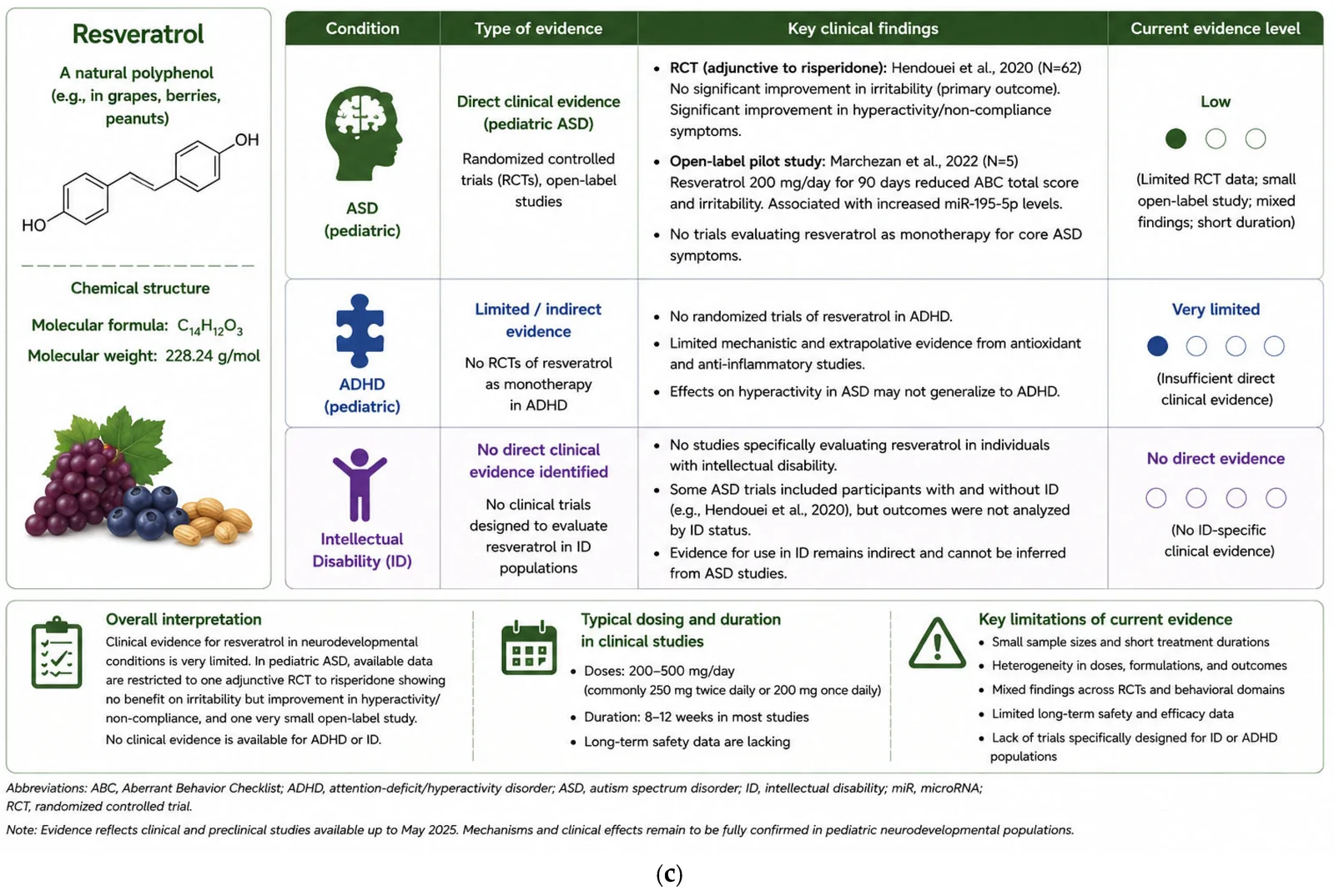
Neurobiological mechanisms and clinical evidence for Resveratrol [223,224,225,226,227,228,229,230,231,232,233,234,235,236,237,238,239,240,241]. (**a**) Major neurotransmitter pathways potentially modulated by Resveratrol. (**b**) Principal non-neurotransmitter mechanisms, including glutathione/redox regulation, anti-inflammatory and immune modulation, mitochondrial protection, and cellular homeostasis. (**c**) Summary of current clinical evidence in neurodevelopmental conditions.

Resveratrol is a naturally occurring polyphenol found in grapes, berries, and peanuts that has been investigated for its antioxidant, anti-inflammatory, mitochondrial, and neuroprotective properties. These neurobiological mechanisms and the available clinical evidence are summarized in Figure 7a–c [224,225,226,227,228,229,230,231,232,233,234,235,236,237,238,239,240,241]. Specifically, Figure 7a illustrates the major neurotransmitter pathways potentially modulated by resveratrol, while Figure 7b highlights the principal non-neurotransmitter mechanisms, including glutathione and redox regulation, anti-inflammatory and immune modulation, mitochondrial protection, and cellular homeostasis. Figure 7c provides an overview of the current clinical evidence regarding the potential therapeutic applications of resveratrol in neurodevelopmental conditions.In neurodevelopmental research, most evidence relevant to resveratrol derives from experimental models of autism spectrum disorder (ASD), whereas human clinical evidence remains limited. Proposed mechanisms include modulation of oxidative stress, neuroinflammatory signaling, mitochondrial function, synaptic plasticity, immune pathways, and blood–brain barrier integrity (Table 15 and Table 16).

One of the earliest and most influential preclinical studies was conducted by Bambini-Junior et al. (2014) [224], who demonstrated that prenatal administration of resveratrol prevented social interaction deficits in a valproic acid (VPA)-induced animal model of autism. Resveratrol-treated animals showed improved sociability and social preference behaviors, suggesting neuroprotective effects during critical stages of neurodevelopment. Subsequently, Bhandari et al. (2017) [225] reported that resveratrol restored autistic-like behaviors in propionic acid-induced ASD models through reduction in oxidative–nitrosative stress, mitochondrial dysfunction, TNF-α expression, and matrix metalloproteinase-9 activity.

Additional mechanistic studies further expanded these findings. Xie et al. (2018) [226] demonstrated that prenatal resveratrol administration ameliorated progestin-induced autism-like behaviors through activation of estrogen receptor-β (ERβ) signaling and epigenetic modulation, including DNA and histone demethylation. In the same period, Fontes-Dutra et al. (2018) [227] showed that resveratrol prevented sensory and behavioral abnormalities in experimental autism models, further supporting its effects on neuronal plasticity and sensory processing. Hidema et al. (2020) [228] additionally demonstrated that acute resveratrol administration improved social deficits in oxytocin receptor knockout mice, suggesting involvement of oxytocinergic signaling pathways relevant to ASD.

Human clinical evidence remains limited and has yielded mixed findings. Hendouei et al. (2020) [229], in a randomized placebo-controlled trial evaluating resveratrol as adjunctive therapy to risperidone in children with ASD, did not observe significant reductions in irritability overall; however, improvements were reported in hyperactivity and noncompliance symptoms. Marchezan et al. (2022) [230], in a small open-label pilot study, administered trans-resveratrol (200 mg/day for 90 days) to children with ASD and reported reductions in ABC total and irritability scores. Treatment was also associated with changes in circulating miR-195-5p, a microRNA implicated in inflammatory and immune-related pathways. However, the absence of a placebo-controlled group and the very small sample substantially limit causal interpretation. Recent reviews have summarized the growing body of evidence supporting the biological plausibility of resveratrol in ASD and related neurodevelopmental disorders. Malaguarnera et al. (2020) [231] highlighted resveratrol’s multimodal mechanisms involving antioxidant defense, cytokine modulation, mitochondrial protection, and neuroimmune regulation. More recently, Nikfarjam et al. (2026) [232] reviewed preventive and therapeutic applications of resveratrol in experimental and clinical ASD studies, emphasizing its ability to modulate oxidative stress, neurodevelopmental signaling pathways, neuroinflammation, and behavioral abnormalities. Overall, resveratrol has a substantial preclinical literature supporting antioxidant, anti-inflammatory, mitochondrial, and neuroprotective effects relevant to neurodevelopmental biology. However, human clinical evidence in ASD remains limited and has yielded mixed findings. In the available randomized adjunctive trial, resveratrol did not demonstrate superiority for the primary irritability outcome, although a potential benefit was observed for hyperactivity/non-compliance. Findings from the small open-label study should be interpreted cautiously because of the extremely limited sample size and absence of a control group. Recent reviews published in 2026 [14] similarly emphasize the predominantly preclinical nature of the evidence and the limited strength of current clinical data. No clinical evidence specifically establishing the efficacy of resveratrol in individuals with intellectual disability was identified in our literature search. Therefore, extrapolation from ASD and experimental models to the broader ID population remains uncertain, and adequately powered randomized controlled trials are required to determine clinical efficacy, optimal dosing, responder characteristics, and long-term safety [14,224,225,226,227,228,229,230,231,232,233,234,235,236,237,238,239,240,241].

#### 3.2.4. Phosphatidylserine

**Figure 8:**
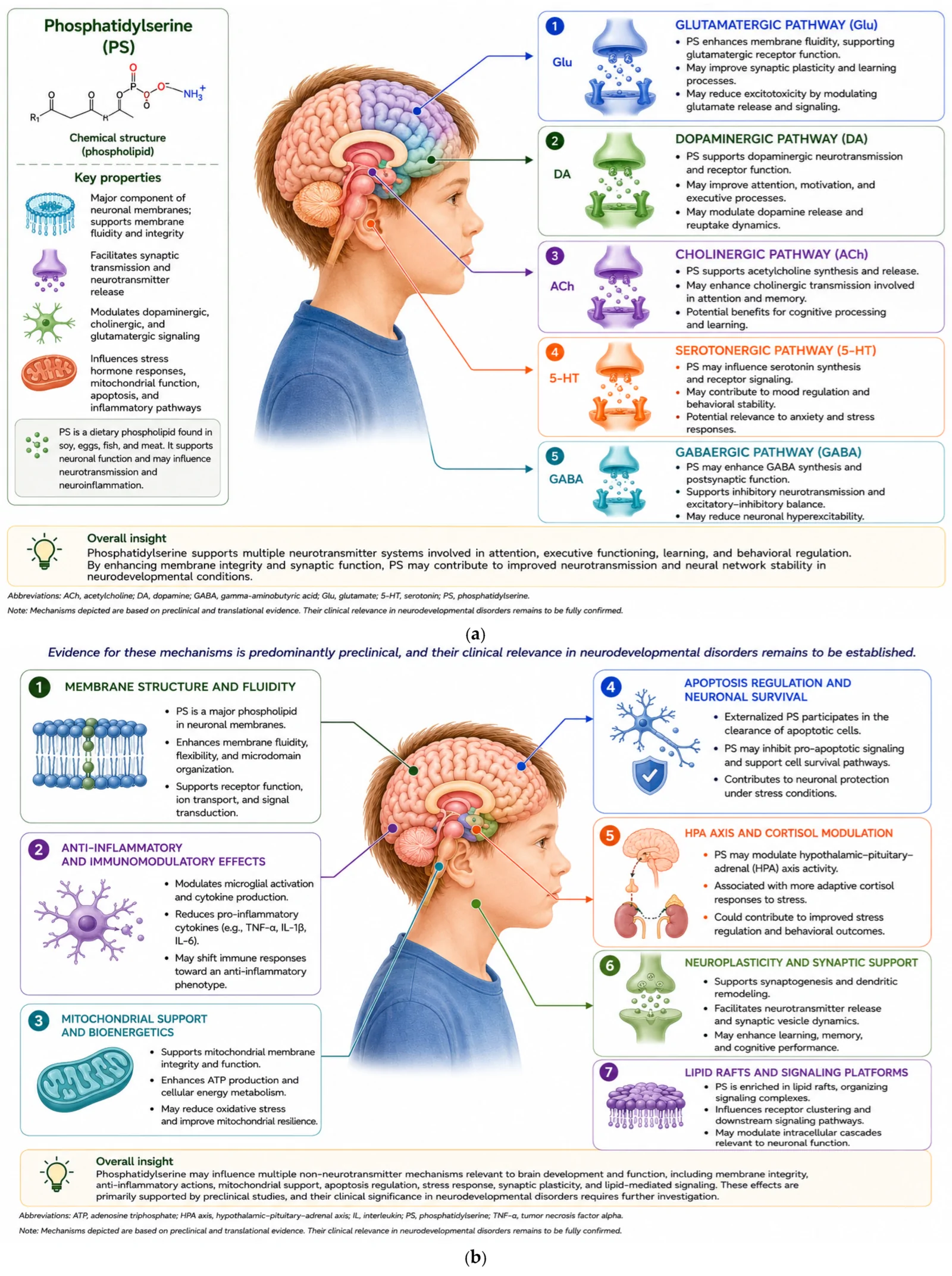

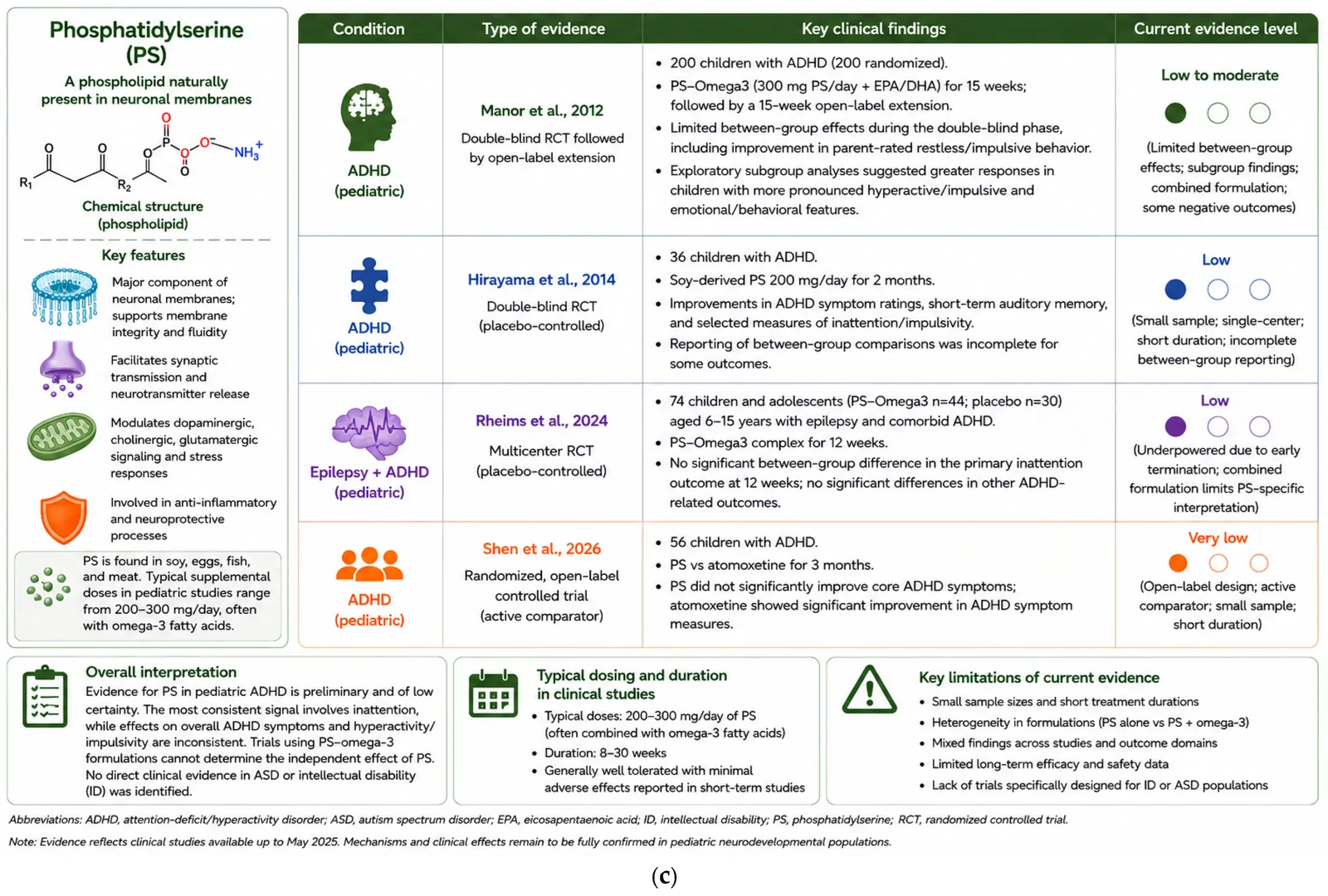
Neurobiological mechanisms and clinical evidence of Phosphatidylserine (PS) [242,243,244,245,246,247,248,249,250,251,252,253,254,255,256,257,258,259,260,261,262,263,264,265,266,267,268,269,270,271]. (**a**) Major neurotransmitter pathways potentially modulated by Phosphatidylserine (PS). (**b**) Principal non-neurotransmitter mechanisms, including glutathione/redox regulation, anti-inflammatory and immune modulation, mitochondrial protection, and cellular homeostasis. (**c**) Summary of current clinical evidence in neurodevelopmental conditions.

Phosphatidylserine (PS) is a phospholipid enriched in neuronal membranes and involved in membrane organization, intracellular signaling, and synaptic function. Experimental evidence has also implicated PS-dependent processes in apoptosis, inflammatory signaling, and neuronal homeostasis (Ma et al., 2022) [242]. These neurobiological mechanisms and the available clinical evidence are summarized in Figure 8a–c [242,243,244,245,246,247,248,249,250,251,252,253,254,255,256,257,258,259,260,261,262,263,264,265,266,267,268,269,270,271]. Specifically, Figure 8a illustrates the major neurotransmitter pathways potentially modulated by phosphatidylserine (PS), while Figure 8b highlights the principal non-neurotransmitter mechanisms, including glutathione and redox regulation, anti-inflammatory and immune modulation, mitochondrial protection, and cellular homeostasis. Figure 8c provides an overview of the current clinical evidence regarding the potential therapeutic applications of phosphatidylserine in neurodevelopmental conditions.In neurodevelopmental research, clinical investigation of PS has focused predominantly on attention-deficit/hyperactivity disorder (ADHD), whereas evidence in autism spectrum disorder (ASD) and intellectual disability (ID) remains limited or absent.

One of the earliest controlled studies investigating phosphatidylserine in neurodevelopmental disorders was conducted by Manor et al. (2012) [243], who evaluated phosphatidylserine enriched with omega-3 fatty acids (PS-Omega-3) in children with ADHD over 30 weeks. The authors reported reductions in ADHD symptoms, impulsivity, and behavioral dysregulation, particularly in subgroups characterized by hyperactivity and emotional dysregulation. However, the combined formulation prevents attribution of these findings specifically to PS, and subgroup findings require cautious interpretation.

Hirayama et al. (2014) [244], in a randomized double-blind placebo-controlled trial, subsequently administered soy-derived phosphatidylserine at approximately 200 mg/day for 2 months to children with ADHD and demonstrated improvements in attention, hyperactivity, impulsivity, short-term auditory memory, and mental performance with generally good tolerability and the absence of major adverse effects. Nevertheless, the small sample and incomplete reporting of between-group comparisons for some outcomes limit the strength of the conclusions.

Ma et al. (2022) [242] reviewed the role of phosphatidylserine in inflammation and central nervous system disorders, emphasizing its involvement in neuronal membrane stability, apoptosis regulation, neuroimmune signaling, and synaptic function. The authors highlighted that PS may modulate inflammatory responses and neuronal repair mechanisms potentially relevant to ASD and intellectual disability.

More recent systematic reviews have produced cautiously supportive conclusions. Bruton et al. (2021) [245], in a systematic review and meta-analysis of phosphatidylserine supplementation in pediatric ADHD, concluded that PS at doses of approximately 200–300 mg/day may reduce symptoms of inattention, although evidence for hyperactivity and overall ADHD symptom reduction remained less consistent. The authors emphasized the generally favorable safety profile of phosphatidylserine, but rated the overall quality of evidence as low and highlighted the need for additional adequately powered trials.

Recent investigations have additionally explored phosphatidylserine in children with epilepsy and comorbid ADHD. Rheims et al. (2024) [246], in a multicenter randomized placebo-controlled trial, evaluated phosphatidylserine enriched with omega-3 fatty acids in children with epilepsy-associated ADHD. Although the study was underpowered because of limited recruitment and did not demonstrate significant improvement in ADHD symptoms compared with placebo, the authors highlighted the importance of investigating phospholipid-based nutritional interventions in epilepsy-related neurodevelopmental disorders.

More recently, Shen et al. (2026) [247] conducted a randomized, open-label, controlled trial comparing phosphatidylserine with atomoxetine in children with ADHD. After 3 months of treatment, phosphatidylserine did not produce a statistically significant improvement in core ADHD symptoms, whereas atomoxetine was associated with significant symptom reduction. These findings further indicate that the clinical effects of phosphatidylserine on core ADHD symptoms remain uncertain and require confirmation in adequately blinded, placebo-controlled trials.

Emerging translational evidence also suggests possible benefits of phosphatidylserine in ASD-related cognitive dysfunction and stress regulation. Preliminary studies reported improvements in cognitive flexibility, attention, cortisol regulation, and behavioral symptoms in children with mild or high-functioning ASD following phosphatidylserine supplementation, although available evidence remains limited and heterogeneous. These mechanisms provide biological context for further investigation but should not be interpreted as evidence of therapeutic efficacy in neurodevelopmental disorders. 

Typical phosphatidylserine supplementation regimens in pediatric neurodevelopmental studies generally range between approximately 200 and 300 mg/day, sometimes combined with omega-3 fatty acids. PS supplementation is generally considered safe and well tolerated, with minimal adverse effects reported across studies. Overall, the clinical evidence for PS in pediatric ADHD is preliminary and of low certainty, with the most consistent signal involving inattention rather than global ADHD symptoms or hyperactivity/impulsivity. Evidence from combined PS–omega-3 formulations cannot establish the independent contribution of PS. Moreover, no direct clinical evidence supporting PS supplementation specifically in individuals with ID was identified, limiting extrapolation to ID-associated neuropsychiatric conditions [242,243,244,245,246,247,248,249,250,251,252,253,254,255,256,257,258,259,260,261,262,263,264,265,266,267,268,269,270,271] (Table 17 and Table 18).

#### 3.2.5. Coenzyme Q10

**Figure 9:**
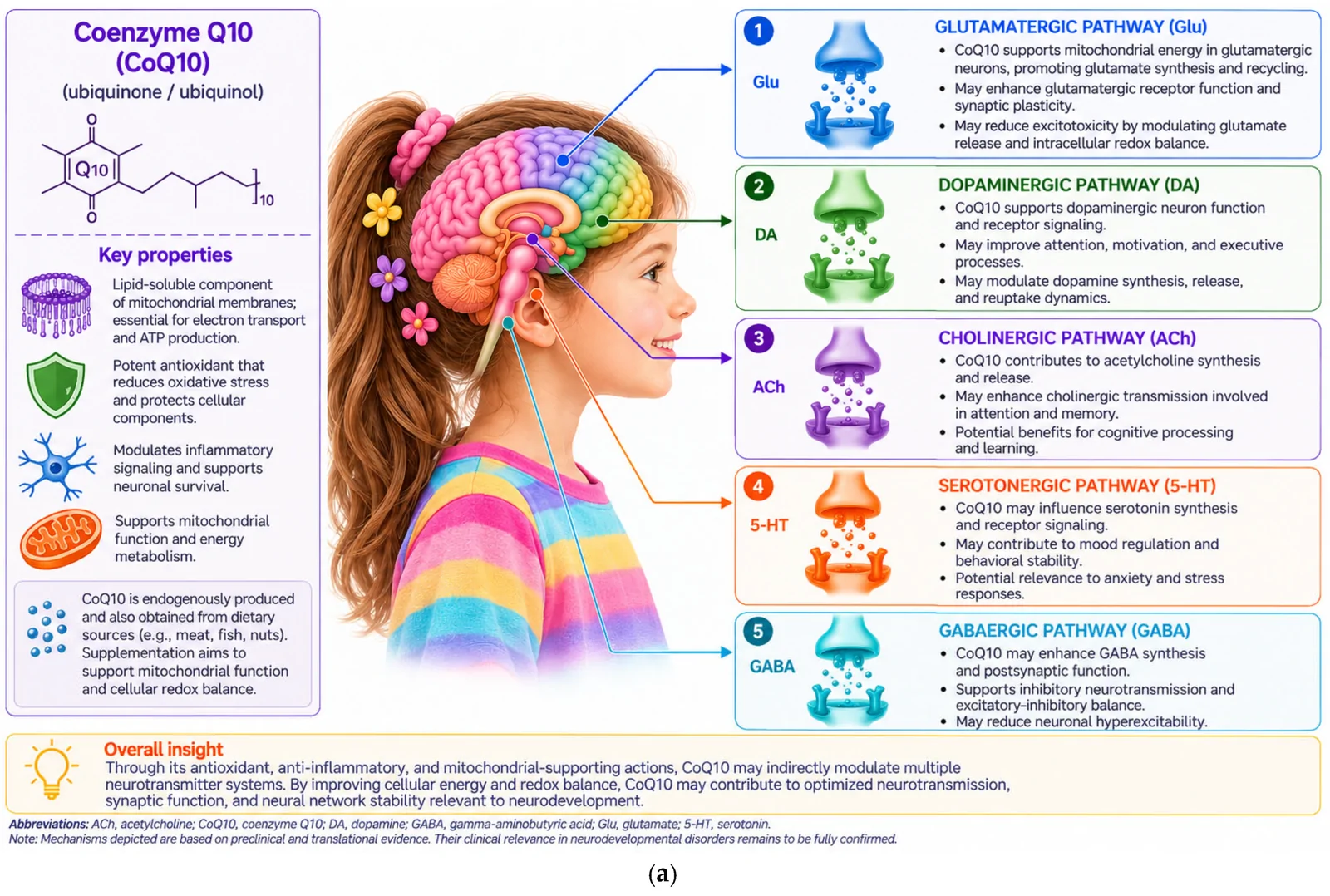

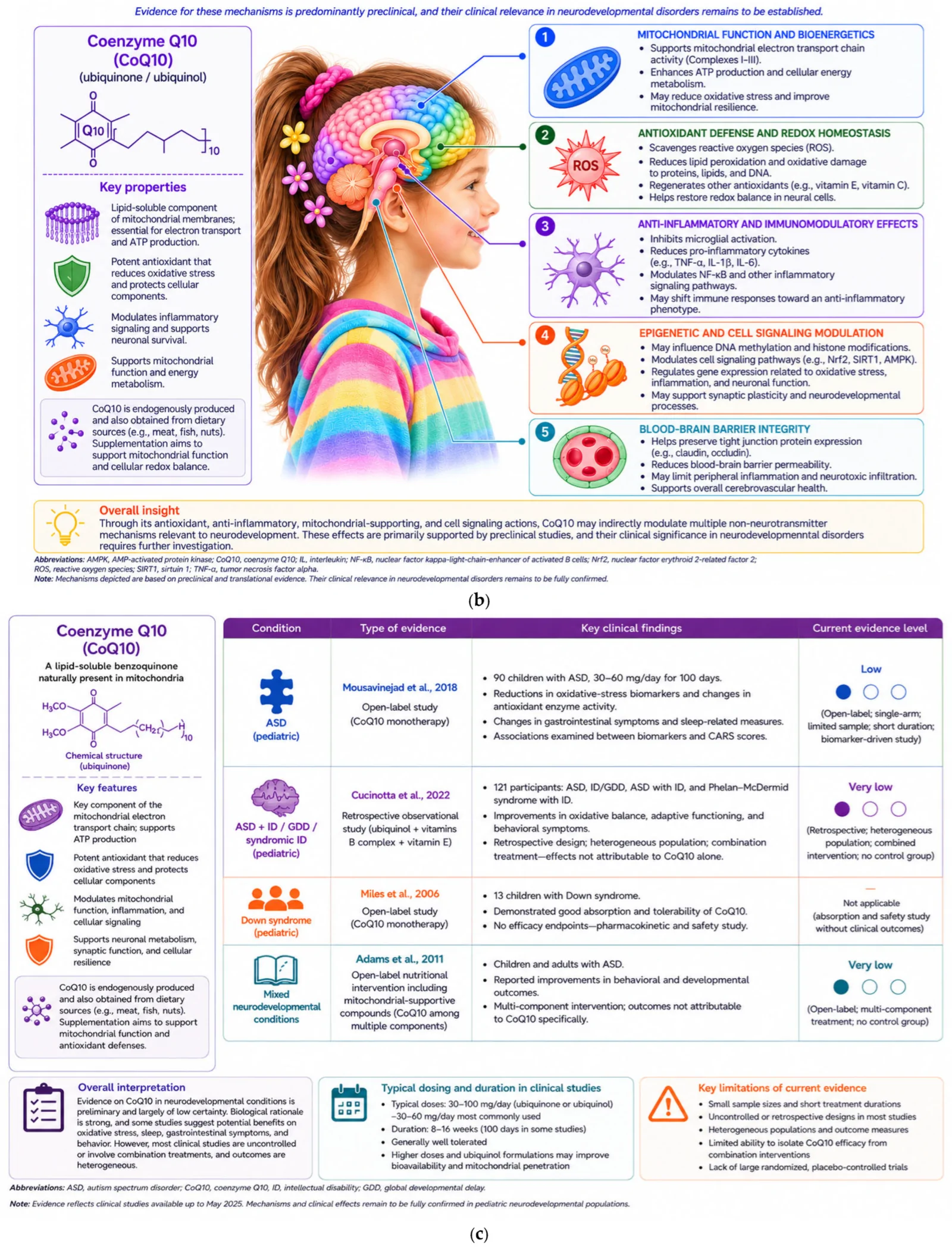
Neurobiological mechanisms and clinical evidence of Coenzyme Q10 [272,273,274,275,276,277,278,279,280,281,282,283,284,285,286,287,288,289,290,291,292,293,294,295,296,297,298,299,300]. (**a**) Major neurotransmitter pathways potentially modulated by Coenzyme Q10. (**b**) Principal non-neurotransmitter mechanisms, including glutathione/redox regulation, anti-inflammatory and immune modulation, mitochondrial protection, and cellular homeostasis. (**c**) Summary of current clinical evidence in neurodevelopmental conditions.

Coenzyme Q10 (CoQ10), particularly in its reduced form ubiquinol, has been investigated in several neurodevelopmental conditions, particularly ASD, because of its role in mitochondrial bioenergetics, oxidative stress regulation, antioxidant defense, and neuronal metabolism. These neurobiological mechanisms and the available clinical evidence are summarized in Figure 9a–c [272,273,274,275,276,277,278,279,280,281,282,283,284,285,286,287,288,289,290,291,292,293,294,295,296,297,298,299,300]. Specifically, Figure 9a illustrates the major neurotransmitter pathways potentially modulated by Coenzyme Q10 (CoQ10), while Figure 9b highlights the principal non-neurotransmitter mechanisms, including glutathione and redox regulation, anti-inflammatory and immune modulation, mitochondrial protection, and cellular homeostasis. Figure 9c provides an overview of the current clinical evidence regarding the potential therapeutic applications of CoQ10 in neurodevelopmental conditions. Mitochondrial dysfunction, impaired oxidative phosphorylation, glutathione depletion, and increased oxidative stress have repeatedly been reported in ASD populations, supporting the biological rationale for CoQ10 supplementation. CoQ10 acts as an electron carrier within the mitochondrial respiratory chain and additionally exerts antioxidant, anti-inflammatory, and neuroprotective effects that may influence synaptic plasticity, neuronal signaling, and neurodevelopment [272,273,274,275,276,277,278,279,280,281,282,283,284,285,286,287,288,289,290,291,292,293,294,295,296,297,298,299,300].

One of the earliest investigations relevant to neurodevelopmental disorders was conducted by Miles et al. (2006) [275], who evaluated CoQ10 absorption and tolerability in children with Down syndrome and demonstrated good safety and bioavailability profiles. Interest in mitochondrial-targeted interventions subsequently increased following recognition of mitochondrial dysfunction in ASD. Adams et al. (2011) [276], in a nutritional intervention study including mitochondrial-supportive compounds, reported improvements in behavioral and developmental outcomes in children and adults with ASD.

Tiano et al. (2011) [273] investigated the effects of CoQ10 supplementation in a double-blind, randomized, placebo-controlled trial involving children with Down syndrome, a genetic condition frequently associated with intellectual disability and mitochondrial oxidative imbalance. Supplementation with CoQ10 at 4 mg/kg/day for six months increased circulating CoQ10 concentrations and showed limited protective effects against oxidative DNA damage, although no significant overall improvement in DNA oxidation was demonstrated at the patient level. These findings provided early clinical evidence supporting the feasibility of CoQ10-based antioxidant interventions in neurodevelopmental conditions, while highlighting the need for further studies to establish clinically meaningful benefits. 

Interest in oxidative stress mechanisms further expanded in subsequent years. El-Ansary et al. (2016) [277] demonstrated that combined antioxidant and CoQ10-related metabolic interventions improved oxidative stress biomarkers in Young male Western albino rats, while Elham Mousavinejad et al. (2016) [278] reviewed evidence linking mitochondrial dysfunction, oxidative imbalance, and reduced CoQ10 concentrations in ASD populations and in 2018 subsequently showed that CoQ10 supplementation at doses of approximately 30–60 mg/day for 100 days may have reduced oxidative stress markers, decreased antioxidant enzyme activity, and improved gastrointestinal symptoms and sleep disorders in children with ASD. The study additionally demonstrated correlations between oxidative stress biomarkers and Childhood Autism Rating Scale (CARS) scores.

Demarquoy et al. (2019) [194] and subsequent metabolic reviews emphasized that mitochondrial dysfunction and oxidative imbalance may characterize biologically vulnerable ASD subgroups potentially responsive to mitochondrial-supportive therapies such as CoQ10. Burke et al. (2020) [279], in a comprehensive review of oxidative stress in ASD and intellectual/developmental disabilities, further highlighted CoQ10 as biologically plausible antioxidant intervention for neurodevelopmental disorders. 

More recent studies continued to support this therapeutic rationale. Cucinotta et al. (2022) [272], in a clinical study evaluating ubiquinol combined with vitamins B and E in ASD, reported improvements in oxidative balance, adaptive functioning, and behavioral symptoms following metabolic support therapy. Recent translational reviews additionally emphasized the role of CoQ10 in regulating neuroinflammation, mitochondrial respiration, glutathione metabolism, and synaptic function in ASD and related neurodevelopmental disorders.

Typical CoQ10 supplementation regimens in ASD studies generally range from approximately 30 to 100 mg/day, although higher doses and ubiquinol formulations may improve bioavailability and mitochondrial penetration. CoQ10 is generally considered safe and well tolerated, with mild gastrointestinal discomfort representing the most commonly reported adverse effect. Overall, available evidence provides a biological rationale and preliminary clinical support for CoQ10-based metabolic interventions in neurodevelopmental disorders. Evidence includes ASD populations as well as individuals with ID/GDD and syndromic ID; however, direct evidence specifically attributable to CoQ10 remains limited. Several studies are uncontrolled or involve combined nutritional formulations, and clinical outcomes are heterogeneous. Larger randomized, placebo-controlled trials are therefore required to determine whether CoQ10 provides clinically meaningful benefits, identify potential responder subgroups, and establish optimal dosing and long-term safety [194,272,273,274,275,276,277,278,279,280,281,282,283,284,285,286,287,288,289,290,291,292,293,294,295,296,297,298,299,300] (Table 19 and Table 20).

### 3.3. Micronutrients and Probiotics

#### 3.3.1. Omega-3 Polyunsaturated Fatty Acids (PUFAs)

**Figure 10:**
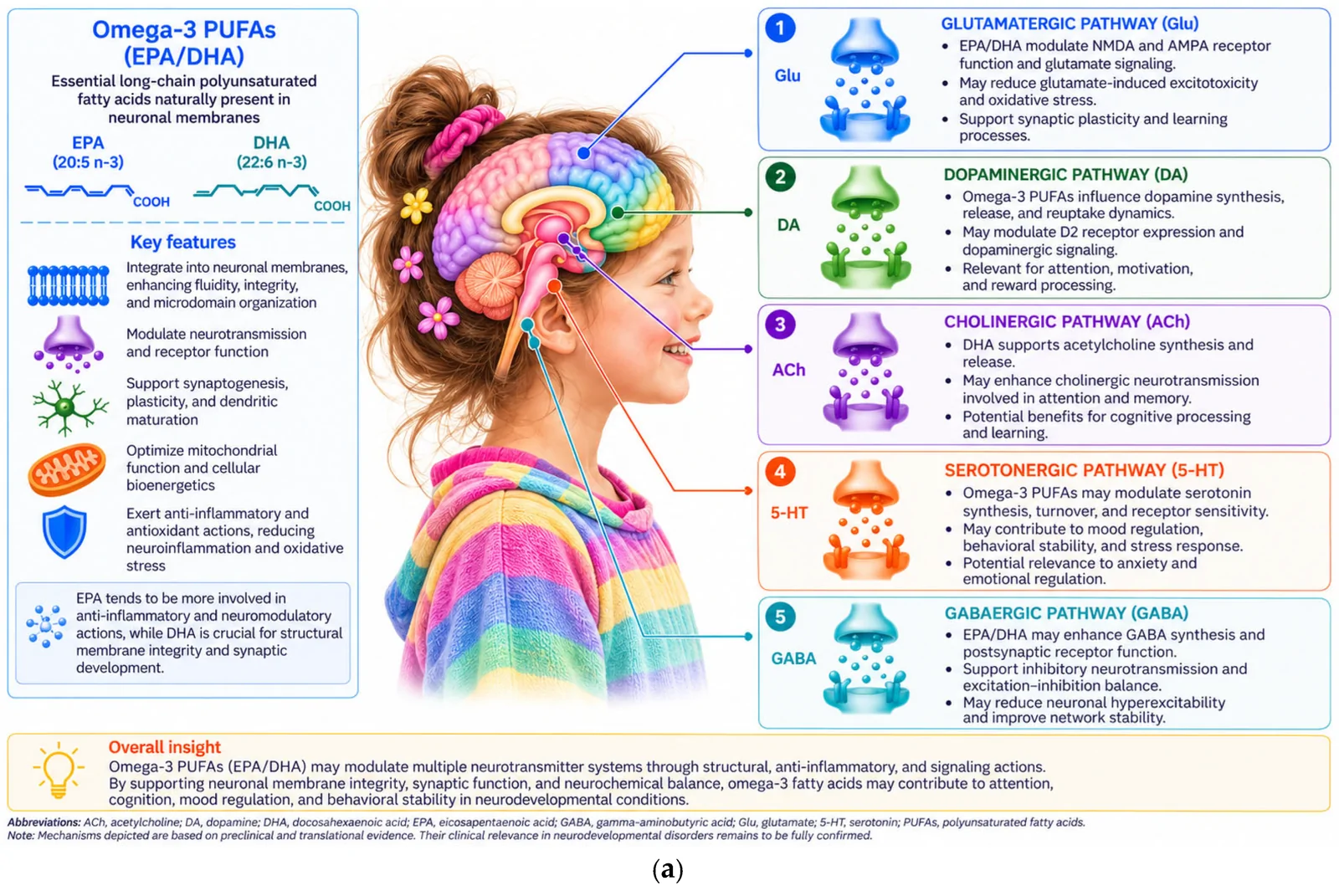

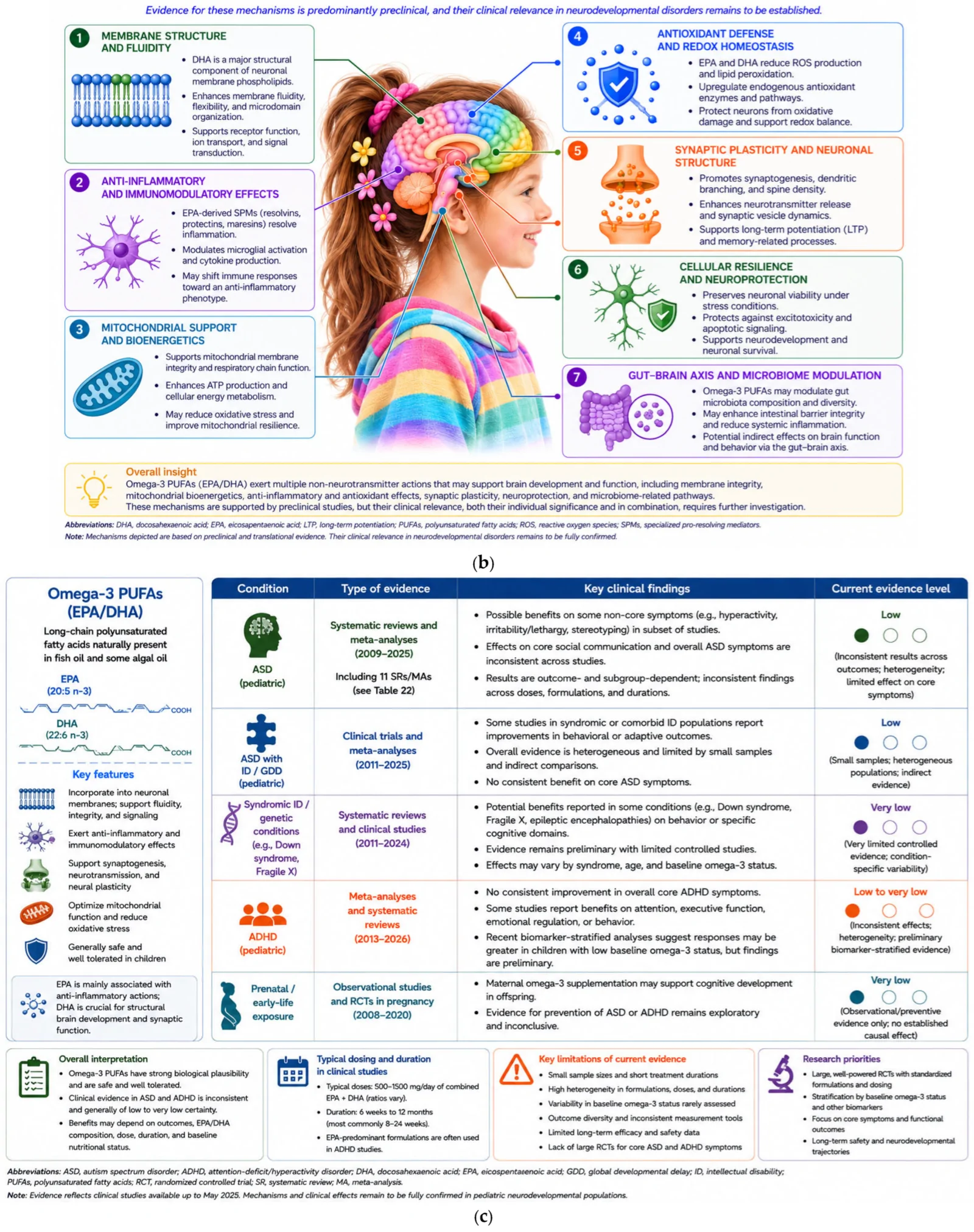
Neurobiological mechanisms and clinical evidence of Omega-3 [301,302,303,304,305,306,307,308,309,310,311,312,313,314,315,316,317,318,319,320,321,322,323,324,325,326,327,328,329,330,331,332,333,334,335,336,337,338,339,340,341,342,343,344,345]. (**a**) Major neurotransmitter pathways potentially modulated by Omega-3. (**b**) Principal non-neurotransmitter mechanisms, including glutathione/redox regulation, anti-inflammatory and immune modulation, mitochondrial protection, and cellular homeostasis. (**c**) Summary of current clinical evidence in neurodevelopmental conditions.

Omega-3 polyunsaturated fatty acids (PUFAs), particularly docosahexaenoic acid (DHA) and eicosapentaenoic acid (EPA), have been investigated in neurodevelopmental disorders associated with intellectual disability, especially autism spectrum disorder (ASD) and attention-deficit/hyperactivity disorder (ADHD). DHA represents a major structural component of neuronal membranes and is essential for synaptogenesis, membrane fluidity, myelination, and cortical development, whereas EPA appears to exert anti-inflammatory and immunomodulatory effects through the modulation of cytokine production, microglial activation, and specialized pro-resolving mediators (SPMs). Omega-3 fatty acids may additionally regulate serotoninergic and dopaminergic neurotransmission, increase brain-derived neurotrophic factor (BDNF) expression, optimize mitochondrial function, and mitigate oxidative stress and neuroinflammation—all core mechanisms implicated in neurodevelopmental conditions [301,302,303,304,305,306,307,308,309,310,311,312,313,314,315,316,317,318,319,320,321,322,323,324,325,326,327,328,329,330,331,332,333,334,335,336,337,338,339,340,341,342,343,344,345] (Table 21 and Table 22). These neurobiological mechanisms and the available clinical evidence are summarized in Figure 10a–c [301,302,303,304,305,306,307,308,309,310,311,312,313,314,315,316,317,318,319,320,321,322,323,324,325,326,327,328,329,330,331,332,333,334,335,336,337,338,339,340,341,342,343,344,345]. Specifically, Figure 10a illustrates the major neurotransmitter pathways potentially modulated by omega-3 polyunsaturated fatty acids (PUFAs), particularly DHA and EPA, while Figure 10b highlights the principal non-neurotransmitter mechanisms, including glutathione and redox regulation, anti-inflammatory and immune modulation, mitochondrial protection, and cellular homeostasis. Figure 10c provides an overview of the current clinical evidence regarding the potential therapeutic applications of omega-3 PUFAs in neurodevelopmental conditions.

The historical evolution of this therapeutic rationale reveals a progressive methodological refinement. Early systematic reviews, most notably the foundational synthesis by Bent et al. (2009) [302] and the subsequent evaluation by James et al. (2011) [303], initially concluded that evidence supporting omega-3 supplementation in ASD remained limited due to small sample sizes and highly heterogeneous methodologies, though preliminary trends toward the mitigation of hyperactivity and stereotyped behaviors were documented [299]. Bent et al. (2011) [304] conducted a pilot randomized controlled trial evaluating a standardized dose of 1.3 g/day of omega-3 fatty acids over 12 weeks in children with ASD. Although some changes in hyperactivity-related measures were observed, the small sample limited definitive conclusions. To address the recruitment boundaries of traditional designs, Bent et al. (2014) [305] conducted an internet-based randomized controlled trial involving 57 children with ASD; omega-3 supplementation was generally well tolerated, but the between-group difference in hyperactivity was not statistically significant compared to the parallel placebo group. Concurrently, broader biochemical evaluations suggest that children with ASD display altered plasma fatty acid profiles, characterized by depleted DHA/EPA stores and elevated omega-6/omega-3 ratios that could be associated with clinical symptom severity, providing a metabolic rationale for further investigation without establishing therapeutic efficacy.

More recent clinical data and meta-analyses have provided cautiously supportive evidence. Cheng et al. (2017) [306] reported modest but statistically significant improvements in hyperactivity, lethargy, and stereotyped behaviors in children with ASD following omega-3 regimens, while Horvath et al. (2017) [307] confirmed excellent compound tolerability and safety profiles despite noting limited impacts on core social communication deficits. Furthermore, Doaei et al. (2021) [308] reported significant improvements in social communication, stereotyped behaviors, and total GARS scores following omega-3 supplementation compared with placebo, whereas the social interaction subscale did not significantly improve, while de Andrade Wobido et al. (2022) [309] reported improvements in selected behavioral outcomes following combined omega-3 and omega-6 supplementation, although the combined formulation prevents attribution of these effects specifically to omega-3.

Evidence has also been extensively investigated in ADHD. Liu et al. (2023) [310], in a comprehensive systematic meta-analysis of pooled pediatric datasets, suggested that omega-3 supplementation does not consistently improve overall core ADHD symptoms, although effects on selected behavioral and cognitive outcomes have been reported in some studies. Differences in EPA/DHA composition, treatment duration, baseline fatty-acid status, and participant characteristics may contribute to the heterogeneity of treatment effects. EPA-predominant formulations have been proposed as potentially relevant to behavioral outcomes, but the optimal formulation and the clinical characteristics of potential responders remain uncertain.

Prenatal and early-life omega-3 exposure has additionally been investigated in relation to long-term neurodevelopmental outcomes. Donovan et al. (2020) [311] reported that maternal omega-3 supplementation during pregnancy may favorably influence cognitive development and mental processing scores in offspring, although preventive evidence regarding specific ASD or ADHD phenotypes remains exploratory and inconclusive. Emerging translational evidence also suggests potential clinical benefits in other genetic and neurological conditions associated with intellectual disability, including Down syndrome, epilepsy, and Fragile X syndrome, although available controlled datasets remain limited.

Typical supplementation regimens in pediatric neurodevelopmental literature range from approximately 500 to 1500 mg/day of combined EPA and DHA, with treatment durations varying between 6 weeks and 12 months. Omega-3 fatty acids are generally considered safe and well tolerated; however, documented minor adverse effects include transient gastrointestinal discomfort, a fishy aftertaste, and, at exceptionally high doses, a theoretical increase in bleeding risk, warranting caution in children receiving concurrent anticoagulant or antiplatelet therapies. High variability in raw oil formulations, EPA-to-DHA ratios, intervention durations, baseline nutritional status, and individual dietary habits likely explains the notable heterogeneity observed across contemporary clinical trials. Overall, omega-3 fatty acids have a well-established biological role in neuronal membrane structure and provide plausible mechanisms relevant to neurodevelopment, including modulation of inflammatory signaling, synaptic function, and neuronal homeostasis. Clinical evidence in ASD and ADHD, however, remains heterogeneous, with inconsistent effects across behavioral and cognitive outcomes. Differences in EPA/DHA composition, dose, treatment duration, baseline nutritional status, and study populations may contribute to this variability. Larger, adequately powered and well-standardized randomized controlled trials are required to determine whether specific formulations or biologically defined subgroups derive clinically meaningful benefit [301,302,303,304,305,306,307,308,309,310,311,312,313,314,315,316,317,318,319,320,321,322,323,324,325,326,327,328,329,330,331,332,333,334,335,336,337,338,339,340,341,342,343,344,345].

#### 3.3.2. Probiotics and Microbiota-Targeted Interventions

**Figure 11:**
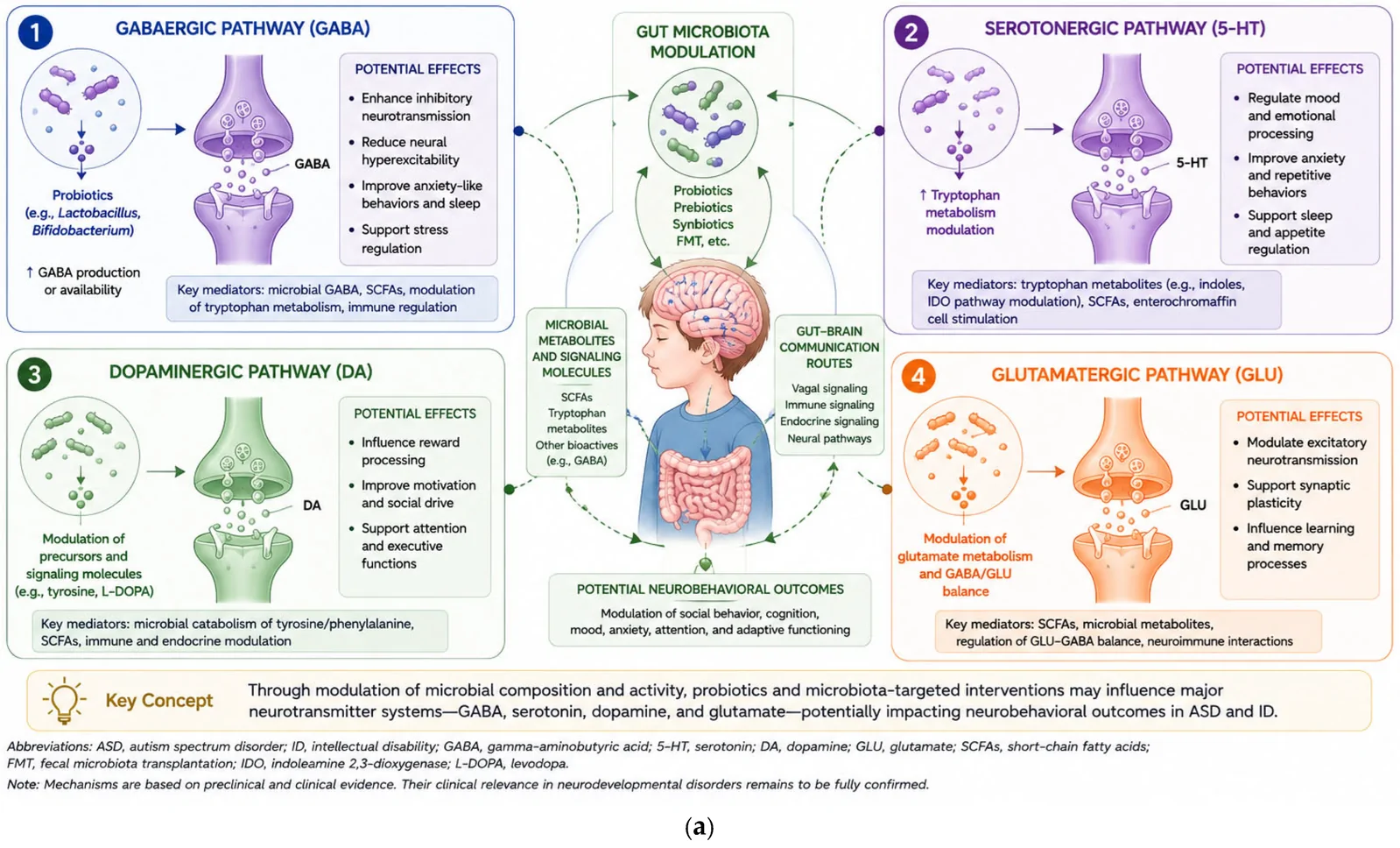

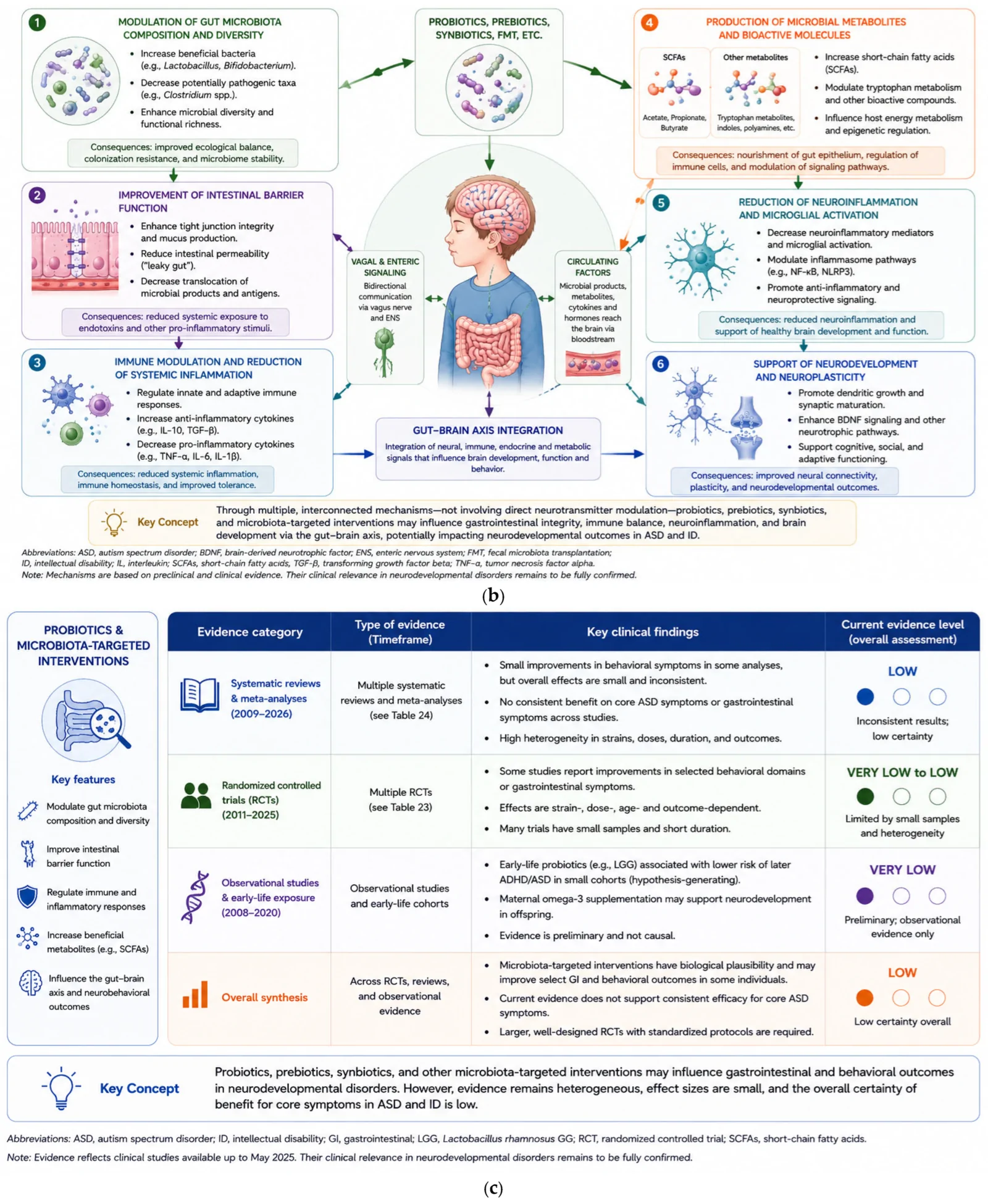
Neurobiological mechanisms and clinical evidence of probiotics [345,346,347,348,349,350,351,352,353,354,355,356,357,358,359,360,361,362,363,364,365,366,367,368,369,370,371,372,373,374,375,376,377,378,379,380,381,382,383,384,385,386,387,388,389,390,391,392,393,394,395,396,397,398,399,400]. (**a**) Major neurotransmitter pathways potentially modulated by probiotics. (**b**) Principal non-neurotransmitter mechanisms, including glutathione/redox regulation, anti-inflammatory and immune modulation, mitochondrial protection, and cellular homeostasis. (**c**) Summary of current clinical evidence in neurodevelopmental conditions.

Probiotics and other gut microbiota-targeted interventions have received increasing attention in neurodevelopmental disorders, particularly autism spectrum disorder (ASD), because of growing evidence implicating the microbiota–gut–brain axis in gastrointestinal, immune, metabolic, and neurobehavioral processes. These neurobiological mechanisms and the available clinical evidence are summarized in Figure 11a–c [346,347,348,349,350,351,352,353,354,355,356,357,358,359,360,361,362,363,364,365,366,367,368,369,370,371,372,373,374,375,376,377,378,379,380,381,382,383,384,385,386,387,388,389,390,391,392,393,394,395,396,397,398,399,400]. Specifically, Figure 11a illustrates the major neurotransmitter pathways potentially modulated by probiotics, while Figure 11b highlights the principal non-neurotransmitter mechanisms, including glutathione and redox regulation, anti-inflammatory and immune modulation, mitochondrial protection, and cellular homeostasis. Figure 11c provides an overview of the current clinical evidence regarding the potential therapeutic applications of probiotics in neurodevelopmental conditions. Studies in children with ASD have reported alterations in gut microbial composition and diversity, gastrointestinal dysfunction, intestinal permeability, immune signaling, and inflammatory pathways, although findings are heterogeneous and no single ASD-specific microbiota profile has been established (Table 23 and Table 24). These observations provide a biological rationale for investigating probiotics, prebiotics, synbiotics, and other microbiota-targeted interventions, but do not by themselves establish therapeutic efficacy [346,347,348,349,350,351,352,353,354,355,356,357,358,359,360,361,362,363,364,365,366,367,368,369,370,371,372,373,374,375,376,377,378,379,380,381,382,383,384,385,386,387,388,389,390,391,392,393,394,395,396,397,398,399,400].

Kang et al. (2017) [347], in an open-label study of microbiota transfer therapy (MTT), reported improvements in gastrointestinal symptoms and ASD-related behavioral measures that persisted during follow-up. However, the small sample and absence of a placebo-controlled group substantially limit causal interpretation. Subsequent clinical studies investigated specific probiotic formulations. Shaaban et al. (2018) [348] reported improvements in gastrointestinal symptoms and behavioral measures in a small open-label cohort of children with ASD, while Liu et al. (2019) [349] reported that *Lactobacillus plantarum* PS128 was associated with improvements in opposition/defiance behaviors, with effects on broader ADHD-related symptoms observed particularly among younger participants aged 7–12 years. Sanctuary et al. (2019) [350] reported changes in gastrointestinal and behavioral measures following an intervention combining probiotics with bovine colostrum; however, the very small sample and combined formulation preclude attribution of these findings specifically to probiotics. Arnold et al. (2019) [351], in a small placebo-controlled crossover pilot trial, found no significant differences in the primary gastrointestinal quality-of-life or anxiety outcomes, although a parent-selected gastrointestinal target symptom improved during probiotic treatment.

Increasing attention has also focused on strain-specific psychobiotic effects. Pärtty et al. (2015) [352], in a long-term follow-up of an early-life randomized probiotic intervention, reported ADHD and/or Asperger syndrome in 6 of 35 children originally assigned to placebo and none of 40 children assigned to *Lactobacillus rhamnosus GG*. Although intriguing, the small follow-up sample, low number of diagnostic events, and long interval between intervention and neurodevelopmental assessment mean that these findings should be considered hypothesis-generating rather than evidence of a preventive effect. Experimental studies further suggested that Lactobacillus reuteri may improve social deficits and oxytocin signaling in ASD animal models. *Bifidobacterium longum* has also been investigated as a potential psychobiotic strain; several studies reported increased abundance of Bifidobacterium species associated with reductions in Clostridium populations and improvements in gastrointestinal and behavioral symptoms in ASD (Wang et al., 2020) [353]. More recently, Li et al. (2025) [354] demonstrated that Bifidobacterium longum subsp. longum CCFM1077 significantly reduced Childhood Autism Rating Scale scores and anxiety-related symptoms in children with ASD. Synbiotic approaches combining probiotics with prebiotics such as galacto-oligosaccharides (GOS) have also shown encouraging effects on microbiota composition and metabolic activity (Duque et al., 2021) [355].

Despite promising findings, systematic reviews and meta-analyses have reported heterogeneous results. Ng et al. (2019) [356] concluded that evidence supporting probiotics in ASD remained limited because of small sample sizes, heterogeneous strains, and inconsistent methodologies. Song et al. (2022) [357], in a meta-analysis of controlled trials, found no significant improvements in overall ASD severity or gastrointestinal symptoms, although some subgroup analyses suggested benefits in selected behavioral domains. Patel (2022) [358] similarly emphasized methodological heterogeneity across studies, including differences in probiotic strains, dosage, treatment duration, and outcome measures. More recent meta-analyses have provided cautiously supportive evidence. He et al. (2023) [359] [found that probiotic blends may improve behavioral symptoms in subgroup analyses despite nonsignificant overall effects, while Rahim et al. (2023) [360], in an umbrella review and meta-analysis, suggested that probiotics, prebiotics, and synbiotics may improve selected gastrointestinal and behavioral outcomes in ASD. Lewandowska-Pietruszka et al. (2023) [361] further confirmed consistent alterations in gut microbiota composition in ASD and highlighted Lactobacillus, Bifidobacterium, and Streptococcus species as potential therapeutic targets.

Soleimanpour et al. (2024) [362] reported a pooled improvement in behavioral symptom measures associated with probiotic supplementation. However, substantial heterogeneity in probiotic strains, treatment protocols, and outcome measures limits the strength and generalizability of this finding, while Zeng et al. (2024) [363] concluded that probiotics may exert modest beneficial effects on both gastrointestinal and behavioral symptoms. More recent meta-analyses have similarly reported small and inconsistent effects. Rojo-Marticella et al. (2025) [364] further investigated dopamine- and GABA-modulating probiotic strains, including *Lactiplantibacillus plantarum* and *Levilactobacillus brevis*, reporting improvements in ASD and ADHD symptom domains after 12 weeks of supplementation. Huang et al. (2025) [365] found a small improvement in total ASD symptom scores across eight randomized placebo-controlled trials, but rated the certainty of evidence as low, while Tang et al. (2025) [366] reported a small improvement in behavioral symptom severity without a significant pooled effect on gastrointestinal symptoms. Nonetheless, recent reviews by Barba-Vila et al. (2025) [367], Sun et al. (2025) [368], Gao et al. (2025) [369] and Chan et al. (2026) [370] suggest that current evidence remains limited by marked heterogeneity in microbiota composition, dietary influences, probiotic formulations, and study design. Particularly, Chan et al. (2026) [370], in the most recent systematic review of 14 clinical studies involving 924 children and adolescents with ASD, found that results across core ASD domains remained inconsistent. Although Lactobacillus reuteri was associated with possible improvements in social and communication functioning in some studies, the authors emphasized the limited amount of high-quality evidence and the need for strain-specific, adequately powered trials. Overall, probiotics, prebiotics, synbiotics, and microbiota-targeted therapies appear to provide modest but biologically plausible benefits in neurodevelopmental disorders associated with intellectual disability, particularly in gastrointestinal regulation, neuroinflammation, behavioral modulation, and gut–brain axis signaling, although larger and better-standardized randomized controlled trials remain necessary before definitive clinical recommendations can be established [346,347,348,349,350,351,352,353,354,355,356,357,358,359,360,361,362,363,364,365,366,367,368,369,370,371,372,373,374,375,376,377,378,379,380,381,382,383,384,385,386,387,388,389,390,391,392,393,394,395,396,397,398,399,400].

#### 3.3.3. Vitamins (D, B6, B12, Folates)

**Figure 12:**
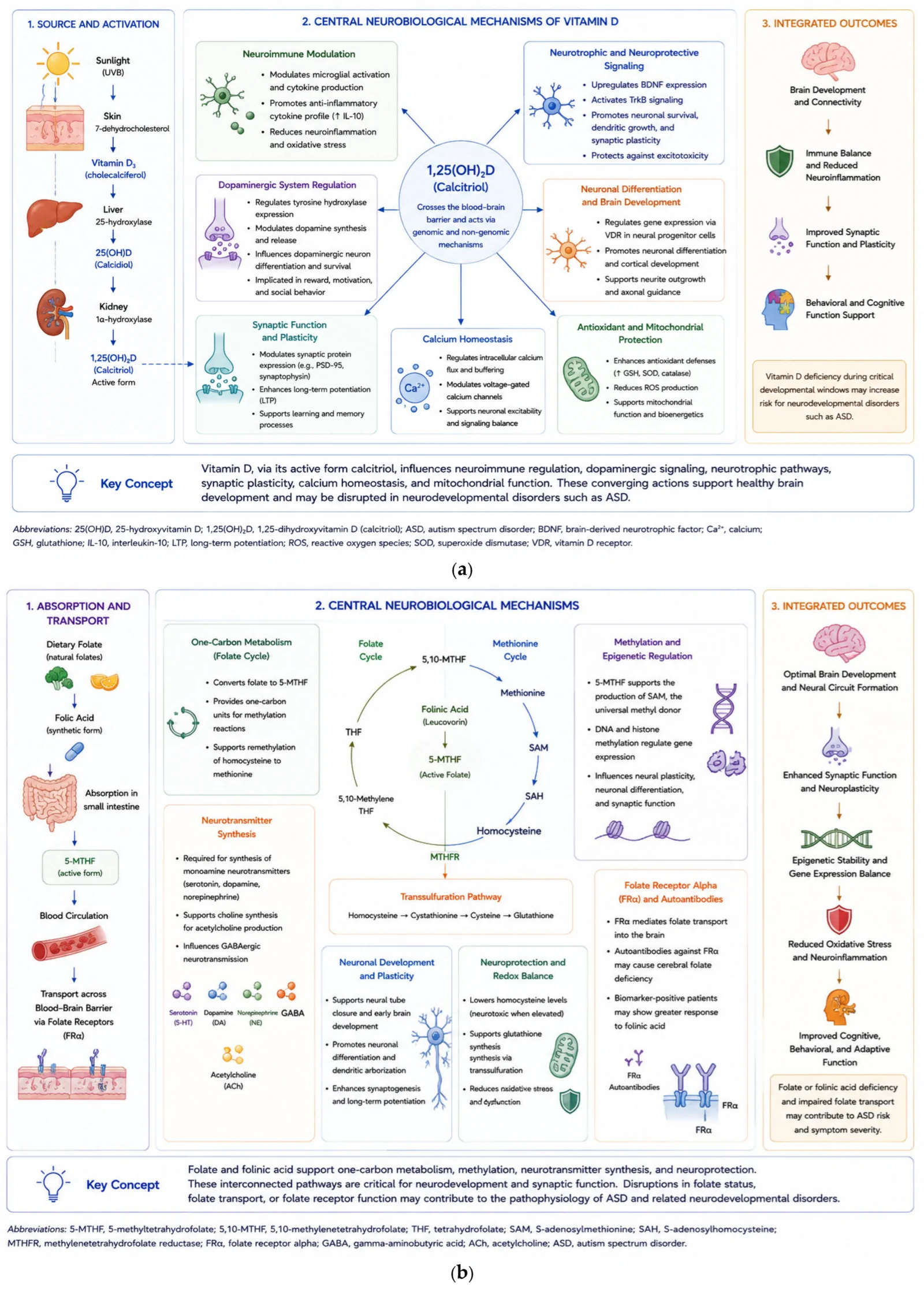

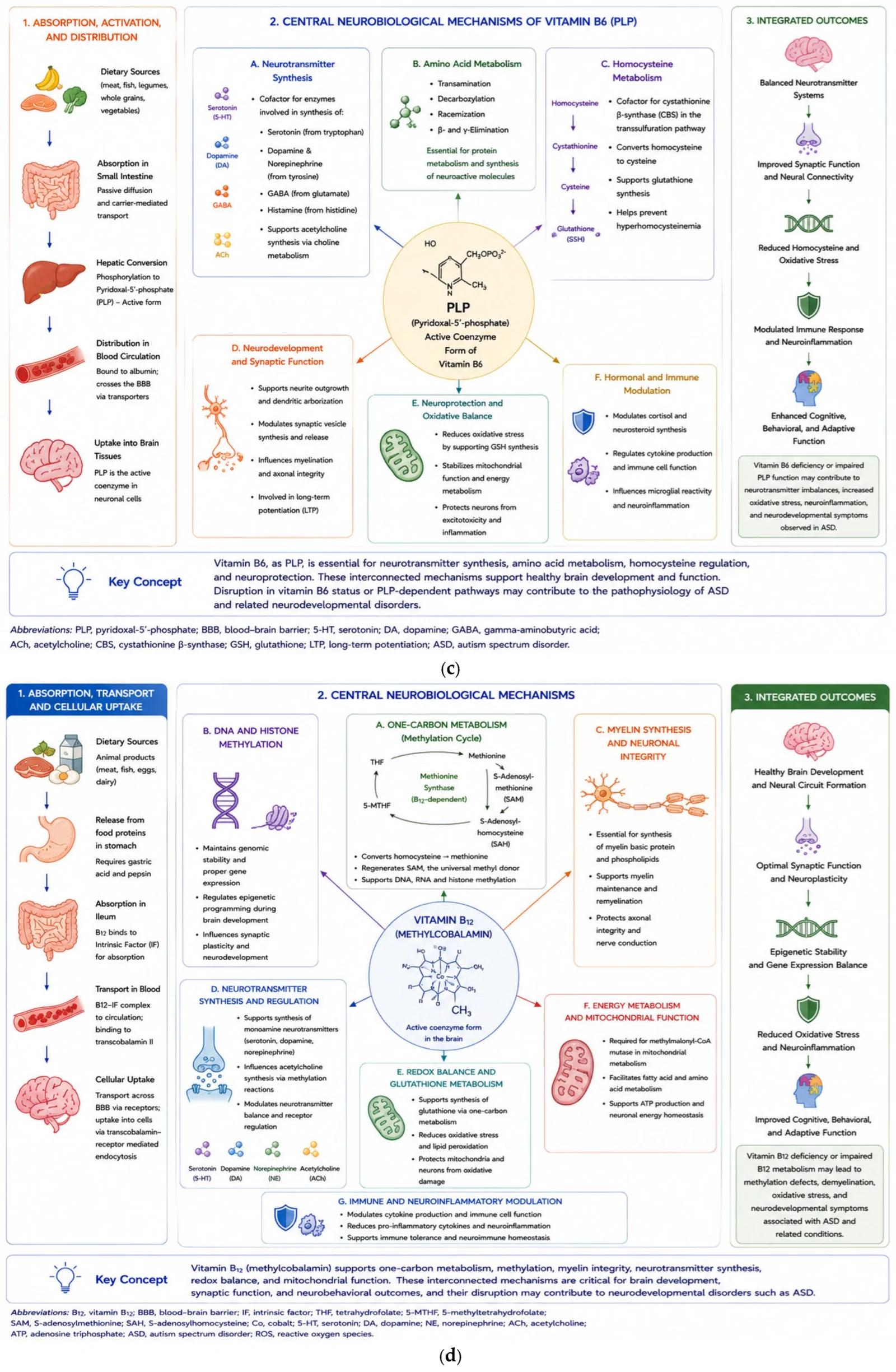

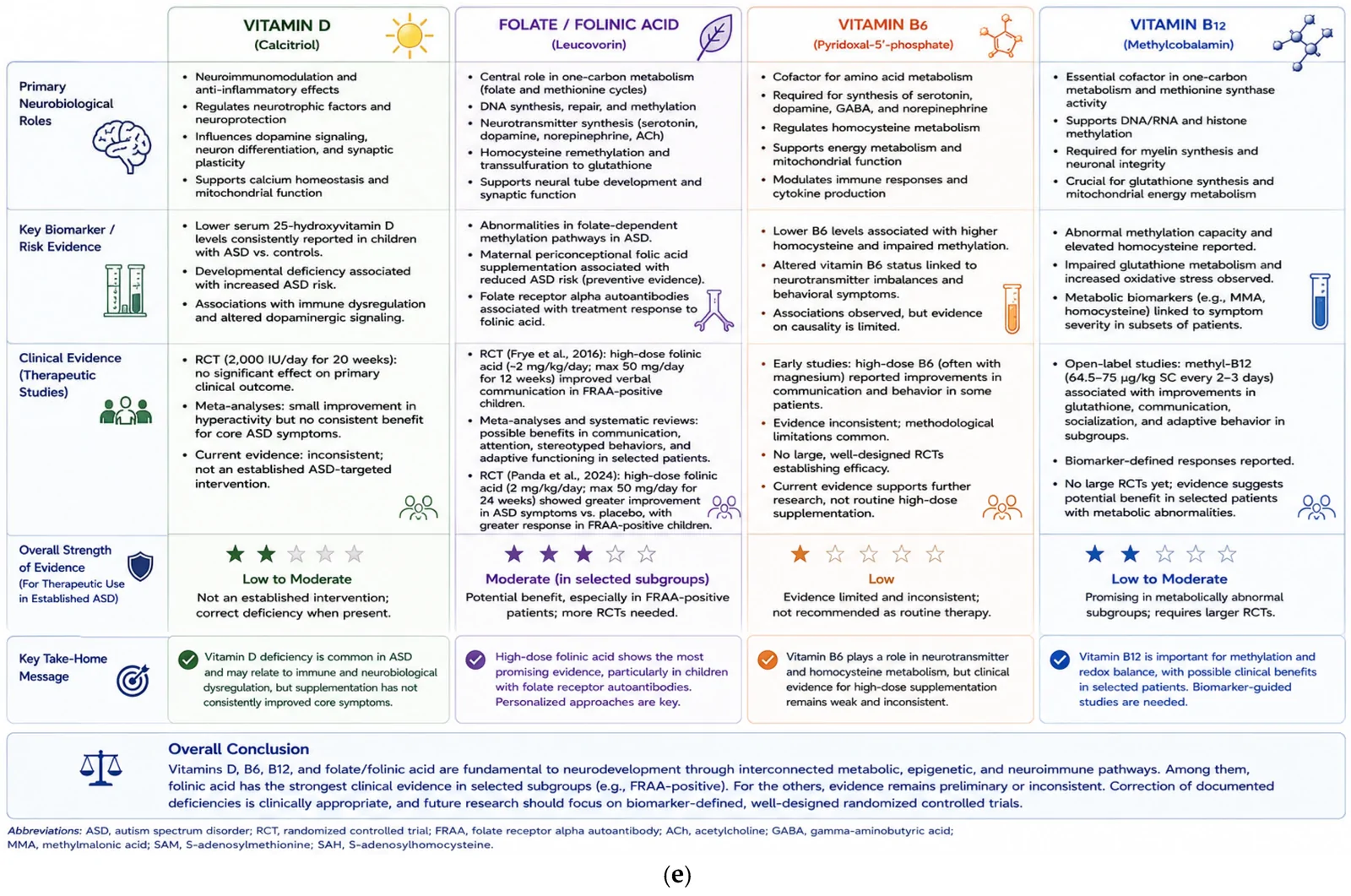
Neurobiological mechanisms potentially modulated by vitamins [403,404,405,406,407,408,409,410,411,412,413,414,415,416,417,418,419,420,421,422,423,424,425,426,427,428,429,430,431,432,433,434,435,436,437,438,439,440,441,442,443,444,445]. (**a**) Neurobiological mechanisms potentially modulated by vitamin D. (**b**) Neurobiological mechanisms potentially modulated by folate and folinic acid. (**c**) Neurobiological mechanisms potentially modulated by vitamin B6. (**d**) Neurobiological mechanisms potentially modulated by vitamin B12. (**e**) Clinical evidence for vitamins D, folinic acid/folate, B6, and B12 in neurodevelopmental disorders.

Vitamins, particularly vitamin *D*, folate/folinic acid, vitamin B6, and vitamin B12, have been extensively investigated in neurodevelopmental disorders associated with intellectual disability, especially autism spectrum disorder (ASD), because of their central role in methylation pathways, neurotransmitter synthesis, mitochondrial metabolism, immune regulation, and neurodevelopment. These neurobiological mechanisms and the available clinical evidence are summarized in Figure 12a–e [401,402,403,404,405,406,407,408,409,410,411,412,413,414,415,416,417,418,419,420,421,422,423,424,425,426,427,428,429,430,431,432,433,434,435,436,437,438,439,440,441,442,443,444,445]. Specifically, Figure 12a illustrates the neurobiological mechanisms potentially modulated by vitamin D, while Figure 12b highlights those associated with folate and folinic acid. Figure 12c presents the principal neurobiological pathways potentially influenced by vitamin B6, whereas Figure 12d summarizes the mechanisms associated with vitamin B12. Finally, Figure 12e provides an overview of the current clinical evidence regarding the potential therapeutic applications of vitamin D, folate/folinic acid, vitamin B6, and vitamin B12 in neurodevelopmental disorders. Increasing evidence suggests that abnormalities in one-carbon metabolism—including the folate cycle, methionine cycle, and transsulfuration pathway—may contribute to oxidative stress, impaired glutathione synthesis, epigenetic dysregulation, and neuroinflammation in ASD and related neurodevelopmental disorders (Table 25 and Table 26). These metabolic abnormalities may affect synaptic plasticity, neuronal connectivity, mitochondrial function, and neurotransmitter balance during critical periods of brain development.

Vitamin D has received considerable attention because of its neuroimmunomodulatory and neuroprotective properties. Developmental vitamin D deficiency has been associated with altered dopaminergic signaling, immune dysregulation, abnormal neuronal differentiation, and increased ASD risk (Lee et al., 2019; Principi et al., 2020) [402,403]. Several observational studies demonstrated significantly lower serum 25-hydroxyvitamin D concentrations in children with ASD compared with neurotypical controls (Petruzzelli et al., 2020 [404]; Altun et al., 2018 [401], although such associations do not establish causality or therapeutic efficacy. Randomized trials of vitamin D supplementation have produced inconsistent results. Kerley et al. (2017) [405], using 2000 IU/day for 20 weeks, found no significant effect on the primary clinical outcome, whereas subsequent meta-analytic evidence suggested a small improvement in hyperactivity but no consistent benefit for core ASD symptoms. More recent reviews similarly conclude that vitamin *D* supplementation cannot currently be considered an established ASD-targeted intervention, although correction of documented deficiency remains clinically appropriate and specific subgroups may warrant further investigation. 

Folate metabolism abnormalities have also been investigated in ASD and related neurodevelopmental conditions. Schmidt et al. (2012) [406] demonstrated that maternal periconceptional folic acid supplementation was associated with reduced ASD risk in offspring, although these data are preventive and do not establish treatment efficacy in children with established ASD. Folate and folinic acid play important roles in one-carbon metabolism, DNA methylation, neurotransmitter synthesis, and neural tube development, and several studies reported abnormalities in folate-dependent methylation pathways in ASD (Roufael et al., 2023) [407]. Frye et al. (2016) [408] reported that high-dose folinic acid treatment improved verbal communication in children with ASD and language impairment, particularly in those positive for folate receptor alpha autoantibodies. Subsequently, Rossignol and Frye (2021) [409], in a systematic review and meta-analysis, concluded that *leucovorin (folinic acid)* supplementation may improve communication, attention, stereotyped behaviors, and adaptive functioning in selected ASD populations. A commonly studied high-dose folinic acid regimen is approximately 2 mg/kg/day (maximum 50 mg/day). Subsequent reviews have supported further investigation of leucovorin in biomarker-defined subgroups, while emphasizing heterogeneity and the limited number of adequately powered trials. 

Vitamin *B12*, particularly methylcobalamin (methyl-B12), has also attracted growing attention because of its role in methylation and glutathione metabolism. Several studies have reported abnormalities in methylation capacity and glutathione metabolism in children with ASD, suggesting impaired detoxification and increased oxidative stress. James et al. (2009) [410] reported that *methyl-B12* supplementation improved glutathione redox status and methylation metabolism in children with ASD. Subsequent clinical studies suggested possible improvements in communication, socialization, and adaptive behavior following *methyl-B12* therapy, particularly in metabolically abnormal subgroups (Frye et al., 2013 [411]; Hendren et al., 2016 [412]). Typical regimens involve subcutaneous methyl-B12 administration at doses of approximately 64.5–75 µg/kg every 2–3 days. Recent reviews by Zwierz et al. (2025) [413] further highlighted the importance of vitamin *B12* in one-carbon metabolism and oxidative stress regulation in ASD.

Vitamin B6 has historically been investigated in ASD because of its role as a cofactor in the synthesis of serotonin, dopamine, gamma-aminobutyric acid (GABA), and other neurotransmitters. Earlier studies suggested that high-dose vitamin B6 combined with magnesium may improve communication, behavioral symptoms, and social interaction in some ASD patients; however, findings have remained inconsistent and often limited by methodological weaknesses (Jennings et al., 2022) [414]. Low vitamin B6 levels have additionally been associated with elevated homocysteine concentrations and impaired methylation pathways in ASD populations (Altun et al., 2018) [401]. Available evidence remains insufficient to support routine vitamin B6 supplementation for ASD. Reported vitamin B6 doses vary substantially across studies, generally ranging from 30 to 300 mg/day.

Emerging evidence also suggests potential roles for vitamin-based interventions in ADHD, Down syndrome, Fragile X syndrome, epilepsy-associated intellectual disability, and other neurodevelopmental disorders, although available data remain more limited compared with ASD. Importantly, several studies emphasized that treatment response may depend on baseline nutritional deficiencies, metabolic phenotypes, genetic factors, and the presence of biomarkers such as folate receptor alpha autoantibodies. Moreover, considerable heterogeneity persists across clinical trials regarding patient stratification, dosing regimens, treatment duration, and outcome measures.

Overall, vitamins involved in one-carbon metabolism, redox regulation, immune signaling, and neurodevelopment provide a plausible biological framework for targeted nutritional interventions in selected neurodevelopmental populations. Among the approaches reviewed, folinic acid currently has the clearest direct clinical signal in selected children with ASD, particularly those with language impairment or folate receptor alpha autoantibodies, whereas methyl-B12 shows preliminary evidence mainly in metabolically defined subgroups. Evidence for vitamin D supplementation as a general ASD-targeted intervention remains inconsistent, despite frequent observational associations with low vitamin D status, and evidence for vitamin B6 remains limited and methodologically weak. Future trials should prioritize biomarker-defined populations, standardized dosing, adequately powered randomized designs, and long-term safety and functional outcomes [401,402,403,404,405,406,407,408,409,410,411,412,413,414,415,416,417,418,419,420,421,422,423,424,425,426,427,428,429,430,431,432,433,434,435,436,437,438,439,440,441,442,443,444,445].

#### 3.3.4. Magnesium

**Figure 13:**
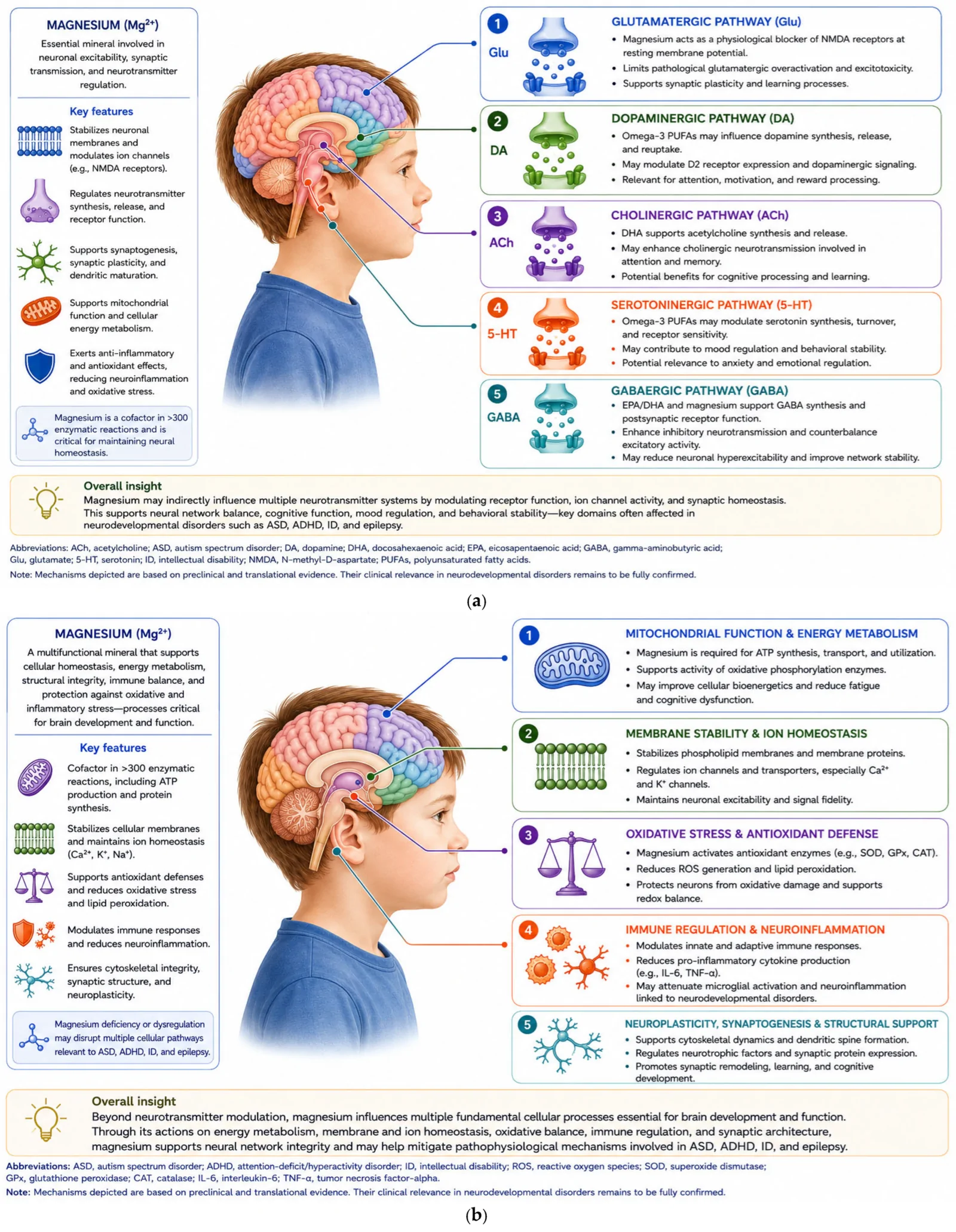

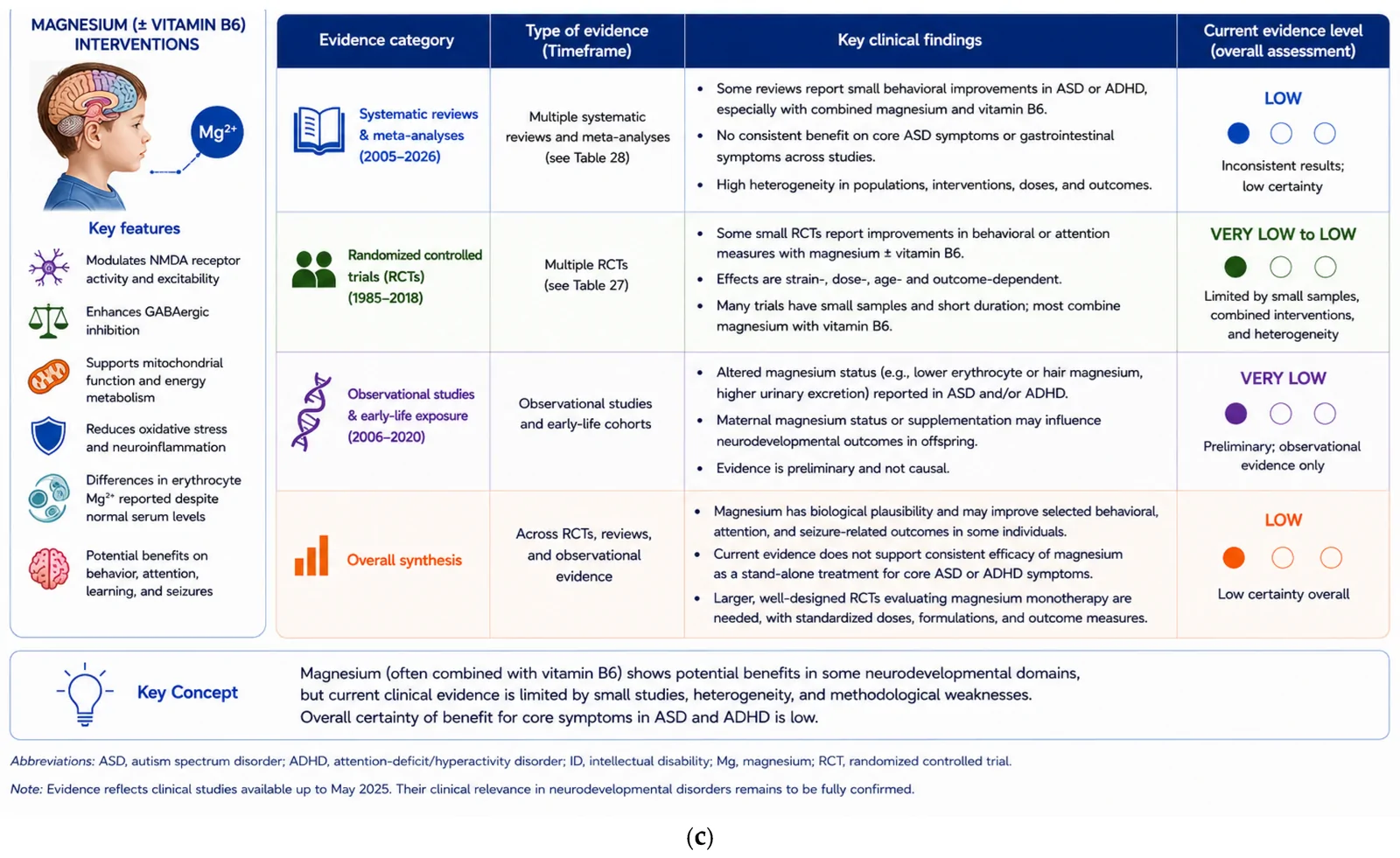
Neurobiological mechanisms and clinical evidence of Magnesium [446,447,448,449,450,451,452,453,454,455,456,457,458,459,460,461,462,463,464,465,466,467,468,469,470,471,472,473,474,475,476,477,478,479]. (**a**) Major neurotransmitter pathways potentially modulated by Magnesium. (**b**) Principal non-neurotransmitter mechanisms, including glutathione/redox regulation, anti-inflammatory and immune modulation, mitochondrial protection, and cellular homeostasis. (**c**) Summary of current clinical evidence in neurodevelopmental conditions.

Magnesium has been increasingly investigated as a potential adjunctive treatment in neurodevelopmental disorders associated with intellectual disability, particularly autism spectrum disorder (ASD), attention-deficit/hyperactivity disorder (ADHD), and epilepsy-associated neurodevelopmental conditions, because of its central role in neuronal excitability, synaptic transmission, mitochondrial metabolism, membrane stabilization, and neurotransmitter regulation. These neurobiological mechanisms and the available clinical evidence are summarized in Figure 13a–c [446,447,448,449,450,451,452,453,454,455,456,457,458,459,460,461,462,463,464,465,466,467,468,469,470,471,472,473,474,475,476,477,478,479]. Specifically, Figure 13a illustrates the major neurotransmitter pathways potentially modulated by magnesium, while Figure 13b highlights the principal non-neurotransmitter mechanisms, including glutathione and redox regulation, anti-inflammatory and immune modulation, mitochondrial protection, and cellular homeostasis. Figure 13c provides an overview of the current clinical evidence regarding the potential therapeutic applications of magnesium in neurodevelopmental conditions. Magnesium is an essential cofactor in more than 300 enzymatic reactions and contributes to glutamatergic, GABAergic, dopaminergic, and serotonergic signaling pathways. One of the most relevant neurobiological mechanisms involves modulation of N-methyl-D-aspartate (NMDA) receptors, where magnesium acts as a physiological antagonist, reducing glutamate-mediated excitotoxicity and neuronal hyperexcitability. Magnesium additionally enhances GABAergic inhibition, stabilizes neuronal membranes, supports mitochondrial ATP production, and reduces oxidative stress and neuroinflammation, all mechanisms implicated in ASD, ADHD, epilepsy, and intellectual disability (Table 27 and Table 28).

Early investigations primarily focused on combined magnesium and vitamin B6 supplementation in ASD. Martineau et al. (1985) [447], in one of the earliest double-blind placebo-controlled studies, reported behavioral improvements following vitamin B6 and magnesium supplementation in autistic children, including reductions in social withdrawal and stereotyped behaviors together with normalization of electrophysiological and biochemical markers such as urinary homovanillic acid excretion. However, the complex historical design and limited replication restrict interpretation according to contemporary trial standards. Martineau et al. (1988) [448] subsequently reported clinical, biochemical, and electrophysiological changes during an 8-week open study in 11 children specifically selected for previous responsiveness to B6–Mg; this enrichment strategy substantially limits generalizability and does not establish the existence of a reproducible responder phenotype. Mousain-Bosc et al. (2006b) [449] reported beneficial effects in ADHD populations, with improvements in attention, emotional regulation, and school performance. However, the uncontrolled design, small sample, and combined intervention prevent causal attribution of the observed changes specifically to magnesium. These studies also reported differences in erythrocyte magnesium concentrations despite frequently normal serum magnesium levels, raising the hypothesis that conventional serum measurements may not fully reflect magnesium status in neurodevelopmental populations. Subsequent investigations further explored *magnesium* dysregulation in ASD and ADHD. Skalny et al. (2020) [446] analyzed serum, hair, and urinary magnesium concentrations in children with ASD and ADHD and reported differences in magnesium-related biomarker profiles across diagnostic groups. These findings suggest possible alterations in magnesium status or distribution; however, peripheral biomarkers cannot establish intracellular or central nervous system magnesium deficiency, causality, or a relationship with therapeutic response. 

Magnesium also has well-established roles in neuronal excitability and NMDA-receptor function, providing a mechanistic rationale for interest in neurological conditions characterized by hyperexcitability or seizures. However, direct clinical evidence supporting magnesium supplementation for epilepsy-associated intellectual disability is currently insufficient. Experimental studies demonstrated that magnesium deficiency increases seizure susceptibility through excessive NMDA receptor activation and glutamatergic hyperexcitability, whereas magnesium supplementation may reduce neuronal excitability and oxidative stress. These mechanisms may be particularly relevant in neurodevelopmental disorders characterized by epilepsy, behavioral dysregulation, and excitatory/inhibitory imbalance.

Several formulations of *magnesium* have been investigated, including magnesium citrate, magnesium glycinate, magnesium oxide, and magnesium L-threonate, which differ in gastrointestinal tolerability, blood–brain barrier penetration, and bioavailability. Magnesium L-threonate has recently attracted interest because of its enhanced central nervous system penetration and potential effects on synaptic plasticity and cognition, although evidence in pediatric neurodevelopmental disorders remains limited. Typical supplementation regimens in ASD and ADHD studies generally range from approximately 6 to 10 mg/kg/day, frequently combined with vitamin B6.

Despite promising biological rationale and some positive clinical findings, systematic reviews and meta-analyses have produced more cautious conclusions. Nye and Brice (2005) [450], in a Cochrane systematic review, concluded that evidence supporting vitamin B6–magnesium therapy in ASD remained insufficient because of small sample sizes, inadequate controls, and methodological limitations. Murza et al. (2010) [451] similarly emphasized that available evidence remained inconclusive despite some reported behavioral benefits. More recent integrative nutritional studies, including Adams et al. (2018) [193], reported improvements in several clinical and metabolic outcomes following comprehensive multicomponent nutritional interventions; however, these findings cannot establish an independent therapeutic contribution of magnesium.

Magnesium supplementation is generally well tolerated, although gastrointestinal adverse effects such as diarrhea and abdominal discomfort may occur, particularly with poorly absorbed formulations. Hypermagnesemia is rare in children with normal renal function but may occur in the presence of renal insufficiency or excessive dosing. Overall, magnesium has a strong biological rationale in neurodevelopment because of its roles in NMDA receptor regulation, neuronal excitability, energy metabolism, and excitatory–inhibitory balance. However, clinical evidence supporting magnesium supplementation as a treatment for ASD or ADHD remains limited and of low certainty. Most positive findings derive from small, older, uncontrolled, or combined magnesium–vitamin B6 studies, making the independent contribution of magnesium difficult to determine. More recent systematic reviews continue to identify substantial methodological heterogeneity and insufficient high-quality randomized evidence. Future studies should evaluate magnesium monotherapy and clearly defined formulations in adequately powered, biomarker-stratified randomized controlled trials [446,447,448,449,450,451,452,453,454,455,456,457,458,459,460,461,462,463,464,465,466,467,468,469,470,471,472,473,474,475,476,477,478,479].

### 3.4. Other Phytotherapeutics

Several additional phytotherapeutic compounds have been investigated for their potential role in neurodevelopmental and cognitive disorders. 

**Figure 14:**
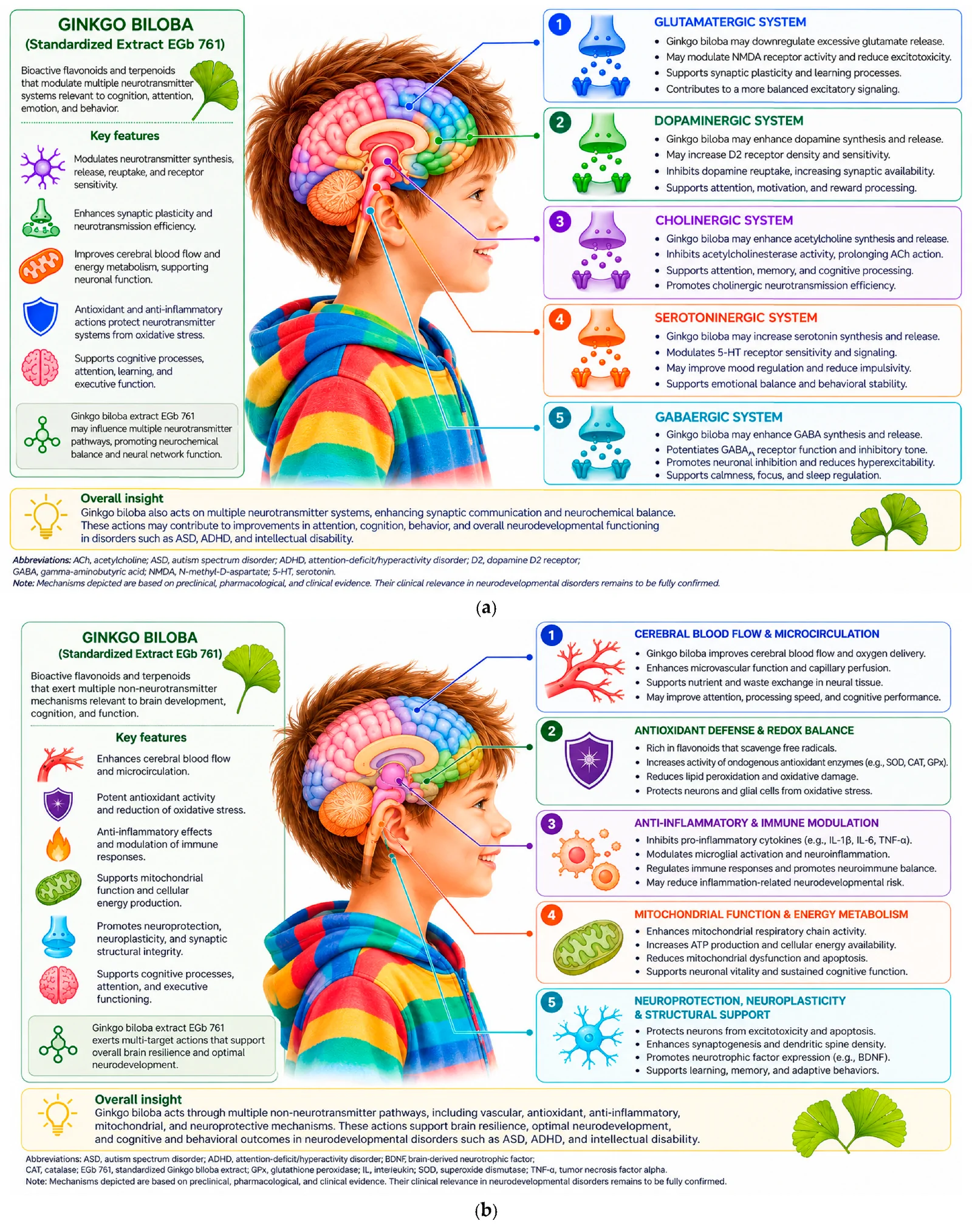

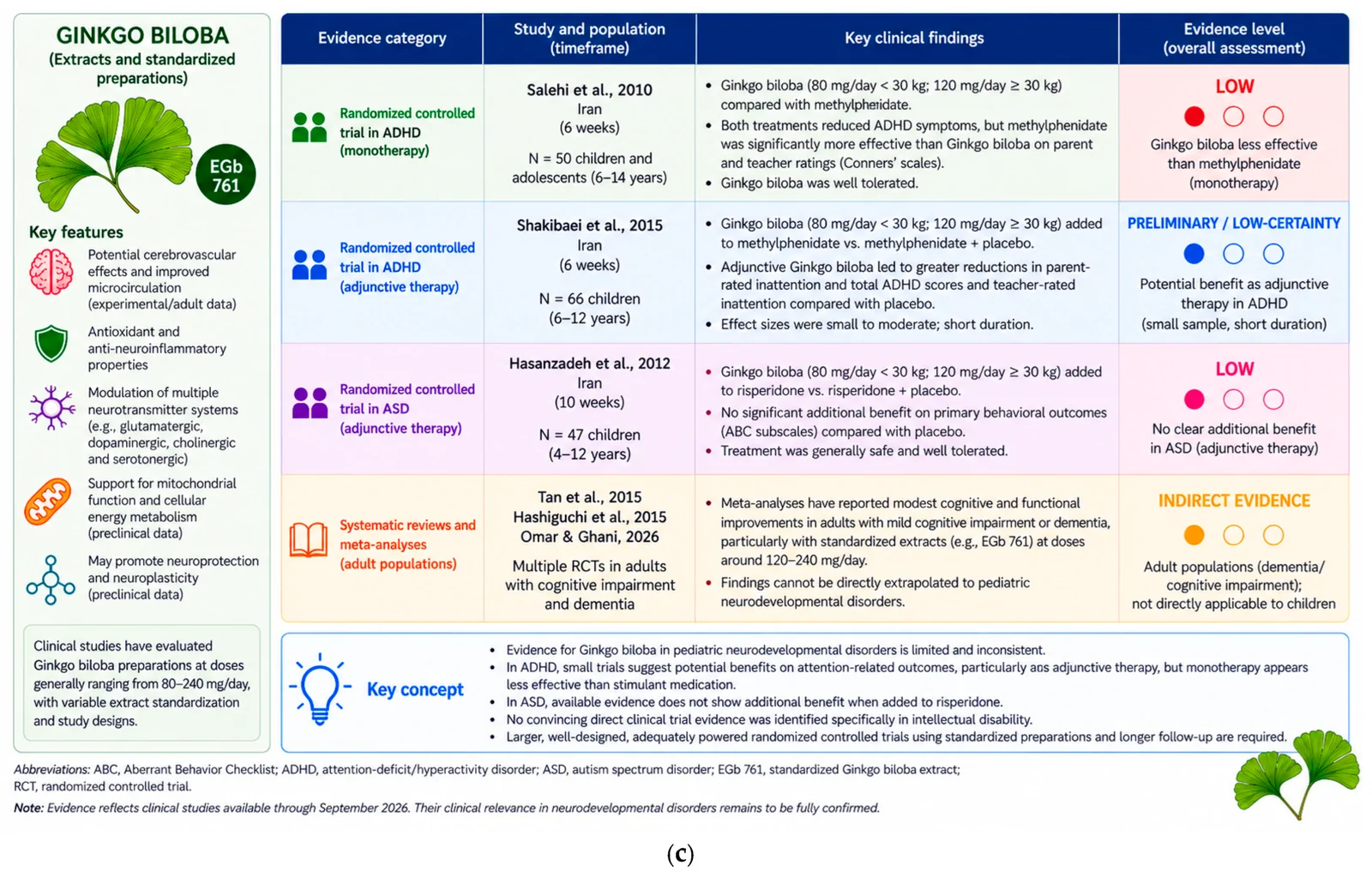
Neurobiological mechanisms and clinical evidence of *Ginkgo biloba* [480,481,482,483,484,485,486,487,488,489,490,491,492,493,494,495,496,497,498]. (**a**) Major neurotransmitter pathways potentially modulated by *Ginkgo biloba*. (**b**) Principal non-neurotransmitter mechanisms, including glutathione/redox regulation, anti-inflammatory and immune modulation, mitochondrial protection, and cellular homeostasis. (**c**) Summary of current clinical evidence in neurodevelopmental conditions.

*Ginkgo biloba* has been investigated for its antioxidant, neuroprotective, vascular, and neurotransmitter-modulating properties. These neurobiological mechanisms and the available clinical evidence are summarized in Figure 14a–c [480,481,482,483,484,485,486,487,488,489,490,491,492,493,494,495,496,497,498]. Specifically, Figure 14a illustrates the major neurotransmitter pathways potentially modulated by *Ginkgo biloba*, while Figure 14b highlights the principal non-neurotransmitter mechanisms, including glutathione and redox regulation, anti-inflammatory and immune modulation, mitochondrial protection, and cellular homeostasis. Figure 14c provides an overview of the current clinical evidence regarding the potential therapeutic applications of *Ginkgo biloba* in neurodevelopmental conditions. Standardized *Ginkgo biloba* extracts, particularly EGb 761, have been extensively studied in adult cognitive impairment and dementia, where systematic reviews and meta-analyses have reported potential benefits on cognition and activities of daily living, particularly at doses around 240 mg/day. However, these findings derive predominantly from older adult populations and cannot be directly extrapolated to pediatric neurodevelopmental disorders (Hashiguchi et al., 2015; Tan et al., 2015) [480,481]. Direct clinical evidence in neurodevelopmental disorders is considerably more limited and has focused mainly on attention-deficit/hyperactivity disorder (ADHD). Salehi et al. [482] evaluated *Ginkgo biloba* (80–120 mg/day for 6 weeks) against methylphenidate in 50 children with ADHD and found *Ginkgo biloba* to be less effective than methylphenidate in reducing parent- and teacher-rated ADHD symptoms. Conversely, Shakibaei et al. (2015) [483], in a randomized double-blind placebo-controlled trial, evaluated *Ginkgo biloba* (80–120 mg/day) as adjunctive treatment to methylphenidate in children with ADHD. Sixty-six participants were randomized, and adjunctive Ginkgo biloba was associated with greater reductions in parent-rated inattention and total ADHD scores and teacher-rated inattention compared with methylphenidate plus placebo, although the short treatment duration and adjunctive design prevent conclusions regarding *Ginkgo biloba* monotherapy. Evidence in autism spectrum disorder (ASD) is even more limited. Hasanzadeh et al. (2012) [484] evaluated *Ginkgo biloba* as adjunctive treatment to risperidone in a randomized double-blind placebo-controlled trial in children with autistic disorder. The addition of *Ginkgo biloba* did not produce a significant advantage over risperidone plus placebo on the primary behavioral outcomes, although treatment was generally tolerated. Overall, *Ginkgo biloba* has biological plausibility and preliminary clinical evidence for selected attention-related outcomes in pediatric ADHD, particularly as adjunctive treatment, but available findings are inconsistent and based on a small number of short-duration trials. Evidence supporting efficacy in ASD is currently insufficient, and no convincing direct clinical evidence supports its use specifically in intellectual disability. Consequently, larger adequately powered randomized controlled trials using standardized extracts, clearly defined neurodevelopmental populations, and longer follow-up are required before therapeutic efficacy can be established [480,481,482,483,484,485,486,487,488,489,490,491,492,493,494,495,496,497,498].

**Figure 15:**
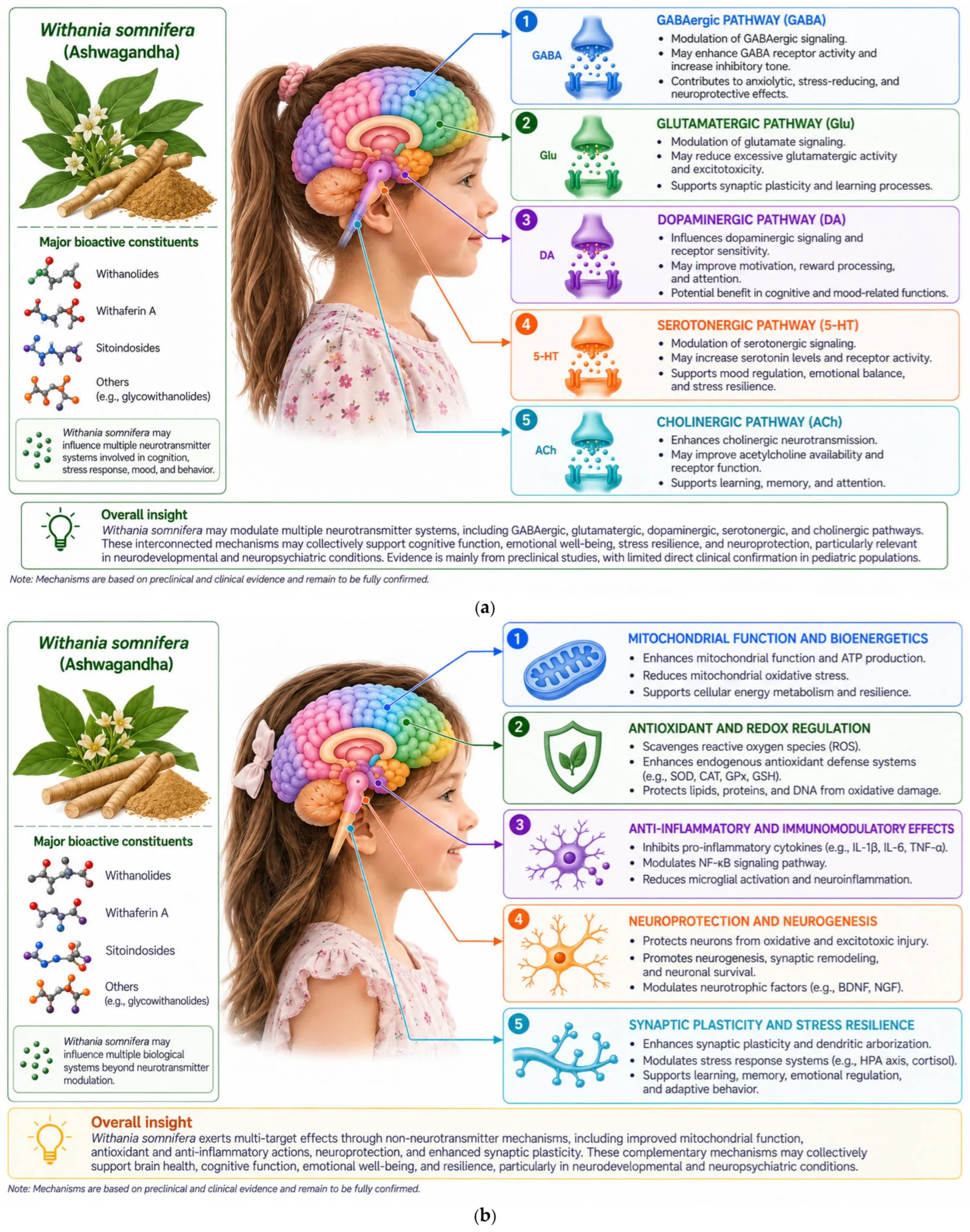

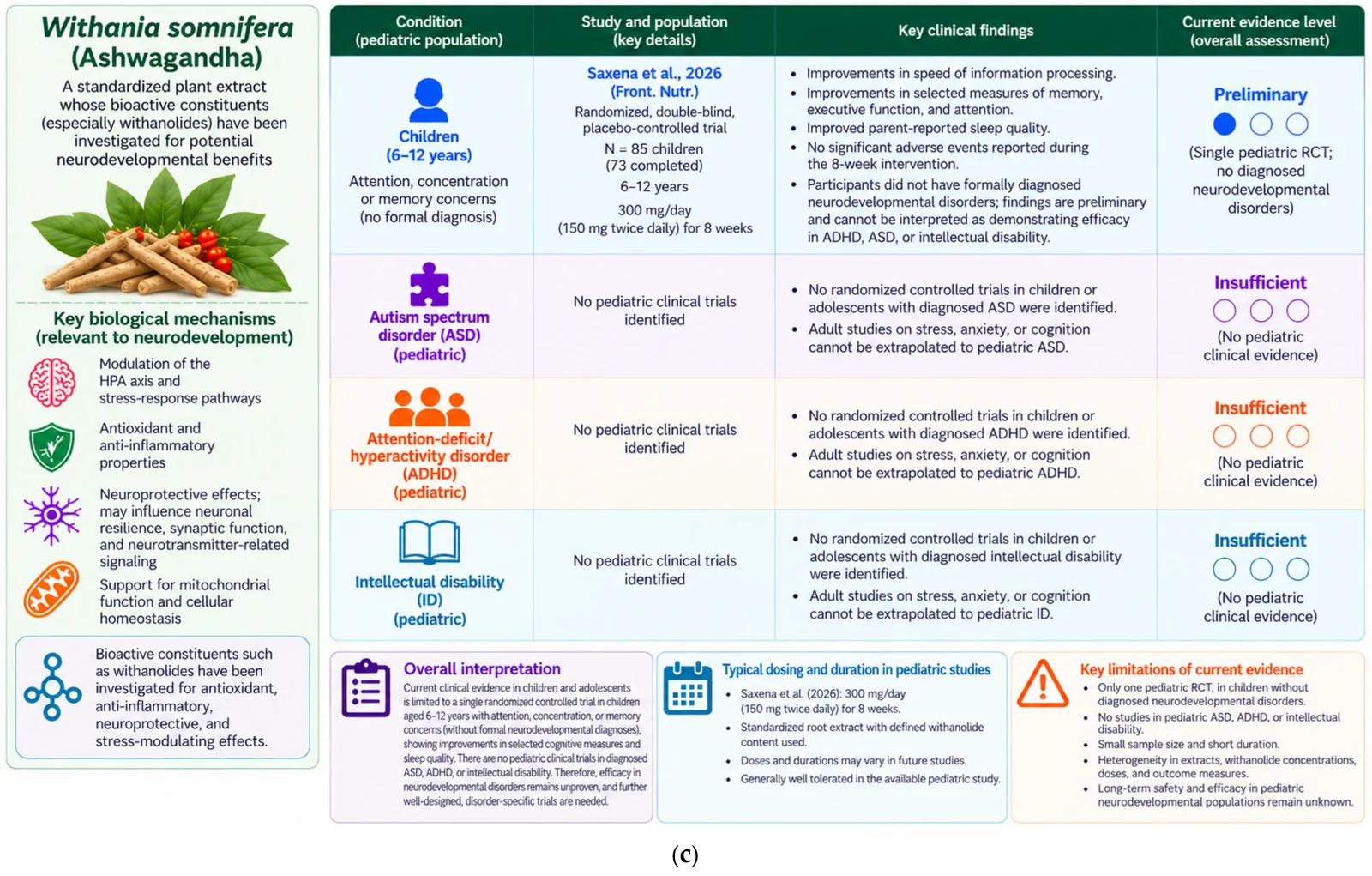
Neurobiological mechanisms and clinical evidence of *Withania somnifera* (*Ashwagandha*) [499,500,501,502,503,504,505,506,507,508,509,510,511,512]. (**a**) Major neurotransmitter pathways potentially modulated by *Withania somnifera* (*Ashwagandha*). (**b**) Principal non-neurotransmitter mechanisms, including glutathione/redox regulation, anti-inflammatory and immune modulation, mitochondrial protection, and cellular homeostasis. (**c**) Summary of current clinical evidence in neurodevelopmental conditions.

*Withania somnifera* (Ashwagandha) is an adaptogenic medicinal plant whose bioactive constituents, particularly withanolides, have been investigated for antioxidant, anti-inflammatory, neuroprotective, and stress-modulating properties. These neurobiological mechanisms and the available clinical evidence are summarized in Figure 15a–c [499,500,501,502,503,504,505,506,507,508,509,510,511,512,513]. Specifically, Figure 15a illustrates the major neurotransmitter pathways potentially modulated by *Withania somnifera* (Ashwagandha), while Figure 15b highlights the principal non-neurotransmitter mechanisms, including glutathione and redox regulation, anti-inflammatory and immune modulation, mitochondrial protection, and cellular homeostasis. Figure 15c provides an overview of the current clinical evidence regarding the potential therapeutic applications of *Withania somnifera* (Ashwagandha) in neurodevelopmental conditions.Experimental evidence suggests that W. somnifera may influence hypothalamic–pituitary–adrenal (HPA) axis activity, oxidative and inflammatory pathways, neuronal resilience, and neurotransmitter-related signaling. Human studies, conducted predominantly in adults rather than neurodevelopmental populations, suggest potential effects on perceived stress, anxiety, cortisol concentrations, sleep quality, and selected cognitive outcomes. Recent systematic reviews and meta-analyses have reported reductions in stress and anxiety measures and, in some studies, cortisol levels and improvements in selected sleep parameters; however, substantial heterogeneity in extracts, withanolide concentrations, doses, study populations, and outcome measures limits definitive conclusions [499,500,501,502,503,504,505,506,507,508,509,510,511,512]. More recently, Saxena et al. (2026) [503] conducted an 8-week randomized, double-blind, placebo-controlled trial evaluating standardized *W. somnifera* root extract in 85 children aged 6–12 years with parent-reported concerns regarding attention, concentration, or memory. Participants received Ashwagandha root extract (150 mg twice daily; 300 mg/day) or placebo, and 73 participants completed the study. Supplementation was associated with improvements in speed of information processing and selected measures of memory, executive function, and attention, as well as improved parent-reported sleep quality. No significant adverse events were reported during the 8-week intervention. Importantly, participants did not have formally diagnosed neurodevelopmental disorders; therefore, these findings provide preliminary pediatric evidence but cannot be interpreted as demonstrating efficacy in ADHD, ASD, or intellectual disability. Overall, although modulation of stress-response pathways, neuroinflammation, oxidative stress, and neuronal function provides a plausible mechanistic rationale for investigating Ashwagandha in neurodevelopmental conditions, direct clinical evidence in diagnosed ASD, ADHD, and ID remains insufficient. Benefits observed in adult stress and sleep studies cannot be directly extrapolated to these populations, and the recent pediatric findings require replication in disorder-specific, adequately powered randomized controlled trials [499,500,501,502,503,504,505,506,507,508,509,510,511,512,513].

**Figure 16:**
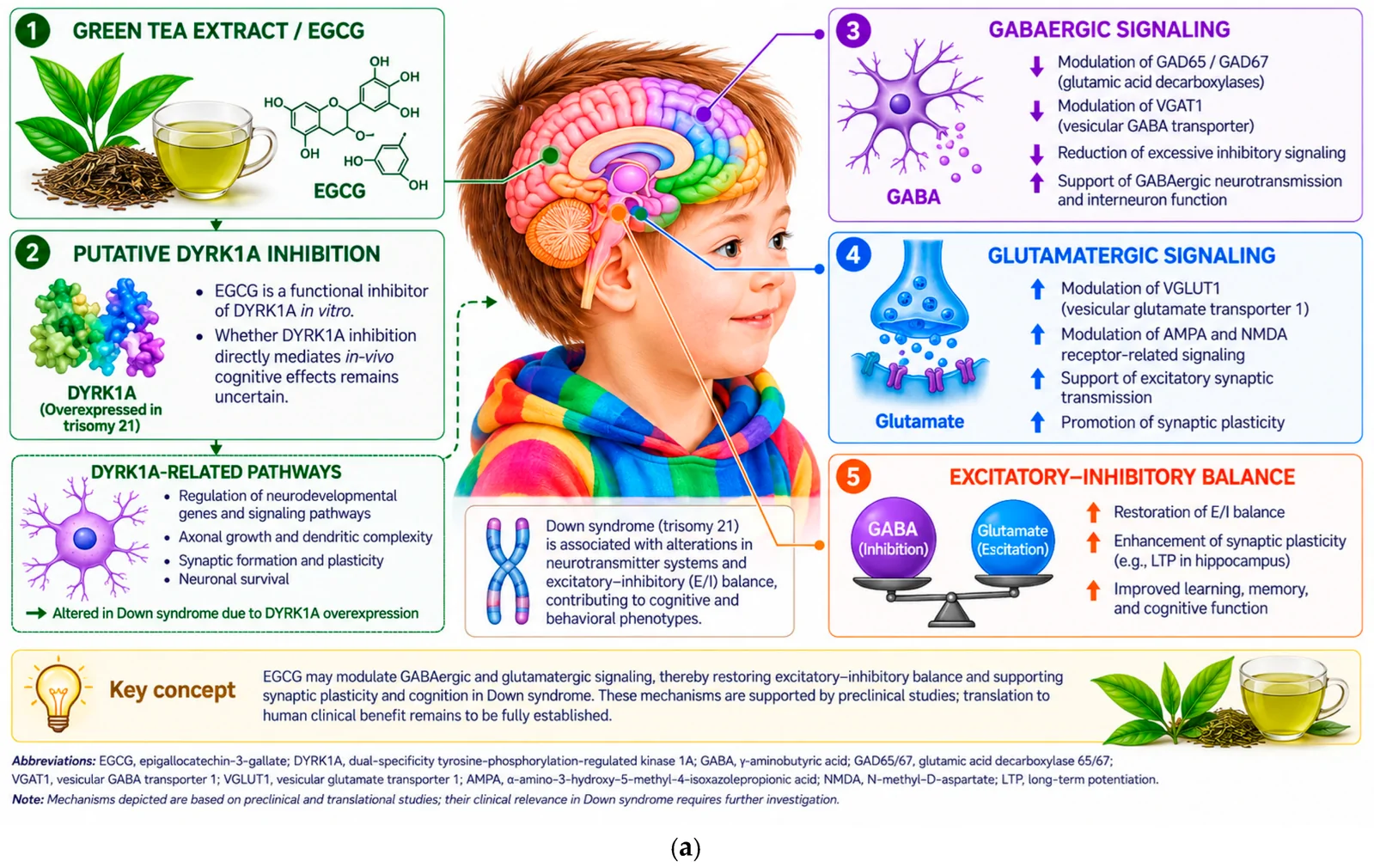

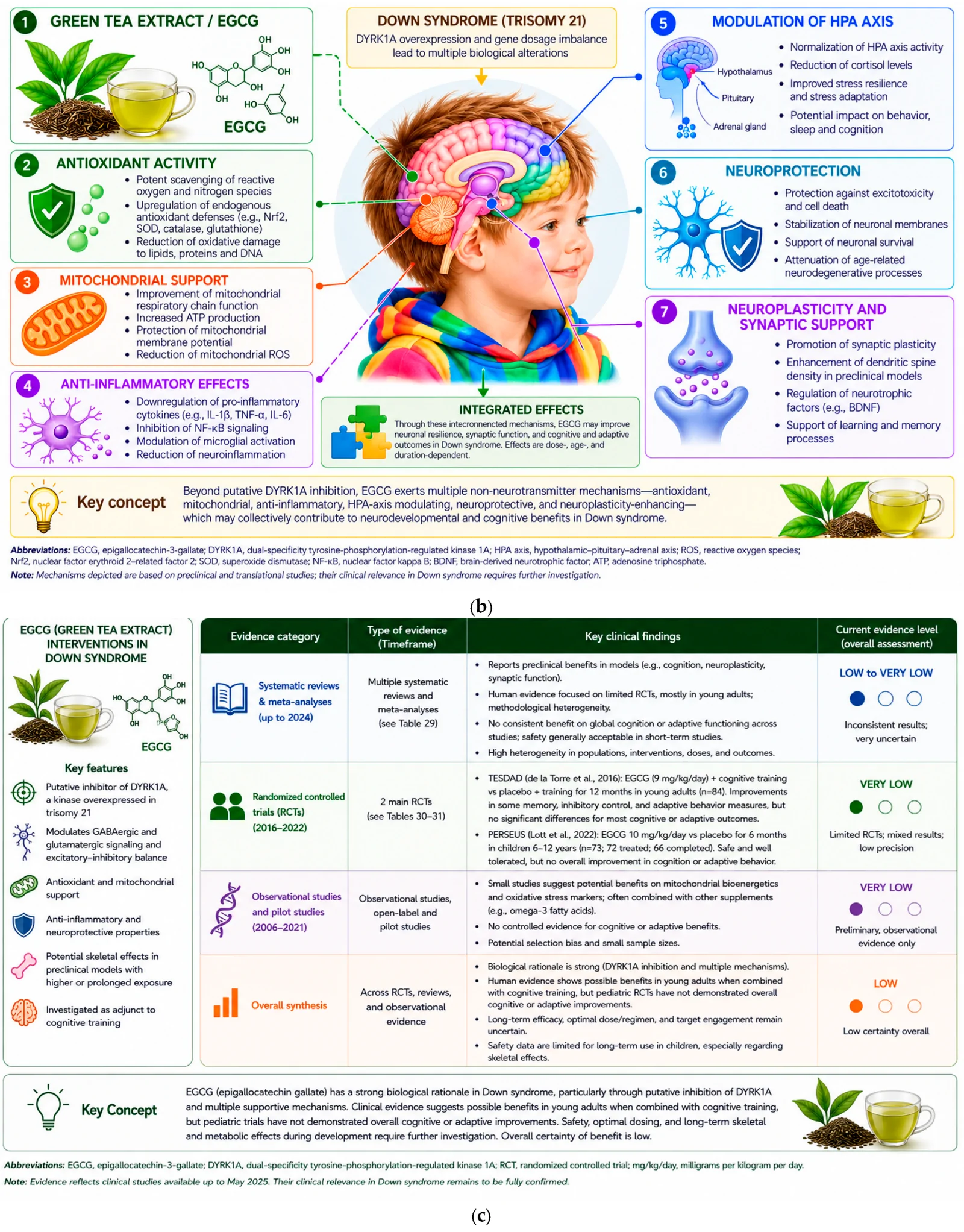
Neurobiological mechanisms and clinical evidence of Green Tea [514,515,516,517,518,519,520,521,522,523,524,525,526,527,528,529,530,531,532,533,534,535,536,537]. (**a**) Major neurotransmitter pathways potentially modulated by EGCG. (**b**) Principal non-neurotransmitter mechanisms. (**c**) Summary of current clinical evidence in neurodevelopmental conditions.

Green tea extracts, particularly those enriched in epigallocatechin-3-gallate (EGCG)*,* have attracted considerable interest in Down syndrome (DS), one of the most common genetic causes of intellectual disability, because EGCG has been investigated as a functional inhibitor of dual-specificity tyrosine-phosphorylation-regulated kinase 1A (DYRK1A), a chromosome 21-encoded kinase whose gene dosage is increased in trisomy 21. In addition to its potential effects on DYRK1A-related signaling, EGCG exhibits antioxidant, mitochondrial, anti-inflammatory, and neuroplasticity-related properties that may be relevant to the complex neurobiology of DS. These neurobiological mechanisms and the available clinical evidence are summarized in Figure 16a–c [514,515,516,517,518,519,520,521,522,523,524,525,526,527,528,529,530,531,532,533,534,535,536,537]. Specifically, Figure 16a illustrates the major neurotransmitter pathways potentially modulated by epigallocatechin-3-gallate (EGCG), while Figure 16b highlights the principal non-neurotransmitter mechanisms, including DYRK1A-related signaling, antioxidant and anti-inflammatory activity, mitochondrial regulation, and neuroplasticity-related pathways. Figure 16c provides an overview of the current clinical evidence regarding the potential therapeutic applications of green tea extracts, particularly EGCG, in neurodevelopmental conditions, with particular emphasis on Down syndrome. Preclinical studies have reported improvements in selected cognitive and neurodevelopmental phenotypes in some DS models; however, findings have been inconsistent across doses, developmental windows, formulations, and experimental models, and several studies have failed to reproduce cognitive benefits. Clinical evidence has similarly produced mixed findings. In the phase 2 TESDAD randomized controlled trial, de la Torre et al. (2016) [519] evaluated EGCG (9 mg/kg/day) combined with cognitive training for 12 months in young adults with DS. Among 84 participants included in the intention-to-treat analysis, the combined intervention produced improvements in selected measures of visual recognition memory, inhibitory control, and adaptive behavior compared with placebo plus cognitive training. However, significant between-group differences were absent for most cognitive and adaptive outcomes, and the combined design prevents attribution of the observed effects specifically to EGCG. Subsequent pediatric evidence has been less supportive. The PERSEUS randomized phase Ib trial evaluated EGCG (10 mg/kg/day) for 6 months in 73 children with DS aged 6–12 years. Seventy-two children received treatment and 66 completed the study. EGCG was generally safe and well tolerated, but did not improve overall cognitive performance or adaptive functionality compared with placebo, although exploratory secondary findings suggested that specific subgroups may warrant further investigation [520]. Additional pediatric pilot evidence has suggested potential effects of EGCG-containing interventions on mitochondrial bioenergetics, including complex I and ATP-synthase activity; however, small samples and combined formulations, including co-administration with omega-3 fatty acids, substantially limit conclusions regarding clinical efficacy or the independent contribution of EGCG. Safety also requires consideration, particularly during development. Experimental studies in DS mouse models indicate that the skeletal effects of EGCG are strongly dependent on dose, treatment duration, developmental timing, and anatomical site. Higher or prolonged exposures have been associated with impaired bone structure, reduced bone mineral density, growth alterations, and exacerbation of selected skeletal phenotypes. These preclinical findings do not establish equivalent skeletal toxicity at clinically studied pediatric doses, but they support caution regarding unsupervised or high-dose supplementation during growth. Overall, EGCG represents a biologically plausible but clinically unconfirmed targeted nutraceutical strategy for DS. The strongest human evidence suggests possible effects on selected cognitive and adaptive domains in young adults when EGCG is combined with cognitive training, whereas pediatric randomized evidence has not demonstrated overall cognitive or functional efficacy. Future studies should clarify optimal formulation, dose, developmental timing, target engagement, long-term skeletal and metabolic safety, and whether biomarker-defined subgroups are more likely to respond [514,515,516,517,518,519,520,521,522,523,524,525,526,527,528,529,530,531,532,533,534,535,536,537].

**Figure 17:**
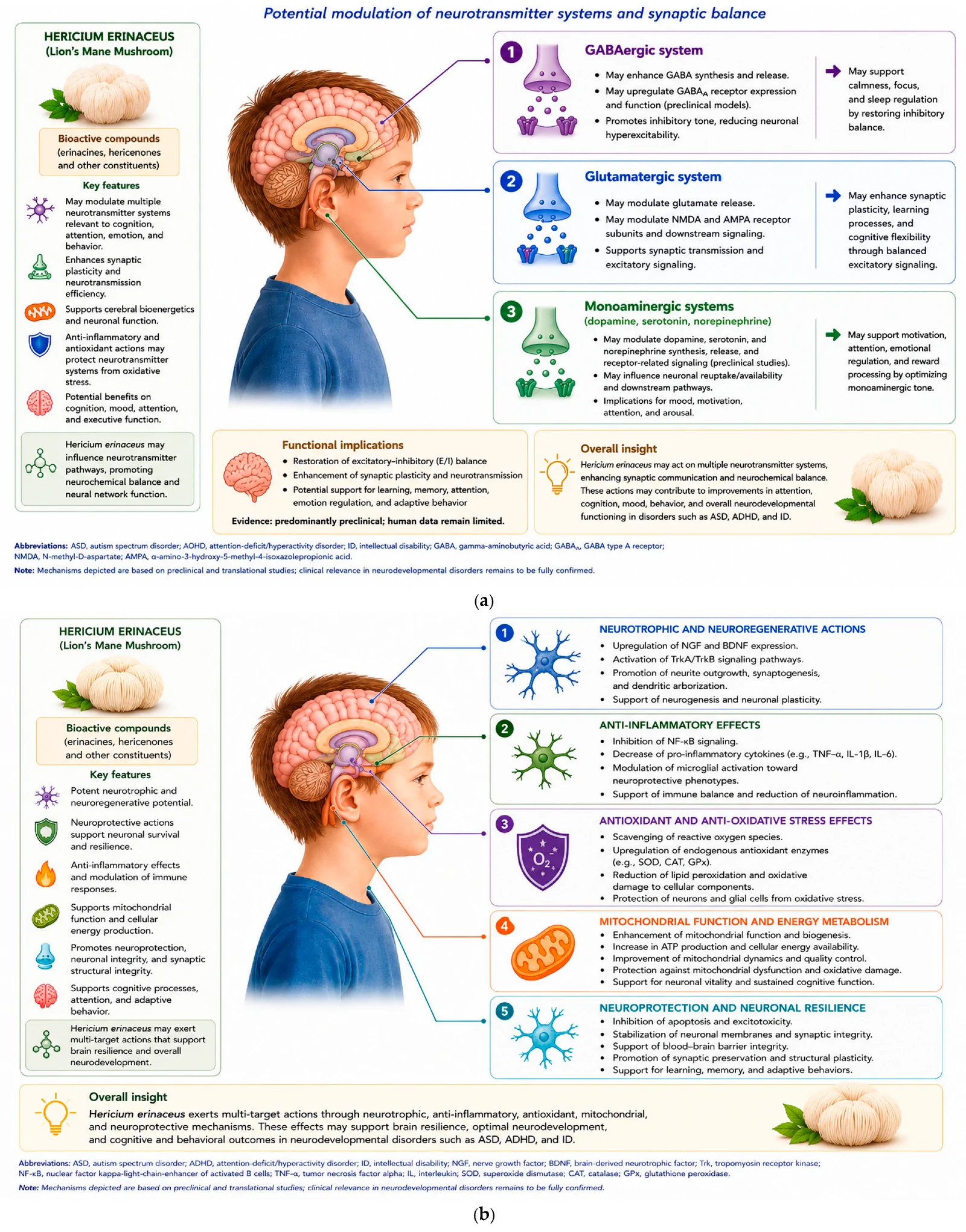

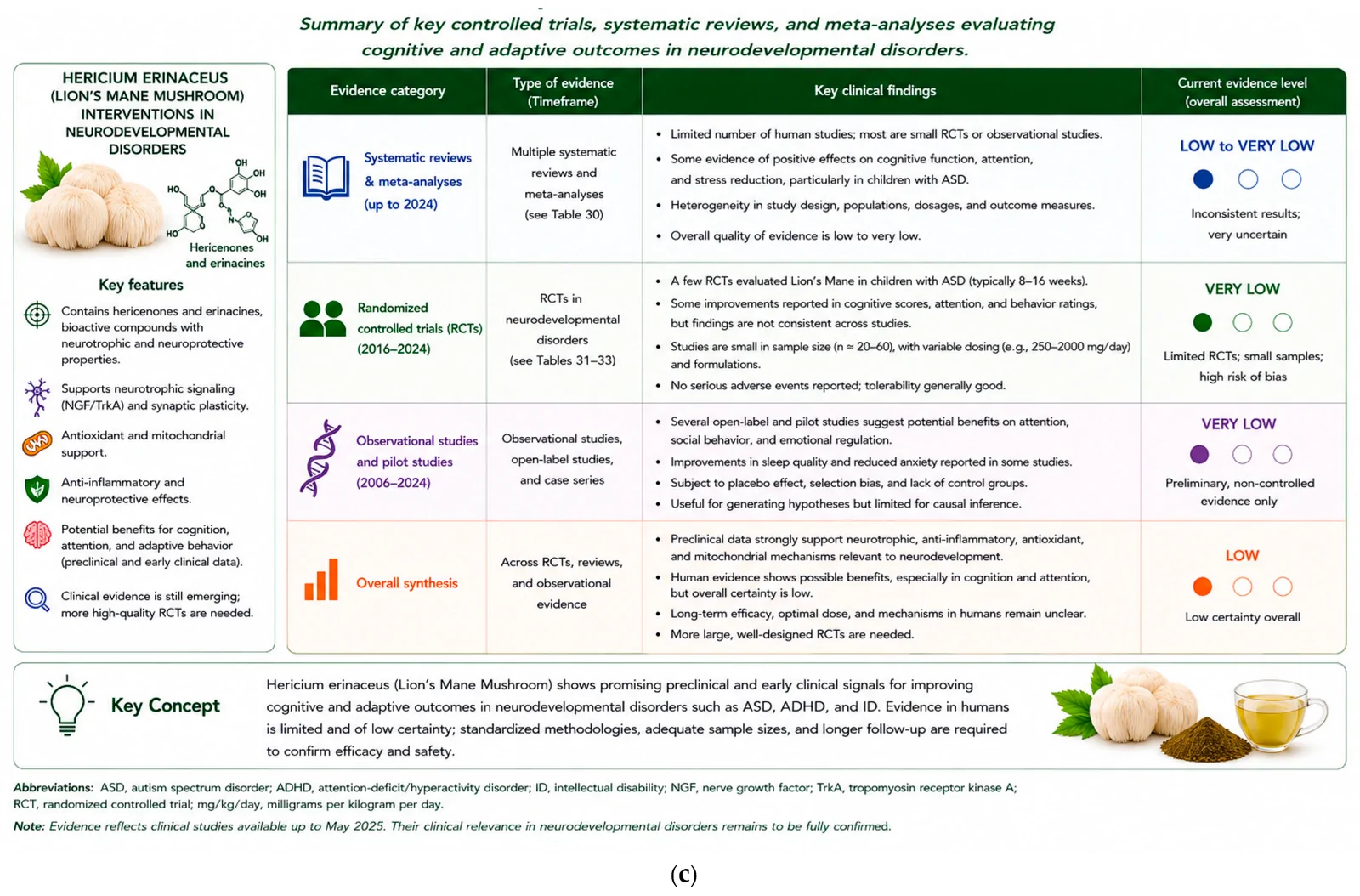
Neurobiological mechanisms and clinical evidence of *Hericium erinaceus* (Lion’s Mane Mushroom) [538,539,540,541,542,543,544,545,546,547,548,549,550,551]. (**a**) Neurotransmitter-related mechanisms of *Hericium erinaceus* in neurodevelopmental disorders. (**b**) Principal non-neurotransmitter mechanisms. (**c**) Summary of current clinical evidence in neurodevelopmental conditions.

*Hericium erinaceus* (Lion’s Mane mushroom) has emerged as a potential neurotrophic and neuroprotective intervention because of bioactive compounds including erinacines and hericenones, which have been investigated for their effects on neurotrophin signaling, neuronal survival, and synaptic plasticity. These neurobiological mechanisms and the available clinical evidence are summarized in Figure 17a–c [538,539,540,541,542,543,544,545,546,547,548,549,550,551]. Specifically, Figure 17a illustrates the principal neurotransmitter-related mechanisms potentially modulated by *Hericium erinaceus* (Lion’s Mane mushroom), while Figure 17b highlights the major non-neurotransmitter mechanisms, including neurotrophic signaling, neuroprotective effects, and pathways involved in neuronal survival and synaptic plasticity. Figure 17c provides an overview of the current clinical evidence regarding the potential therapeutic applications of *Hericium erinaceus* in neurodevelopmental conditions. Preclinical evidence indicates that selected *H. erinaceus* constituents can enhance nerve growth factor (NGF)- and brain-derived neurotrophic factor (BDNF)-related signaling, promote neurite outgrowth, and modulate pathways involved in neuronal survival and plasticity. However, the effects appear compound- and model-dependent, and the frequently proposed direct stimulation of NGF synthesis by all *H. erinaceus* constituents should be interpreted cautiously. Human clinical evidence remains limited and has predominantly involved adults with mild cognitive impairment, age-related cognitive decline, or mood-related outcomes. Recent reviews and human intervention suggest possible benefits on selected measures of cognition and mood, but findings across intervention studies are heterogeneous and constrained by small samples, short treatment durations, differences in preparations and doses, and limited replication. Consequently, evidence obtained in adult cognitive or psychiatric populations cannot be directly extrapolated to pediatric neurodevelopmental disorders. More recently, an observational study investigated *H. erinaceus* biomass supplementation in 147 children with mild-to-borderline intellectual disabilities and high-functioning autistic attributes/characteristics, using quantitative electroencephalography (qEEG) to assess changes in brain activity over 6 and 12 months. The authors reported changes interpreted as improved CNS maturation, including reductions in dominant slow-wave activity and shifts toward beta1 activity. However, the absence of randomization, placebo control, and blinding, together with the reliance on electrophysiological rather than established clinical efficacy endpoints, substantially limits causal interpretation. These findings should therefore be considered preliminary and hypothesis-generating rather than evidence of therapeutic efficacy in ASD or intellectual disability. Overall, *H. erinaceus* has a compelling preclinical neurotrophic rationale involving NGF/BDNF signaling, neuroplasticity, neuroprotection, and anti-inflammatory mechanisms, but clinical evidence in neurodevelopmental disorders remains very limited and of low certainty. Adequately powered randomized placebo-controlled trials using standardized preparations, clinically validated neurodevelopmental outcomes, and long-term safety monitoring are required before therapeutic efficacy can be established in ASD, ADHD, or intellectual disability [538,539,540,541,542,543,544,545,546,547,548,549,550,551].

**Figure 18:**
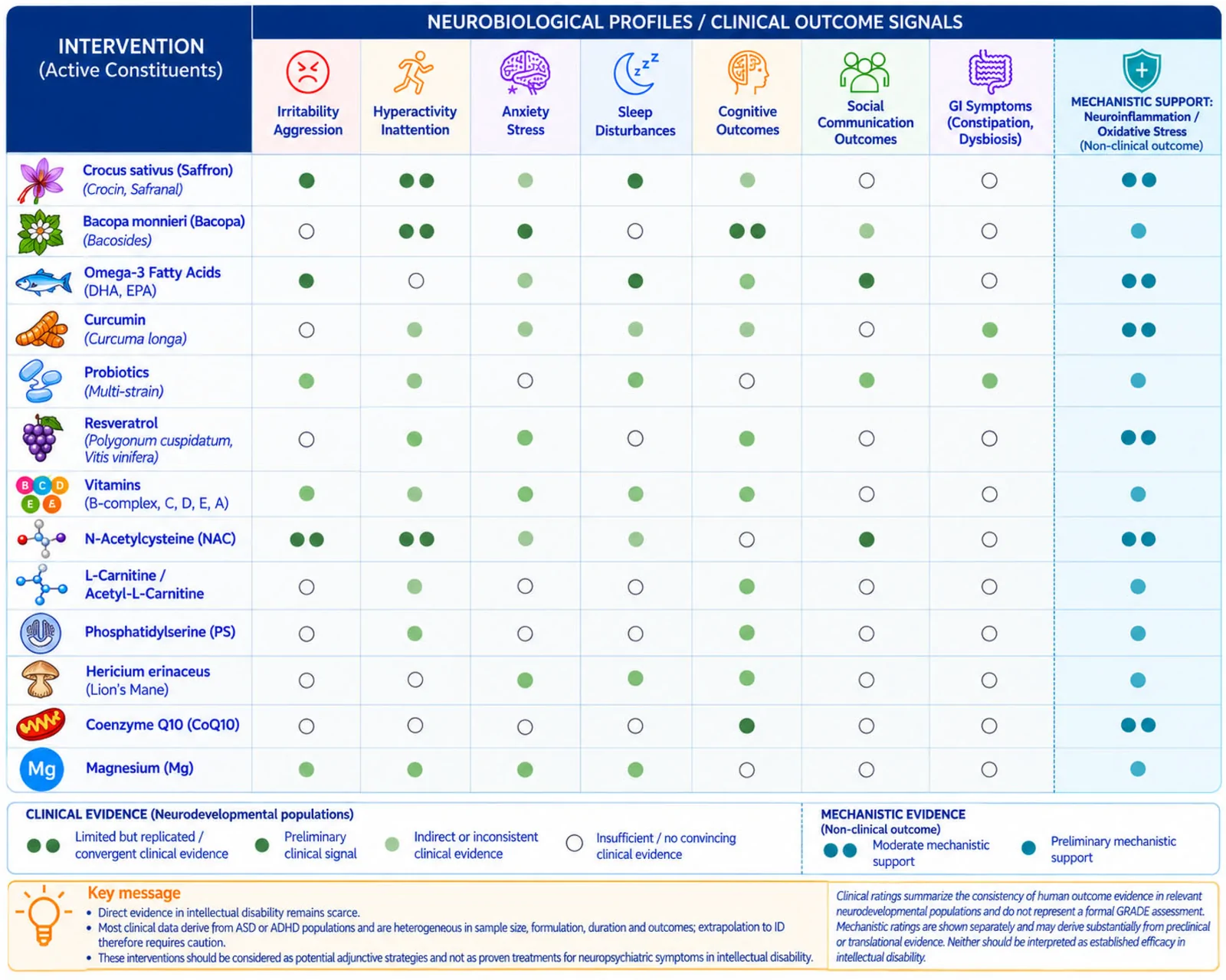
Phytotherapeutics, nutraceuticals and micronutrients in neurodevelopmental disorder associated with ID: potential use in relation to Neurobiological Profile/clinical targets. This table presents a comparative overview of selected nutritional and botanical interventions mapped against key neurobiological profiles and clinical targets relevant to neurodevelopmental health. The interventions are evaluated based on their therapeutic potential, where the number of green dots (1 to 3) represents the relative potency or strength of evidence for each specific clinical target.

**Figure 19:**
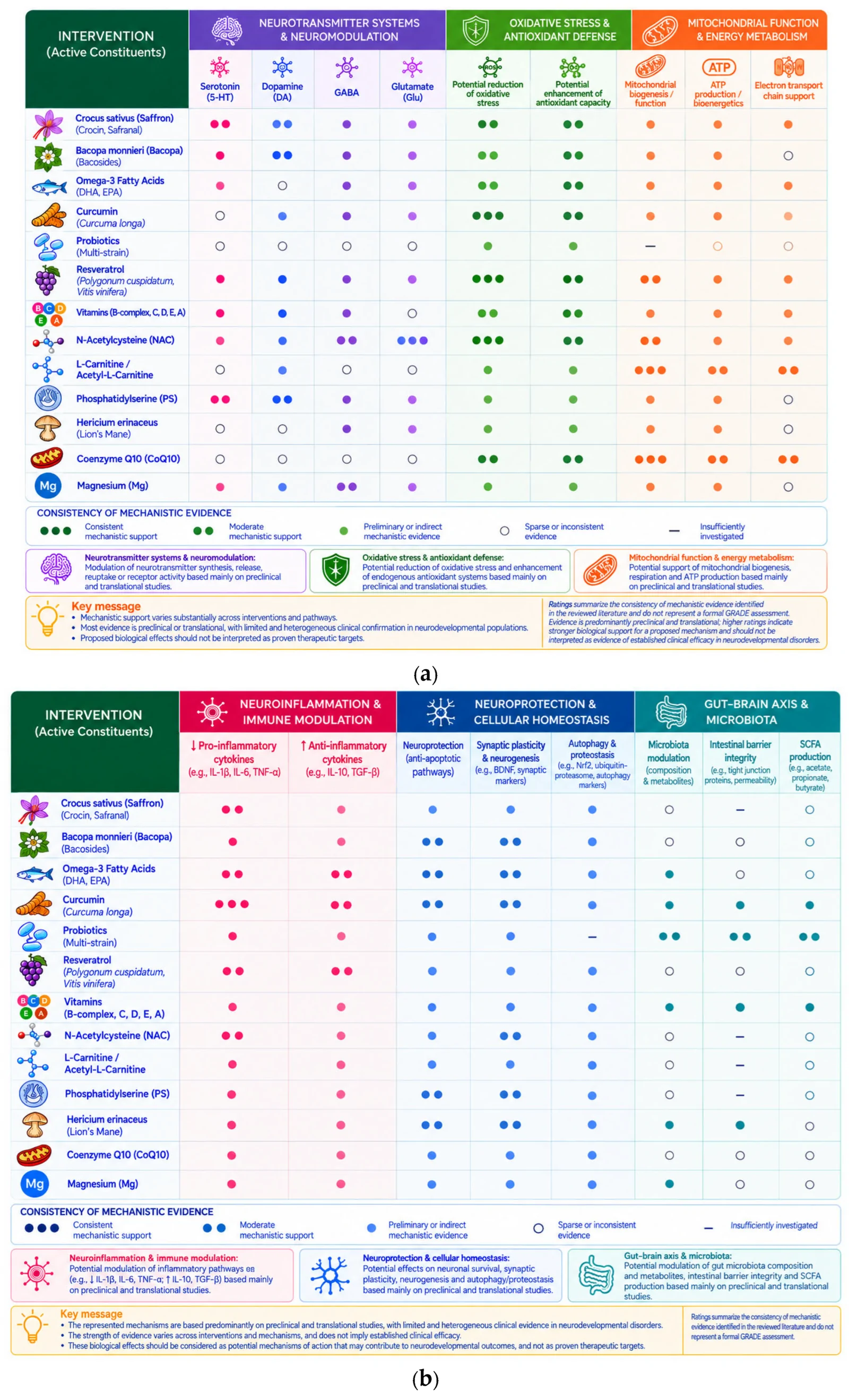
(**a**,**b**) Neurobiological potential targets of phytotherapeutic and micronutrients in neurodevelopmental disorders associated with intellectual disability.

The figure below Figure 18 and Figure 19 summarize the main findings discussed throughout this review regarding the potential role of phytotherapeutics, nutraceuticals, and micronutrients in neurodevelopmental disorders associated with intellectual disability. It highlights their proposed mechanisms of action, targeted neurological profiles and clinical symptoms. Overall, the evidence suggests that these compounds may contribute to improvements in behavioral, cognitive, and neurological outcomes through antioxidant, anti-inflammatory, neurotransmitter-modulating, mitochondrial-supporting, and neuroprotective effects, although the strength of evidence varies among interventions.

## 4. Neurobiological Targets Most Frequently Modulated

Table 29 and Table 30 highlight that most phytotherapeutic and nutraceutical interventions converge on a relatively limited set of interconnected neurobiological pathways implicated in neurodevelopmental disorders associated with intellectual disability. Among these, oxidative stress and neuroinflammatory signaling represent the most frequently targeted mechanisms, consistent with growing evidence implicating redox imbalance and immune dysregulation in the pathophysiology of ASD, ADHD, epilepsy-associated intellectual disability, and related neurodevelopmental conditions. 

Several interventions—including NAC, curcumin, resveratrol, omega-3 fatty acids, coenzyme Q10, and selected vitamins—exert overlapping antioxidant and anti-inflammatory actions. This mechanistic convergence raises the possibility of complementary effects across biological pathways, although potential clinical synergy between these interventions has not been established and should not be inferred from mechanistic overlap alone. Mitochondrial dysfunction and impaired cellular bioenergetics represent another recurrent therapeutic target, particularly for L-carnitine, CoQ10, NAC, and resveratrol. These findings support the hypothesis that mitochondrial abnormalities may contribute to neurodevelopmental phenotypes in biologically defined subgroups, while also emphasizing the need for biomarkers capable of identifying individuals most likely to benefit from mitochondria-targeted interventions. 

The tables (Table 28, Table 29 and Table 30) also illustrate increasing interest in the gut–brain axis, particularly in relation to probiotics and other microbiota-targeted interventions, omega-3 fatty acids, and curcumin. Proposed mechanisms include modulation of gut microbial composition, intestinal barrier integrity, microbial metabolites such as short-chain fatty acids, and peripheral and central immune signaling, which may in turn influence neuroinflammation, cognition, and behavior. However, the extent to which these mechanisms mediate clinically meaningful effects in neurodevelopmental disorders remains uncertain. 

A further point of convergence involves synaptic plasticity and neurotrophic signaling. Interventions such as *Bacopa monnieri*, Hericium erinaceus, and omega-3 fatty acids have been investigated for their potential effects on BDNF- and/or NGF-related pathways, neurite growth, synaptic function, and neuronal resilience. These mechanisms are particularly relevant to learning and memory; however, much of the supporting evidence remains preclinical, and their contribution to clinical outcomes in ASD, ADHD, and intellectual disability has not been firmly established. Overall, Table 29 and Table 30 underscore the multifactorial and interconnected biological architecture of neurodevelopmental disorders and provide a mechanistic rationale for therapeutic strategies that address multiple biological systems rather than isolated molecular or neurotransmitter abnormalities. At the same time, convergence at the mechanistic level should not be equated with demonstrated clinical efficacy. Future studies should determine whether biomarker-guided approaches can identify biologically defined subgroups in whom specific interventions—or rational combinations of interventions—are most likely to provide clinically meaningful benefit. In Table 31, there is a summary of Mechanisms, Clinical Evidence, and Limitations of Nutraceutical Interventions in Neurodevelop-mental Disorders.

## 5. Clinical Evidence in ID-Associated Disorders

To provide a comprehensive and comparative overview of the clinical evidence regarding nutraceutical interventions in neurodevelopmental disorders associated with intellectual disability (ID), Table 31 summarizes the principal biological targets, the strongest available clinical evidence, the main clinical outcomes investigated, the overall levels of evidence, and the key limitations associated with each intervention. This comparative analysis highlights the heterogeneity of the available findings across different neurodevelopmental conditions. Although several nutraceutical compounds exhibit promising neurobiological mechanisms and preliminary clinical signals, the overall strength of the evidence remains variable and frequently limited by small sample sizes, methodological heterogeneity, insufficient long-term follow-up, and a lack of well-designed randomized controlled trials. Furthermore, evidence obtained in broader neurodevelopmental populations cannot necessarily be extrapolated to individuals with intellectual disability. Overall, Table 31 provides an integrated synthesis of the current clinical evidence, emphasizing both the potential therapeutic relevance of these interventions and the need for further rigorous investigations to establish their efficacy, safety, and clinical applicability in ID-associated neurodevelopmental disorders.

**Table 31:** Mechanisms, Clinical Evidence, and Limitations of Nutraceutical Interventions in Neurodevelopmental Disorders.

Intervention	Main Biological Targets	Strongest Clinical Evidence	Clinical Outcomes Investigated/Potential Signals	Level of Evidence	Main Limitations
***Crocus sativus* (Saffron)**	Serotonergic, dopaminergic, GABAergic modulation; antioxidant; anti-inflammatory	ADHD (small pediatric RCTs); anxiety/depression evidence predominantly indirect/adult	Hyperactivity, irritability, sleep, mood dysregulation	Low/preliminary	Limited long-term pediatric data
***Bacopa monnieri***	Cholinergic modulation, neuroprotective activity by increasing BDNF, antioxidant	ADHD, cognitive dysfunction	Attention, memory, executive function	Low/preliminary	Limited pediatric NDD-specific evidence; no direct ID trials
***Omega-3 Fatty Acids (DHA/EPA)***	Membrane fluidity, anti-inflammatory, synaptogenesis	ASD, ADHD	Irritability, hyperactivity, social interaction	low	Heterogeneous formulations/doses
***Curcumin***	NF-κB inhibition, Nrf2 activation, antioxidant	ASD: one recent small randomized controlled trial plus preclinical evidence	Neuroinflammation, oxidative stress, cognition	Very Low/preclinical dominant	Poor bioavailability
***Probiotics***	Gut–brain axis, microbiota modulation, immune regulation	ASD with GI symptoms	Dysbiosis, irritability, behavior	Low	Strain-specific variability
***Resveratrol***	SIRT1 activation, antioxidant, mitochondrial support	ASD: limited human adjunctive/pilot studies plus preclinical evidence	Social deficits, neuroinflammation	Very low	Limited human studies
***Vitamins (B6, B12, D, Folates)***	One-carbon metabolism, methylation, neurotransmitter synthesis	ASD, ADHD	Language, adaptive behavior, irritability	Low–Moderate, intervention-dependent	Biomarker-dependent response
***N-Acetylcysteine (NAC)***	Glutathione precursor, glutamatergic modulation	ASD	Irritability, repetitive behaviors, oxidative stress	Low–moderate	Variable response across studies
***L-Carnitine/Acetyl-L-Carnitine***	Mitochondrial metabolism, fatty acid oxidation	ASD; particular mechanistic relevance to mitochondrial dysfunction	Cognition, adaptive behavior, fatigue	Low	Small sample sizes
***Phosphatidylserine***	Membrane structure/function, phospholipid signaling, synaptic/neurotransmitter-related processes	ADHD	Attention, impulsivity	Low	Limited ASD data
***Hericium erinaceus (Lion’s Mane)***	NGF/BDNF-related signaling, neurite growth, neuroplasticity	Adult cognitive studies; one uncontrolled pediatric observational/qEEG study in mild-to-borderline ID with autistic characteristics	Cognition; neurophysiological/qEEG outcomes; mood in indirect adult studies	Very low	Predominantly preclinical/indirect adult evidence; pediatric study uncontrolled
***Coenzyme Q10 (CoQ10/Ubiquinol)***	Mitochondrial respiration, antioxidant defense	ASD: limited small clinical studies; indirect mitochondrial/redox evidence	Sleep, oxidative stress, adaptive behavior	Very Low–low	Limited controlled trials
***Magnesium***	Voltage-dependent NMDA channel block; modulation of excitatory–inhibitory signaling	ASD, ADHD	Hyperactivity, irritability, sleep	Very low—low	Mostly combined with vitamin B6
***EGCG/Green tea extract***	Putative DYRK1A modulation; redox, mitochondrial and neuroplastic pathways	Down syndrome: TESDAD adult RCT + PERSEUS pediatric RCT	Selected cognitive/adaptive outcomes; no overall pediatric efficacy	Low/inconsistent	Combined intervention in TESDAD; negative overall pediatric efficacy; formulation/dose and developmental safety concerns

Abbreviations: ADHD = Attention-Deficit/Hyperactivity Disorder; ASD = Autism Spectrum Disorder; NDD = Neurodevelopmental Disorder; ID = Intellectual Disability; RCT = Randomized Controlled Trial; GABA = Gamma-Aminobutyric Acid; BDNF = Brain-Derived Neurotrophic Factor; DHA = Docosahexaenoic Acid; EPA = Eicosapentaenoic Acid; NF-κB = Nuclear Factor Kappa B; Nrf2 = Nuclear Factor Erythroid 2-Related Factor 2; GI = Gastrointestinal; SIRT1 = Sirtuin 1; B6 = Vitamin B6; B12 = Vitamin B12; NAC = N-Acetylcysteine; NGF = Nerve Growth Factor; qEEG = Quantitative Electroencephalography; CoQ10 = Coenzyme Q10; NMDA = N-Methyl-D-Aspartate; EGCG = Epigallocatechin Gallate; DYRK1A = Dual-Specificity Tyrosine-Phosphorylation-Regulated Kinase 1A; TESDAD = TEst Safety and efficacy of green tea extract in Down syndrome; PERSEUS = Pediatric trial of EGCG in Down syndrome.

## 6. Safety and Tolerability

Despite the growing interest in phytotherapeutic and nutraceutical interventions for neurodevelopmental disorders associated with intellectual disability (ID), several important clinical limitations must be considered before these agents can be routinely integrated into clinical practice. Although these compounds are generally well-tolerated, they are not devoid of risks and require caution due to potential drug-nutrient interactions and marked variability in supplement quality. Because pediatric populations are characterized by physiological fragility and are frequently subject to polypharmacotherapy, they represent a particularly vulnerable group. Consequently, clinical monitoring and appropriate professional supervision should be considered, particularly in medically complex pediatric populations, when these approaches are implemented as adjunctive strategies [552,553,554].

A primary concern involves the potential for pharmacokinetic and pharmacodynamic drug–nutraceutical interactions. Many phytochemicals and nutraceuticals interact with hepatic cytochrome P450 enzymes and membrane transporters, thereby altering the plasma concentration of concomitant medications. For example, *Crocus sativus* (saffron) exerts significant serotonergic activity; its concurrent use with selective serotonin reuptake inhibitors (SSRIs), which are commonly prescribed for anxiety and mood dysregulation in ID, may theoretically potentiate serotonergic neurotransmission and increase the risk of serotonin syndrome [555,556]. Similarly, *Ginkgo biloba*—often sought for cognitive enhancement—is well-documented for its antiplatelet and anticoagulant properties, which present a clinical risk of hemorrhage if co-administered with anticonvulsant drugs like valproic acid or anticoagulants, often utilized in individuals with ID-associated epilepsy [557,558]. Furthermore, *Curcuma longa* (curcumin) has been shown to inhibit CYP3A4 and P-glycoprotein, pathways that are critical for the metabolism of numerous antipsychotics and antiepileptic agents. The inhibitory effect of curcumin on these pathways can lead to increased serum levels of psychotropic medications, potentially exacerbating dose-dependent adverse effects such as sedation or metabolic disturbances [559,560].

Beyond interaction risks, dosage variability and the lack of standardization across commercial formulations remain major limitations. Considerable heterogeneity exists regarding extraction methods, bioavailability, purity, active constituent concentration, treatment duration, and administered doses. This variability is particularly relevant for phytotherapeutics such as curcumin, saffron, *Bacopa monnieri*, resveratrol, and probiotics, whose biological effects are strongly dependent on formulation characteristics and pharmacokinetic properties [561,562,563]. For instance, the therapeutic potential of curcumin is severely hampered by poor oral bioavailability; however, the addition of bioenhancers like piperine (black pepper extract) to overcome this limitation can simultaneously inhibit the metabolism of a wide range of other drugs, creating an unintended secondary risk of drug toxicity. Probiotics present a different set of challenges, as their efficacy and safety are highly strain-specific. Clinicians must be aware that findings from one specific strain cannot be extrapolated to others, and the administration of high-dose microbial strains to patients with compromised intestinal barriers or immune deficiencies requires cautious evaluation to prevent the rare but severe risk of translocation and bacteremia [562,563]. The absence of universally standardized preparations therefore limits reproducibility and may contribute to inconsistent clinical findings.

Regarding pediatric safety and neurodevelopmental vulnerability, the lack of long-term data represents a significant knowledge gap. While acute adverse effects such as gastrointestinal discomfort, diarrhea, or mild agitation are commonly documented and typically transient, the long-term impact of chronic nutraceutical exposure on the developing brain-during the highly sensitive periods of synaptogenesis and pruning-remains unknown. Children with ID and associated comorbidities often exhibit reduced metabolic reserves, and their response to exogenous substances may differ significantly from neurotypical pediatric populations. For example, excessive or poorly monitored magnesium supplementation in patients with even mild renal insufficiency can induce hypermagnesemia, manifesting as lethargy, hypotonia, or cardiac arrhythmias. Furthermore, practitioners should maintain high vigilance for signs of endocrine or immunological disruption, as many antioxidants and immunomodulatory compounds can potentially shift the delicate redox balance or immune set-points in ways that are not yet fully elucidated through pediatric longitudinal studies [564,565].

Finally, quality control concerns in commercial supplements further complicate clinical application. Nutraceutical products may exhibit substantial variability in purity, potency, contamination risk, and labeling accuracy. Independent analyses have occasionally identified discrepancies between declared and actual concentrations of active compounds, as well as contamination with heavy metals, pesticides, solvents, or undeclared pharmaceutical substances [46,566]. This issue is especially relevant in pediatric populations and in individuals with ID who may require prolonged, daily supplementation. In addition, regulatory oversight for dietary supplements is generally less stringent than for pharmaceutical medications in many countries, leading to inconsistencies in manufacturing standards and product reliability.

Overall, while phytotherapeutics and nutraceuticals represent promising adjunctive approaches, their clinical application should currently be considered complementary rather than replacement therapy. Future research should prioritize larger randomized controlled trials, biomarker-guided patient stratification, long-term safety evaluation, pharmacokinetic studies, and international standardization of nutraceutical formulations to improve both scientific rigor and clinical applicability.

## 7. Limitations of Current Evidence

The current evidence on phytotherapeutics and nutraceuticals in neurodevelopmental disorders associated with intellectual disability is limited by several important methodological weaknesses. First, there is a scarcity of clinical trials specifically targeting populations with intellectual disability, since most studies primarily focus on ASD or ADHD without adequately stratifying patients according to cognitive impairment severity. Moreover, many studies involve small sample sizes and short treatment durations, reducing statistical power and limiting the evaluation of long-term efficacy and safety. Another major limitation is the marked heterogeneity in formulations, dosages, and study designs, which complicates comparisons across trials and contributes to inconsistent findings. Finally, most studies lack biomarker-guided approaches, despite growing evidence that treatment response may depend on underlying biological phenotypes such as oxidative stress, mitochondrial dysfunction, neuroinflammation, or gut dysbiosis. Together, these limitations highlight the need for larger, standardized, biomarker-driven clinical trials specifically designed for individuals with intellectual disability.

## 8. Future Directions

Future research in neurodevelopmental disorders associated with intellectual disability should prioritize large-scale randomized controlled trials involving well-characterized clinical and biological subgroups. Particular attention should be directed toward biomarker-driven and precision medicine approaches capable of identifying metabolically and genetically defined responder phenotypes. In addition, combination therapies targeting multiple interconnected biological pathways—including oxidative stress, neuroinflammation, mitochondrial dysfunction, neurotransmitter imbalance, and gut–brain axis alterations—may represent a promising therapeutic strategy. Given the chronic nature of these conditions and the developmental vulnerability of pediatric populations, long-term safety and tolerability studies are also essential to establish the efficacy, sustainability, and clinical applicability of phytotherapeutic and nutraceutical interventions.

## 9. Conclusions

In conclusion, this review highlights the potential role of phytotherapeutics, nutraceuticals, and micronutrients as adjunctive interventions in neurodevelopmental disorders associated with intellectual disability, particularly ASD and ADHD, while emphasizing that robust clinical evidence specifically in populations with ID remains scarce. Current evidence identifies oxidative stress, neuroinflammation, mitochondrial dysfunction, neurotransmitter dysregulation, synaptic and neurotrophic signaling, and gut–brain axis alterations as recurrent and interconnected biological targets potentially modulated by these interventions. Among the compounds reviewed, omega-3 fatty acids, N-acetylcysteine (NAC), selected vitamins and vitamin-related interventions—particularly folinic acid and vitamin D—probiotics, and saffron have accumulated comparatively greater clinical evidence in specific neurodevelopmental populations or symptom domains, although the overall certainty of evidence remains generally low to moderate and varies substantially across interventions. Other compounds, including curcumin, resveratrol, *Bacopa monnieri*, coenzyme Q10, L-carnitine, magnesium, phosphatidylserine, and *Hericium erinaceus*, are supported predominantly by preliminary clinical, mechanistic, or preclinical evidence and therefore require further validation. Across the literature, interpretation is constrained by small sample sizes, methodological heterogeneity, inconsistent formulations and dosing regimens, limited biomarker stratification, and insufficient long-term safety data. Accordingly, phytotherapeutics and nutraceuticals should currently be considered potential complementary rather than replacement strategies and, when used clinically, should be integrated within individualized multidisciplinary care. Future adequately powered randomized controlled trials, biomarker-guided patient stratification, standardized intervention protocols, and long-term safety studies will be essential to identify responder phenotypes, establish clinically meaningful efficacy, and determine whether mechanistic promise can translate into reproducible therapeutic benefit.

## Figures and Tables

**Table 1:** Clinical Studies Evaluating *Crocus sativus*.

Reference	Population (N)	Study Type	Dose & Duration	Primary Endpoints	Key Results	Study Limitations/Risk of Bias/Notes
Blasco-Fontecilla et al., 2022 [43]	Children/Adolescents with ADHD (N = 63)	Open-label Pilot Clinical study	15–30 mg/day (12 wks), Saffron extract	Hyperactivity, Sleep quality	Symptom improvement	Small N; lack of placebo control; greater effect on sleep disturbances
Khaksarian et al., 2021 [44]	Children (N = 66 completed) 70 enrolled (4 drop-outs)	RCT (Add-on) Randomized double-blind study	Methylphenidate + saffron (~20–30 mg/day, 6–8 wks)	ADHD symptoms	Symptom improvement reported with adjunctive saffron; additional benefit requires confirmation	Small N; limited follow-up

Abbreviations: ADHD, Attention-Deficit/Hyperactivity Disorder; N, Total number of participants; RCT, Randomized Controlled Trial.

**Table 2:** Secondary Research Evaluating *Crocus sativus*.

Reference	Population (N)	Study Type	Dose & Duration	Primary Endpoints	Key Results	Study Limitations/Risk of Bias/Notes
Ross, 2020 [42]	Mixed (ADHD Pediatric)	Narrative Review	20–30 mg/day	ADHD Symptoms	Symptom improvement	Lack of original data; high potential for selection bias; secondary analysis
Seyedi-Sahebari et al., 2024 [45]	Children (N = 118 approx)	Systematic Review Of RCTs	20–30 mg/day (6–12 wks)	ADHD scales	Symptom reduction, generally good tolerability	Methodological heterogeneity; variable extract purity
Rigillo et al., 2025 [46]	Pediatric mental disorders (Review)	Narrative Review	20–30 mg/day	ADHD Symptoms	Preliminary findings suggesting potentially similar symptom improvement to MPH in limited available studies	Qualitative synthesis; lacks standardized methodology; integrative perspective

Abbreviations: ADHD, Attention-Deficit/Hyperactivity Disorder; MPH, Methylphenidate; N, Total number of participants; RCT, Randomized Controlled Trial.

**Table 3:** Clinical Studies Evaluating *Crocus sativus* in Adults with Depression.

Reference	Population (N)	Study Type	Dose & Duration	Primary Endpoints	Key Results	Study Limitations/Risk of Bias/Notes
Akhondzadeh et al., 2004 [47]	Adults (N = 40)	RCT vs. imipramine	30 mg/day (6 weeks)	Depression severity	Similar short-term symptom improvement to imipramine	Small N; pilot study
Akhondzadeh et al., 2005 [48]	Adults (N = 40)	RCT vs. placebo	30 mg/day (6 weeks)	HAM-D score	Significant improvement vs. placebo	Landmark trial; limited sample
Noorbala et al., 2005 [49]	Adults (N = 40)	RCT vs. fluoxetine	30 mg/day (6 weeks)	Depression scores	Similar short-term symptom improvement to fluoxetine	Generally well-tolerated; small sample, short-term study.
Talaei et al., 2015 [52]	Adult (N = 60)	RCT vs. placebo	30 mg/day (purified crocin—4 weeks)	Depression scores	Significant improvement	Evaluated pure bioactive constituent (crocin) rather than total extract; special clinical population; limited sample size.

Abbreviations: N, Total number of participants; RCT, Randomized Controlled Trial. HAM-D: Hamilton Depression Rating Scale.

**Table 4:** Secondary Research Evaluating *Crocus sativus* in Depression.

Reference	Population (N)	Study Type	Dose & Duration	Primary Endpoints	Key Results	Study Limitations/Risk of Bias/Notes
Hausenblas et al., 2013 [50]	Mixed Depression (N = 200 randomized—177 analyzed aggregated)	Meta-analysis of RCTs (5RCTs)	~30 mg/day (6 wks)	Depression severity	Moderate-to-large effect size	Limited number and size of primary studies; potential between-study heterogeneity.
Lopresti & Drummond, 2014 [51]	Mixed Depression (N = 250 randomized—230 analyzed aggregated)	Systematic review/meta-analysis (6RCTs)	30 mg/day typical (4–8 wks)	Mood outcomes	Antidepressant effect. Mapped multi-target pathways (serotonergic, antioxidant, and anti-inflammatory).	Lack of long-term follow-up data; significant variations in herbal extract standardization across primary studies.

Abbreviations: N, total number of participants; RCT, Randomized Controlled Trial.

**Table 5:** Clinical Studies Evaluating *Crocus sativus* L. in Neuropsychiatric Disorders.

Reference	Population (N)	Study Type	Dose & Duration	Primary Endpoints	Key Results	Study Limitations/Risk of Bias/Notes
Lopresti et al., 2017 [53]	Adults with major depression (N = 123)	RCT	Curcumin extract (500 mg/day) + Saffron extract (30 mg/day) for 12 weeks	Depressive symptoms (IDS-SR30), anxiety (STAI)	Significant clinical improvement in mood and anxiety vs. placebo; findings derived from combined curcumin–saffron supplementation	Lack of a saffron-only monotherapy arm to isolate the incremental benefit of saffron; reliance on self-report rating scales.
Kell et al., 2017 [58]	Healthy adults self-reporting low mood (N = 128)	RCT (Double-blind, parallel-group)	Saffron extract (affron) at 22 mg/day or 28 mg/day for 4 weeks	Mood disturbance (POMS), anxiety, sleep quality	Significant reduction in negative mood and anxiety scores at the 28 mg/day dose	Short intervention duration; exclusion of clinically diagnosed psychiatric populations (subclinical cohort); reliance on self-reported measures.

Abbreviation: IDS-SR30, Inventory of Depressive Symptomatology; POMS, Profile of Mood States; RCT, Randomized Controlled Trial; STAI, Spielberger State–Trait Anxiety Inventory.

**Table 6:** Secondary Research for *Crocus sativus*.

Reference	Scope/Included Studies	Study Type	Dose (Typical)	Primary Endpoints	Key Findings	Study Limitations/Risk of Bias/Notes
Tóth et al., 2019 [54]	Patients with mild-to-moderate depression (9 RCTs included)	Systematic review and meta-analysis	30 mg/day	Depression severity scales	Reduction in depressive symptoms vs. placebo; no significant difference from antidepressant comparators in the included trials.	Small sample sizes within primary studies; high reliance on trials conducted within restricted geographic regions (potential replication bias).
Dai et al., 2020 [55]	Patients with mild-to-moderate depression (12 RCTs included)	Systematic review and meta-analysis	20–30 mg/day	Hamilton Depression Rating Scale (HAM-D)/Beck Depression Inventory (BDI)	Improvement in depressive symptoms vs. placebo; no significant difference relative to antidepressant comparators in the included trials; generally comparable tolerability.	Short duration of included trials; limited sample sizes; authors note a strict need for multicenter studies across diverse ethnic groups to verify generalizability.
Musazadeh et al., 2022 [56]	Depressive disorders (7 meta-analyses aggregated)	Umbrella meta-analysis	Typically 30 mg/day	Standardized depression scores (BDI, HAM-D)	Reduction in BDI scores was observed with adjunctive saffron.	Significant reduction observed specifically on the BDI scale, while variations on the HAM-D were non-significant in the global pooling; cannot be considered a standalone monotherapy.

Abbreviations: N, Total number of participants; RCT, Randomized Controlled Trial. HAM-D: Hamilton Depression Rating Scale, BDI: Beck Depression Inventory.

**Table 7:** Clinical Studies Evaluating *Bacopa monnieri*.

Reference	Population (N)	Study Type	Dose & Duration	Primary Endpoints	Key Results	Study Limitations/Risk of Bias
Roodenrys et al., 2002 [102]	Healthy adults (N = 76, aged 40–65)	RCT (Double-blind, placebo-controlled)	Bacopa extract: 300 mg/day if body weight <90 kg; 450 mg/day if >90 kg; 12 weeks	Memory functions, including retention of newly learned information; attention; verbal and visual short-term memory; learning; anxiety	Improvement in retention of newly learned information vs. placebo; learning rate was unaffected, suggesting a reduced rate of forgetting. Attention, verbal and visual short-term memory, retrieval of pre-existing knowledge, everyday memory and anxiety were not significantly affected.	Healthy middle-aged adults; therefore, evidence is indirect and cannot be directly generalized to pediatric neurodevelopmental disorders or intellectual disability.
Raghav et al., 2006 [103]	Elderly adults with Age-Associated Memory Impairment (N = 40 enrolled, 35 completed)	RCT (Double-blind, placebo-controlled)	250 mg/day (12 weeks), standardized extract	Logical memory, paired associate learning	Improvement in logical memory and paired associate learning scores compared to placebo.	Small sample size; sample is strictly specific to age-related cognitive decline rather than developmental deficits.
Calabrese et al., 2008 [104]	Healthy elderly individuals aged 65+ (N = 103 randomized, 81 completed)	RCT (Double-blind, placebo-controlled)	300 mg/day (12 weeks), standardized extract	Delayed word recall, attention, anxiety, and depression scales	Improvement in delayed word recall; significant reduction in anxiety and depression scores vs. placebo.	Small completed sample size; geriatric non-ID cohort; reliance on subclinical mood scores.
Dave et al., 2008 [101]	Children requiring an individual education programme; IQ 70–90 (N = 28 enrolled)	Clinical trial	BacoMind^®^ 225 mg/day for 4 months	Working memory; short-term verbal memory; logical, personal-life, visual, and auditory memory	Improvements from baseline in several memory domains; treatment was reported as well tolerated	Small sample; limited methodological information; no clear placebo-controlled comparison; population not specifically diagnosed with ADHD or ID
Peth-Nui et al. (2012) [105]	Healthy elderly volunteers aged 55–80 (N = 60)	Clinical Trial (Primary data)	300 mg/day (12 weeks)	Attention, Cognitive Processing, Working Memory, and Functions of Both Monoamine Oxidase and Acetylcholinesterase	Suppression of acetylcholinesterase (AChE) activity; improved attention and working memory.	Small, single-center sample.
Dave et al., 2014 [110]	Children with ADHD aged 6–12 years (N = 31 completed)	Open-label clinical study	225 mg/day (for <30 kg) or 300 mg/day (for >30 kg) for 6 months	ADHD symptom scales, learning capacity, and behavioral regulation	Within-group reductions in ratings of restlessness, inattention, and impulsivity after 6 months.	Open-label design with no placebo control arm; single-center study; subjective parental/investigator rating scales.
Kean et al., 2022 [108]	Boys aged 6–14 years with inattention/hyperactivity (N = 112 recruited; 93 analyzed)	Randomized, double-blind, placebo-controlled trial	CDRI 08^®^; 14-week trial	Behavioral, cognitive, mood, and sleep outcomes	No significant between-group differences in behavioral outcomes; exploratory improvements in selected cognitive, executive, interpersonal, and sleep measures	Primary behavioral benefit not demonstrated; several positive findings involved secondary outcomes; further confirmatory trials required

Abbreviations: N, total number of participants; RCT, Randomized Controlled Trial; ID: Intellectual Disability; ADHD: Attention Deficit/Hyperactivity Disorder; AChE: Acetylcholinesterase.

**Table 8:** Secondary Research and Preclinical Evidence for *Bacopa monnieri*.

Reference	Scope/Model/Population	Study Type	Dose (Reported/Typical)	Key Findings	Study Limitations/Risk of Bias/Notes
Uabundit et al., 2010 [106]	AF64A-induced cholinergic degeneration in Wistar Rats	Preclinical (Animal study)	20, 40, and 80 mg/kg (up to 2 weeks)	Dose-dependent reduction in hippocampal neuronal loss; significant mitigation of oxidative stress.	Animal model of cholinergic deficit; lacks translational data regarding complex human neurodevelopment.
Kongkeaw et al., 2014 [109]	Cognitive performance in adults (9 RCTs; pooled N = 518)	Systematic Review & Meta-analysis	~300 mg/day (Standardized extract)	Improvement in selected cognitive outcomes, particularly speed of attention; potential effects on memory-related measures.	Substantial methodological heterogeneity among primary trials; variations in extract standardization protocols.
Kean et al., 2016 [111]	Children and adolescent populations (5 pediatric studies)	Systematic Review	~300 mg/day (or weight-stratified)	Indicates potential for improving long-term memory retrieval in youth; no major safety concerns reported in the included studies	Qualitative synthesis due to lack of homogeneous quantitative data; limited availability of rigorous pediatric RCTs.

Abbreviations: N, total number of participants; RCT, Randomized Controlled Trial.

**Table 9:** Clinical Studies Evaluating *Curcuma longa* (Curcumin).

Reference	Population (N)	Study Type	Dose & Duration	Primary Endpoints	Key Results	Study Limitations/Risk of Bias
Lopresti et al., 2014 [132]	Adults with Major Depressive Disorder (N = 56)	RCT (Double-blind, placebo-controlled)	1000 mg/day (8 weeks)	Depressive symptoms (IDS-SR30 score), cognitive anxiety markers	Improvements in mood and cognitive disturbances vs. placebo, with therapeutic effects emerging predominantly between weeks 4 and 8.	Small sample size; short intervention window; sample restricted to adult unipolar depression (lacks direct pediatric translation).
Vergara Serpa & Sumalla Cano, 2026 (CURATEA) [136]	Children and adolescents with Autism Spectrum Disorder (N = 60)	RCT (Double-blind, placebo-controlled)	3000 mg/day (8 weeks)	Inflammatory biomarkers (hs-CRP, ESR, fecal calprotectin); sensory regulation scales (IGDRPS); GI symptom scores	Reduction in hs-CRP (−83.8%) and fecal calprotectin (−73.7%); 49% improvement in sensory-processing measures; 90% resolution of functional GI comorbidities.	Single-center study; modest sample size; short treatment duration; independent multicenter replication and longer-term safety data are required.

Abbreviations: N, total number of participants; RCT, Randomized Controlled Trial; IDS-SR30: Inventory of Depressive Symptomatology—Self-Report; GI: Gastrointestinal; IGDRPS: General Index of Sensory Processing Regulation Dysfunction.

**Table 10:** Secondary Research and Preclinical Evidence for *Curcuma longa*.

Reference	Scope/Model/Dataset	Study Type	Dose (Reported/Typical)	Key Findings	Limitations/Risk of Bias/Notes
Bharal et al., 2008 [131]	PTZ and MES-induced seizure models in mice	Preclinical (Animal study)	50, 100, and 200 mg/kg/day	Dose-dependently increased the seizure threshold; reduced lipid peroxidation and restored endogenous antioxidant enzyme activity.	Animal model of acute seizure induction; difficult to translate directly to chronic developmental encephalopathies or ID.
Bhandari and Kuhad [135]	Propionic acid (PPA)-induced neurodevelopmental model of Autism (Rats)	Preclinical (Translational animal model)	50, 100, and 200 mg/kg/day for 4 weeks	Reversed ASD-like behavioral abnormalities; suppressed TNF-alpha and neuroinflammation; restored depleted mitochondrial complex (I–IV) activities.	Preclinical rodent model of ASD-related phenotypes; lacks direct human pediatric validation
Mythri & Bharath, 2012 [139]	Neuroprotective pathways in Parkinson’s Disease models	Narrative Literature Review	Not applicable	Outlined curcumin’s capacity to protect dopaminergic neurons, mitigate mitochondrial Complex I deficiency, and block apoptotic cascades.	Monographic focus on adult dopaminergic neurodegeneration (Parkinson’s); application to pediatric neurodevelopment is indirect.
Ng et al., 2017 [133]	Major Depressive Disorder (6 RCTs; total pooled N = 377)	Systematic Review & Meta-analysis	500–1000 mg/day	Reductions in depressive and anxiety symptoms relative to placebo in pooled adult studies of curcumin vs. placebo; generally well-tolerated across cohorts.	Small total pooled sample size (N = 377); short duration of primary trials; data are derived from adult psychiatric cohorts.
Hewlings & Kalman, 2017 [134]	Systemic inflammation and oxidative stress in human health	Comprehensive Narrative Review	500–2000 mg/day across general literature	Documented cross-target reduction in NF-kB activation and circulating cytokines; highlights co-administration with piperine to enhance bioavailability.	Narrative synthesis lacking statistical pooling (meta-analysis); emphasizes that poor absorption remains a major hurdle.

Abbreviations: PTZ: Pentylenetetrazole; MES: Maximal Electroshock; PPA: Propionic Acid Induced; N: total number of participants; RCT: Randomized Controlled Trial; NFkB: Nuclear Factor kappa-light-chain-enhancer of activated B cells; ID: Intellectual Disability; ASD: Autism Spectrum Disorder.

**Table 11:** Clinical Studies Evaluating N-acetylcysteine (NAC) in ASD.

Reference	Population (N)	Study Type	Dose & Duration	Primary Endpoints	Key Results	Study Limitations/Risk of Bias/Notes
Ghanizadeh & Derakhshan, 2012 [165]	Child with ASD (N = 1)	Case Report	~800 mg/day for 8 weeks	Behavioral symptoms, irritability scales	Behavioral improvements, reduction in hyperactivity, and enhanced compliance.	Single case report; uncontrolled design; findings cannot be generalized or used to infer efficacy
Hardan et al., 2012 [166]	Children with ASD (Total 40—N = 33 completed)	RCT (Double-blind, placebo-controlled pilot trial)	Standardized oral NAC escalated up to 2700 mg/day for 12 weeks	Irritability scores (Aberrant Behavior Checklist—ABC)	Reduction in irritability scores and hyperactivity subscales compared to the placebo group.	Small pilot study; single-center design; short-term follow-up; participants with and without ID were included, but ID-specific outcomes were not evaluated
Nikoo et al., 2015 [167]	Children and adolescents with ASD (N = 40)	RCT (Double-blind, placebo-controlled adjunctive trial)	NAC (1200 mg/day) + Risperidone (1–2 mg/day) for 8 weeks	Irritability subscale (ABC-I)	Greater reduction in irritability with NAC + risperidone than with risperidone + placebo.	Concomitant medication design (difficult to isolate the standalone efficacy of NAC); short trial duration; potential selection bias.
Wink et al., 2016 [168]	Youth with ASD (N = 31 randomized—28 completed)	RCT (Double-blind, placebo-controlled pilot study)	Standardized oral NAC escalated up to 2700 mg/day for 12 weeks	Brain glutathione (GSH) levels via MRS; Social behavior scales	Increased cortical glutathione levels; generally well tolerated; no significant improvement in core social impairment	Small pediatric sample size; mixed clinical efficacy profile; heavy reliance on subjective parental rating scales despite objective biomarker tracking.
Pesko et al., 2020 [169]	Pediatric patients with ASD (N = 4)	Clinical Case Series	Variable individualized dosing over retrospective timelines	Irritability patterns, behavioral stabilization	Reductions in severe irritability were reported during NAC supplementation.	Retrospective case series design; extremely small sample footprint (N = 4); total lack of control group or blinding protocols.

Abbreviations: ASD = Autism Spectrum Disorder; NAC = N-Acetylcysteine; RCT = Randomized Controlled Trial; ABC = Aberrant Behavior Checklist; ABC-I = Aberrant Behavior Checklist–Irritability Subscale; GSH = Glutathione; MRS = Magnetic Resonance Spectroscopy; ID = Intellectual Disability; N = number of participants; mg/day = milligrams per day.

**Table 12:** Secondary Research and Preclinical Evidence for N-acetylcysteine (NAC).

Reference	Scope/Model/Dataset	Study Type	Dose & Duration	Primary Endpoints	Key Findings	Study Limitations/Risk of Bias/Notes
Lee et al., 2021 [170]	Children and adolescents with ASD (Pooled N = 237)	Systematic Review & Meta-analysis of RCTs	900–2700 mg/day (typical primary ranges)	Hyperactivity subscales, irritability indices, social responsiveness	Modest improvements in hyperactivity and irritability; generally well tolerated.	Substantial methodological heterogeneity across included primary trials; highly variable treatment durations and extract criteria.
Zhang et al., 2023 [172]	Valproic acid (VPA)-induced model of Autism (Rats)	Preclinical (Animal study)	Variable doses (systemic injection regimens)	Autophagic biomarkers, oxidative stress status, autism-like behaviors	Attenuated autism-like behavioral abnormalities; restored hippocampal autophagic activity and downregulated Notch-1/Hes-1 signaling	Preclinical rodent model; translation to human neurodevelopmental disorders remains uncertain
Pangrazzi et al., 2024 [173]	*Shank3b*-knockout syndromic model of Autism (Mice)	Preclinical (Translational molecular study)	Variable doses administered via experimental timelines	Cerebellar neuroinflammation, microglial activation, social interactions	Attenuated cerebellar inflammation and microglial activation and improved sociability-related measures.	Mechanistic rodent design; explicitly restricted to genetic, syndromic ASD pathways; cannot be generalized to idiopathic human autism phenotypes.
Ramírez-Guerrero et al., 2025 [171]	Antioxidant-based interventions in ASD (20 clinical studies; 3 RCTs evaluating NAC)	Systematic Review	Heterogeneous across included studies	ASD-related behavioral and psychiatric symptoms, including irritability, repetitive behavior, social cognition, and hyperactivity	NAC showed Improvement in selected behavioral domains, particularly irritability, repetitive behavior, social cognition, and hyperactivity; findings were inconsistent across trials.	Heterogeneity in interventions, dosing regimens, outcome measures, and sample sizes; risk of bias in several included studies; insufficient evidence to support antioxidant therapies as monotherapy for ASD.

Abbreviations: ASD = Autism Spectrum Disorder; RCT = Randomized Controlled Trial; VPA = Valproic Acid; NAC = N-Acetylcysteine; N = number of participants; *Shank3b* = SH3 and Multiple Ankyrin Repeat Domains 3B; mg/day = milligrams per day.

**Table 13:** Clinical Studies Evaluating L-Carnitine and ALCAR in Neurodevelopmental Disorders.

Reference	Population (N)	Study Type	Dose & Duration	Primary Endpoints	Key Results	Study Limitations/Risk of Bias/Notes
Geier et al., 2011 [189]	Children with ASD (N = 30)	RCT (Double-blind, placebo-controlled)	50 mg/kg/day for 3 months	Autism severity scores (CARS), clinical behavioral metrics	Improvement in CARS total scores; positive outcomes directly correlated with increased serum carnitine levels.	Small sample size (N = 15 per arm); single-center pilot design; short follow-up window to assess long-term developmental maintenance.
Fahmy et al., 2013 [190]	Children with ASD (N = 30)	Randomized, double-blind, placebo-controlled clinical trial	100 mg/kg/day for 6 months	Childhood Autism Rating Scale (CARS) scores, cognitive and communication subscales	Post-treatment improvements in communication, cognition, and overall CARS total severity scores.	Lack of a parallel placebo control group; small cohort footprint; potential open-label investigator and parental expectation bias.
Adams et al., 2018 [193]	Children and adults with ASD (N = 117 completed)	RCT (Single-blind, randomized controlled trial)	Comprehensive multi-supplement protocol (including carnitine) for 12 months	Overall autism symptoms, non-verbal cognition, adaptive functioning	Improvement in adaptive behavior, non-verbal IQ, and systemic metabolic parameter matrix vs. non-treated controls.	Complex multi-supplement integrative matrix design; impossible to isolate or quantify the independent therapeutic effect of carnitine alone.
Shakibaei & Jelvani, 2023 [198]	Children and adolescents with ASD (N = 50; 25 per group)	Randomized, double-blind, placebo-controlled adjunctive trial	L-carnitine + risperidone vs. placebo + risperidone; 8 weeks	Aberrant Behavior Checklist (ABC), assessed at baseline and weeks 4 and 8	Improvement in total ABC score and lethargy/social-isolation subscale with adjunctive L-carnitine compared with placebo; within-group reductions were also reported in several ABC subscales.	Relatively small sample; short treatment duration; adjunctive design prevents isolation of the independent effect of L-carnitine; findings require confirmation in larger trials.
Nasiri et al., 2024 [199]	Children with ASD (N = 68 randomized; N = 60 completed)	RCT (Double-blind, placebo-controlled adjunctive trial)	L-carnitine 150 mg/day + risperidone for 10 weeks	Aberrant Behavior Checklist (ABC) subscales (Irritability and Hyperactivity)	Reductions in irritability (*p* = 0.033) and hyperactivity (*p* < 0.001) with L-carnitine + risperidone versus placebo + risperidone; no significant between-group differences in lethargy, stereotypic behavior, or inappropriate speech.	Co-administration protocol with atypical antipsychotics; relatively short clinical treatment duration; single-region cohort footprint; findings considered preliminary by the authors.

Abbreviations: ABC, Aberrant Behavior Checklist; ASD, autism spectrum disorder; CARS, Childhood Autism Rating Scale; RCT, randomized controlled trial.

**Table 14:** Secondary Research and Mechanistic Evidence for L-Carnitine and ALCAR.

Reference	Scope/Model/Dataset	Study Type	Dose & Duration	Primary Endpoints	Key Neurobiological Findings	Study Limitations/Risk of Bias/Notes
Rueda et al., 2015 [191]	Fragile X Syndrome (2 RCTs; total pooled N = 81)	Cochrane Systematic Review	Variable Acetyl-L-carnitine (ALCAR) protocols	Hyperactivity, inappropriate behavior scales, vocabulary scores	Provided limited, low-quality evidence; individual trials suggested selective baseline improvements in hyperactivity and ADHD-like symptoms.	Small number of included primary trials; high risk of performance and detection bias; narrow syndromic focus.
Ferreira & McKenna, 2017 [192]	Central nervous system pathologies	Mechanistic Review	Narrative data synthesis	Mitochondrial bioenergetics, neurotransmitter pathways	Documented broad neuroprotective, antioxidant, and anti-apoptotic profiles of ALCAR; emphasizes role in acetylcholine synthesis and myelination.	Qualitative synthesis lacking original clinical or primary quantitative data; general mechanistic overview.
Demarquoy et al., 2019 [194]	ASD and metabolic dysfunction	Biochemical Review	Narrative metabolic analysis	Fatty acid transport, beta-oxidation metrics	Linked systemic acylcarnitine abnormalities and reduced baseline carnitine levels to ASD pathophysiology and mitochondrial stress.	Narrative overview; focuses heavily on metabolic biomarkers without clinical trial intervention testing.
Malaguarnera et al., 2019 [195]	ASD and Intellectual Disability (ID)	Comprehensive Review	Literature synthesis parameters	Cognition subscales, language skills, adaptive behavior	Reported potential benefits of carnitine administration on selected behavioral, cognitive, and adaptive outcomes across the reviewed literature.	High methodological heterogeneity among reviewed primary protocols; includes both idiopathic and syndromic cohorts.
Kępka et al., 2021 [196]	Metabolic profiling in ASD populations	Translational Review	Biomarker synthesis data	Serum/plasma carnitine, dried blood spot acylcarnitines	Highlighted alterations in carnitine/acylcarnitine profiles and discussed their potential relevance for metabolic characterization; clinical utility remains to be established.	Exclusively biomarker-focused; clinical application thresholds and targeted therapeutic monitoring criteria remain unstandardized.
Nabi et al., 2023 [197]	Mitochondrial pathology in ASD	Narrative Literature Review	Mitochondrial-targeted agents	Oxidative stress pathways, respiratory chain function	Identified carnitine and ALCAR as potential metabolic interventions targeting oxidative stress and mitochondrial dysfunction	Narrative synthesis; lacks systematic statistical pooling or formal quality/bias assessment of primary sources.
Zahedi et al., 2023 [201]	VPA-induced model of Autism (Rats, N = 40)	Preclinical (Animal study)	Chronic ALCAR treatment protocols	Neuroinflammatory cascades, short-chain fatty acids (SCFAs)	Mitigated systemic and cortical oxidative stress and improved social-interaction measures and locomotor asymmetry.	Preclinical rodent model framework; inherent translational gap when mapping to complex human pediatric neurodevelopment.
Zahedi et al., 2024 [202]	Prenatal valproic acid (VPA)-induced rat model of autism-like phenotypes	Preclinical (Animal study)	ALCAR 300 mg/kg/day, p.o., postnatal days 21–49	Oxidative stress markers, social interaction tests	Attenuated autism-like behavioral and learning deficits; reduced hippocampal oxidative stress; improved parvalbumin-positive interneuron alterations; and modulated mitochondrial membrane potential and mitochondrial biogenesis.	Animal model constraints; short intervention window; lacks long-term developmental tracking into adulthood.
Persico et al., 2025 [200]	ASD and neurodevelopmental conditions	Systematic Review	Multi-nutraceutical frameworks	Clinical safety, metabolic and behavioral phenotypes	Identified L-carnitine among interventions with potentially favorable controlled clinical evidence in pediatric ASD, while emphasizing the limited evidence base.	Limited number and size of available controlled trials; heterogeneous interventions and outcomes; evidence remains insufficient for definitive conclusions regarding efficacy.

Abbreviations: ALCAR, acetyl-L-carnitine; ASD, autism spectrum disorder; ID, intellectual disability; SCFAs, short-chain fatty acids; VPA, valproic acid.

**Table 15:** Clinical Studies Evaluating Resveratrol in Neurodevelopmental Disorders.

Reference	Population (N)	Study Type	Dose & Duration	Primary Endpoints	Key Results	Study Limitations/Risk of Bias/Notes
Hendouei et al., 2020 [229]	Children with ASD (N = 62)	RCT (Double-blind, placebo-controlled trial)	500 mg/day (250 mg twice daily) for 10 weeks (Adjunctive to Risperidone)	Aberrant Behavior Checklist (ABC) subscales, targeting Irritability	No significant between-group differences in irritability, lethargy/social withdrawal, stereotypic behavior, or inappropriate speech. A greater reduction in hyperactivity/non-compliance was observed with adjunctive resveratrol compared with placebo.	Adjunctive evaluation framework (concomitant antipsychotic use limits evaluation of pure resveratrol effects); lack of long-term follow-up data.
Marchezan et al., 2022 [230]	Children with ASD (N = 5)	Prospective Open-Label Pilot Clinical Trial	200 mg/day for 90 days (Trans-resveratrol supplementation)	ABC total scores, irritability subscales, circulating microRNA profiles	Reductions in ABC total and irritability scores were observed after treatment; circulating miR-195-5p levels increased.	Extremely small sample (N = 5); open-label design without placebo control; findings are exploratory and do not establish efficacy.

Abbreviations: ASD = Autism Spectrum Disorder; RCT = Randomized Controlled Trial; ABC = Aberrant Behavior Checklist; miR-195-5p = microRNA-195-5p; N = number of participants; mg/day = milligrams per day.

**Table 16:** Secondary Research and Preclinical Evidence for Resveratrol.

Reference	Scope/Model/Population	Study Type	Dose & Duration	Primary Endpoints	Key Neurobiological Findings	Study Limitations/Risk of Bias/Notes
Bambini-Junior et al., 2014 [224]	VPA-induced model of Autism (Rats, N = 24)	Preclinical (Animal study)	Prenatal administration (3.6 mg/kg maternal injection)	Social interaction profiles, sociability markers	Prenatal treatment successfully prevented post-natal social interaction deficits and normalized social preference behaviors.	Preclinical rodent design; inherent translational limits when mapping to complex human clinical neurodevelopment.
Bhandari et al., 2017 [225]	Propionic acid (PPA)-induced Autism (Rats, N = 32)	Preclinical (Animal study)	5, 10, and 20 mg/kg/day for 28 days	Oxidative stress indices, TNF-α and MMP-9 levels, behaviors	Dose-dependently reduced oxidative-nitrosative stress; restored mitochondrial complex functions; suppressed elevated TNF-α and MMP-9.	Acute chemical induction model; short observation window; focus is primarily mechanistic rather than longitudinal.
Xie et al., 2018 [226]	Progestin-induced model of Autism (Rats, N = 30)	Preclinical (Animal study)	Prenatal administration (20 mg/kg/day maternal oral gavage)	ERβ signaling pathways, histone demethylation, behaviors	Ameliorated autism-like behaviors through the direct activation of estrogen receptorβ (ERβ) and downstream epigenetic modifications.	Highly specific, hormone-driven syndromic model; narrow pathophysiology; general translation to idiopathic ASD is limited.
Fontes-Dutra et al., 2018 [227]	VPA-induced model of Autism (Rats, N = 24)	Preclinical (Animal study)	Maternal dietary enrichment (3.6 mg/kg during gestation)	Sensory gating mechanisms, cortical neuroplasticity	Prevented atypical sensory processing and primary behavioral abnormalities; protected cortical structural plasticity.	Rodent sensory framework; lacks cross-species verification; difficult to extrapolate directly to complex human sensory phenotypes.
Hidema et al., 2020 [228]	Oxytocin receptor-knockout Mice (N = 20)	Preclinical (Animal study)	Acute systemic administration (20 mg/kg single dose)	Oxytocinergic signaling, targeted social behavior scales	Acute administration bypassed receptor deficiencies to significantly improve core social interaction deficits.	Genetically restricted knockout strain; evaluates acute behavioral response rather than long-term steady-state developmental recovery.
Malaguarnera et al., 2020 [231]	ASD and related developmental conditions	Narrative Literature Review	Systemic review parameters	Antioxidant pathways, cytokine networks, mitochondrial health	Synthesized multi-target mechanisms highlighting resveratrol’s capacity to optimize neuroimmune balance and mitochondrial protection.	N/A (Narrative Review); descriptive synthesis that lacks systematic meta-analytical statistical pooling or formal risk of bias scoring.
Nikfarjam et al., 2026 [232]	Experimental and clinical literature concerning resveratrol in ASD	Comprehensive Systematic Review	Meta-synthesis parameter ranges, Heterogeneous across included studies	Behavioral and neurodevelopmental outcomes; oxidative, inflammatory, mitochondrial, epigenetic, and neurotransmitter-related mechanisms	Identified potential effects of resveratrol across several behavioral and neurobiological pathways relevant to ASD, while supporting further investigation of its preventive and therapeutic potential.	N/A (Systematic Review); qualitative summary restricted by high methodological heterogeneity among the primary reviewed datasets. Evidence base includes substantial preclinical evidence and limited human clinical data; literature search conducted through December 2024.

Abbreviations: VPA = Valproic Acid; PPA = Propionic Acid; TNF-α = Tumor Necrosis Factor-alpha; MMP-9 = Matrix Metalloproteinase-9; ERβ = Estrogen Receptor-beta; ASD = Autism Spectrum Disorder; N = number of subjects; N/A = Not Applicable; mg/kg = milligrams per kilogram; mg/kg/day = milligrams per kilogram per day.

**Table 17:** Clinical Studies Evaluating Phosphatidylserine (PS) in Neurodevelopmental Disorders.

Reference	Population (N)	Study Type	Dose & Duration	Primary Endpoints	Key Results/Findings	Study Limitations/Risk of Bias/Notes
Manor et al., 2012 [243]	Children with ADHD (N = 200)	Double-Blind RCT	*PS-Omega-3* (300 mg PS/day + *EPA/DHA*) for 15 weeks; followed by a 15-week open-label extension	ADHD core symptoms, impulsivity scores, emotional regulation scales	Limited between-group effects were observed during the double-blind phase, including improvement in parent-rated restless/impulsive behavior; exploratory subgroup analyses suggested greater responses in children with more pronounced hyperactive/impulsive and emotional/behavioral features.	Combined PS-omega-3 formulation prevents attribution of effects specifically to PS; several outcomes were negative and subgroup findings should be interpreted cautiously.
Hirayama et al., 2014 [244]	Children with ADHD (N = 36)	Double-Blind RCT (Placebo-controlled)	Soy-derived phosphatidylserine 200 mg/day for 2 months	Visual attention performance, short-term auditory memory, impulsivity indices	Improvements in ADHD symptom ratings, short-term auditory memory, and selected measures of inattention/impulsivity; however, reporting of between-group comparisons was incomplete for some outcomes.	Relatively small sample footprint (N = 36); single-center recruitment; short intervention window to analyze long-term cognitive retention.
Rheims et al., 2024 [246]	Children and adolescents aged 6–15 years with epilepsy and comorbid ADHD (N = 74 randomized; PS-Omega-3 n = 44, placebo n = 30)	Multicenter RCT (Placebo-controlled)	Phosphatidylserine + Omega-3 complex for 12 weeks	Global ADHD symptom severity, executive functioning indices	No significant between-group difference in the primary inattention outcome at 12 weeks; no significant differences were observed in the other ADHD-related outcomes.	The trial was terminated early because of insufficient recruitment and remained underpowered; the intervention combined PS with omega-3 PUFAs, preventing assessment of the independent effect of PS.
Shen et al., 2026 [247]	Children with ADHD (N = 56)	Randomized, open-label, controlled trial	PS vs. atomoxetine for 3 months	Core ADHD symptoms and associated behavioral outcomes	PS did not significantly improve core ADHD symptoms; however, within-group reductions in internalizing and externalizing behavioral problems, particularly aggressive behavior, were reported. Atomoxetine showed significant improvement in ADHD symptom measures.	Open-label design; active comparator rather than placebo; relatively small sample; findings do not establish equivalence or non-inferiority and require confirmation in blinded placebo-controlled trials.

Abbreviation: ADHD = Attention-Deficit/Hyperactivity Disorder; PS = Phosphatidylserine; RCT = Randomized Controlled Trial; EPA = Eicosapentaenoic Acid; DHA = Docosahexaenoic Acid; PUFA = Polyunsaturated Fatty Acids; N = number of participants; n = Number of participants in a subgroup; mg/day = milligrams per day.

**Table 18:** Secondary Research and Mechanistic Evidence for Phosphatidylserine (PS).

Reference	Scope/Model/Dataset	Study Type	Dose (Typical)	Outcomes/Pathways	Key Results	Study Limitations/Risk of Bias/Notes
Bruton et al., 2021 [245]	Pediatric ADHD (Pooled clinical trials)	Systematic Review & Meta-analysis	200–300 mg/day of standardized PS	Inattention severity index, hyperactivity subscales	Found a statistically significant clinical reduction in inattention scores; noted that efficacy data for global hyperactivity mitigation remain less consistent.	Substantial methodological heterogeneity across the primary included trials; variable duration parameters and diagnostic criteria.
Ma et al., 2022 [242]	Central nervous system (CNS) pathologies	Mechanistic Review	Phosphatidylserine-mediated pathways	Neuroinflammation cascades, microglial activation, synaptic signaling	Documented broad neuroprotective, anti-apoptotic, and anti-inflammatory roles; highlighted active regulatory modulation of neuroimmune networks.	Qualitative narrative synthesis; explicitly lacks original primary clinical cohort data or systematic meta-analytical pooling.

Abbreviations: ADHD = Attention-Deficit/Hyperactivity Disorder; PS = Phosphatidylserine; CNS = Central Nervous System; mg/day = milligrams per day.

**Table 19:** Clinical Studies Evaluating Coenzyme Q10 in Neurodevelopmental Disorders.

Reference	Population (N)	Study Type	Dose & Duration	Primary Endpoints	Key Results	Study Limitations/Risk of Bias/Notes
Miles et al., 2006 [275]	Children with Down Syndrome (N = 16 completed)	Open-label Pharmacokinetic & Safety Study	1 mg/kg/day for 1 month, followed by 10 mg/kg/day for 2 months	Plasma CoQ10 absorption kinetics, systemic tolerability thresholds	Documented excellent oral bioavailability and clinical safety; successfully elevated plasma concentrations without adverse events.	Small sample size; open-label design lacking a placebo group; limited tracking of long-term functional efficacy endpoints.
Adams et al., 2011 [276]	Children and adults with ASD (N = 141 enrolled)	RCT (Double-blind, placebo-controlled trial)	Comprehensive multivitamin/mineral matrix (including CoQ10) for 3 months	Global autism symptoms, metabolic profiles, hepatic detox parameters	Statistically significant improvements in overall behavioral and developmental outcomes vs. the parallel placebo cohort.	Complex integrative multi-supplement approach; impossible to isolate the specific, independent therapeutic weight of CoQ10 alone.
Tiano et al., 2011 [273]	Children with Down syndrome (N = 30)	Double-blind, randomized, placebo-controlled clinical trial	CoQ10 supplementation (4 mg/kg/day) for 6 months	Oxidative DNA damage and circulating CoQ10 concentrationstterns	CoQ10 supplementation increased circulating CoQ10 levels and showed limited protective effects against oxidative DNA damage; no significant overall reduction in DNA oxidation was observed at the patient level.	Small sample size (N = 30); short treatment duration; biomarker-based outcomes without assessment of cognitive, adaptive, or behavioral improvements; limited generalizability to ASD and other neurodevelopmental disorders.
Mousavinejad et al., 2018 [280]	Children with ASD (N = 90)	RCT (Double-blind, placebo-controlled trial)	30–60 mg/day for 100 days of standardized CoQ10	Systemic oxidative stress markers, CARS scores, GI symptoms	Reductions in oxidative-stress biomarkers were reported, together with changes in selected clinical measures, including sleep and gastrointestinal symptoms; associations with CARS scores were also examined	Single-center recruitment matrix; relatively short post-treatment follow-up window; heavy focus on baseline biochemical correlations.
Cucinotta et al., 2022 [272]	Patients with neurodevelopmental disorders (N = 59): ASD (n = 17), ASD + ID (n = 19), ID/global developmental delay (n = 15), ADHD (n = 3), and Phelan–McDermid syndrome with ID (n = 5)	Retrospective Chart Review	Ubiquinol combined with Vitamins B and E for 3 months	Daily adaptive functioning scores, oxidative stress balance	Clinical improvement was retrospectively documented in several diagnostic groups, including ID/GDD and ASD with comorbid ID; treatment was generally well tolerated.	Retrospective uncontrolled chart review; heterogeneous diagnostic groups; combined ubiquinol + vitamin E + B-complex intervention prevents attribution of observed effects specifically to CoQ10; no placebo comparator.

Abbreviations: CoQ10 = Coenzyme Q10; ASD = Autism Spectrum Disorder; RCT = Randomized Controlled Trial; CARS = Childhood Autism Rating Scale; GI = Gastrointestinal; ID = Intellectual Disability; ADHD = Attention-Deficit/Hyperactivity Disorder; GDD = Global Developmental Delay; N = number of participants; n = number of participants in a subgroup; mg/day = milligrams per day; mg/kg/day = milligrams per kilogram per day.

**Table 20:** Secondary Research and Mechanistic Evidence for Coenzyme Q10.

Reference	Scope/Model/Dataset	Study Type	Dose (Typical)	Outcomes Evaluated	Key Synthesized Results	Study Limitations/Risk of Bias/Notes
El-Ansary et al., 2016 [277]	Young male Western albino rats (rodent model of autism induced via propionic acid)	Pre-clinical, experimental in vivo controlled laboratory study	Combined antioxidant and mitochondrial-supportive therapy (including CoQ10 and melatonin)	Fatty acid profiles, lipid peroxidation markers, and redox status in brain tissue	Demonstrated significant improvements in overall brain fatty acid balance and a marked reduction in neurotoxic lipid damage.	Focus is strictly biochemical and mechanistic; lacks direct correlation with longitudinal behavioral randomized controlled trial (RCT) metrics.
Mousa-vinejad et al., 2016 [276]	Mitochondrial pathways in autism	Pathophysiological Literature Review	N/A (Review framework)	Mitochondrial respiration efficiency, oxidative stress	Reviewed evidence linking mitochondrial dysfunction, oxidative stress, and altered CoQ10 status in subsets of individuals with ASD, supporting investigation of mitochondrial-targeted interventions	N/A (Narrative Review); entirely descriptive synthesis that lacks quantitative meta-analytical pooling or statistical risk grading.
Demarquoy et al., 2019 [194]	Mitochondrial subgroups in neurodevelopment	Metabolic Literature Review	N/A (Review framework)	Fatty acid beta-oxidation, mitochondrial respiratory chain	Provided integrative metabolic evidence supporting the hypothesis that mitochondrial and metabolic alterations may characterize specific ASD subgroups and may inform investigation of targeted metabolic interventions.	N/A (Metabolic Review); qualitative overview with a narrow biomarker focus; lacks original primary clinical datasets.
Burke et al., 2021 [279]	Oxidative pathways in ASD/ID populations	Comprehensive Narrative Review	N/A (Review framework)	Systemic oxidative stress cascades	Discussed CoQ10 among antioxidant interventions with potential biological relevance to oxidative-stress pathways in neurodevelopmental disorders.	N/A (Narrative Review); narrative synthesis format lacking rigorous systematic search criteria or RCT-level clinical evidence grading.
Bagheri et al., 2023 [281]	CoQ10 in neurological disorders	Narrative Review	N/A	Mitochondrial function, oxidative stress, neuroprotection	Reviews antioxidant and mitochondrial mechanisms underlying the potential neuroprotective actions of CoQ10 across neurological disorders.	Broad neurological focus; predominantly neurodegenerative rather than neurodevelopmental evidence; limited direct relevance to ASD/ID populations.
Mantle et al., 2024 [282]	CoQ10 supplementation in neonates, infants, and children	Narrative/Clinical Review	Variable across pediatric conditions	Safety, tolerability, clinical efficacy of CoQ10	Pediatric evidence indicates generally favorable tolerability and examines supplementation across several childhood disorders, including Down syndrome and ADHD.	Highly heterogeneous disorders and dosing regimens; not specifically focused on ASD or ID.
Ramírez-Guerrero et al., 2025 [171]	Antioxidant-based pharmacological interventions in ASD	Systematic Review	Variable	ASD symptoms, oxidative stress, antioxidant interventions	Identified CoQ10 among antioxidant interventions studied in ASD; available CoQ10 evidence suggested effects on oxidative-stress biomarkers and selected sleep/GI outcomes, while evidence for core ASD symptoms remained limited.	Heterogeneous interventions and outcome measures; limited number of CoQ10 studies; insufficient evidence to establish CoQ10 efficacy as monotherapy.
Mantle & Hargreaves, 2025 [283]	CoQ10 and blood–brain barrier	Narrative/Mechanistic Review	N/A	BBB penetration, mitochondrial function, neurological effects	Reviews the biological rationale for CoQ10 supplementation but highlights uncertainty concerning the extent to which orally administered CoQ10 reaches brain tissue.	Not ASD-specific; primarily mechanistic and neurological evidence rather than neurodevelopmental clinical trials.
Scenna et al., 2026 [14]	Natural antioxidants and mitochondrial-targeted interventions in ASD	Review	CoQ10 30–60 mg/day in cited pediatric study	Oxidative stress, mitochondrial dysfunction, ASD-related outcomes	Reviews CoQ10 as a mitochondrial/antioxidant intervention in ASD and discusses pediatric clinical evidence showing modulation of oxidative-stress markers.	Clinical evidence remains sparse; mechanistic rationale is substantially stronger than evidence for improvement of core ASD symptoms.

Abbreviation: CoQ10 = Coenzyme Q10; ASD = Autism Spectrum Disorder; ID = Intellectual Disability; RCT = Randomized Controlled Trial; BBB = Blood–Brain Barrier; ADHD = Attention-Deficit/Hyperactivity Disorder; GI = Gastrointestinal; N/A = Not Applicable; mg/day = milligrams per day.

**Table 21:** Clinical Studies Evaluating Omega-3 Fatty Acids in ASD and ADHD.

Reference	Population (N)	Study Type	Dose & Duration	Primary Endpoints	Key Results	Study Limitations/Risk of Bias/Notes
Bent et al., 2011 [304]	Children with ASD (N = 27 randomized)	Pilot Randomized Controlled Trial (RCT)	1.3 g/day of omega-3 PUFAs for 12 weeks	Hyperactivity subscales (ABC-H), safety parameters, plasma fatty acids	Some changes in hyperactivity-related measures were observed; however, the small sample and lack of statistically robust between-group effects precluded definitive conclusions regarding efficacy.	Small pilot sample (N = 27), limiting statistical power; short intervention duration and exploratory nature of the study.
Bent et al., 2014 [305]	Children with ASD (N = 57 randomized)	Internet-based, Double-Blind RCT (Placebo-controlled)	1.3 g/day of omega-3 PUFAs for 6 weeks	Parent- and teacher-rated hyperactivity scores (ABC-H)	Omega-3 supplementation was generally well tolerated; hyperactivity decreased numerically more in the omega-3 group, but the between-group difference was not statistically significant.	High attrition risks inherent to fully remote models; Short intervention duration (6 weeks), limiting assessment of sustained behavioral effects
Doaei et al., 2021 [308]	Children with ASD (N = 54 completed)	Double-Blind RCT (Placebo-controlled)	1000 mg/day (1 g/day) of omega-3 for 8 weeks	Clinical autism symptoms (GARS scores), social communication, metabolic biomarkers	Improvements in stereotyped behaviors, social communication, and total GARS score compared with placebo; no significant improvement in the social interaction subscale.	Moderate sample size; single-center execution; lack of long-term post-treatment follow-up to check symptom retention.

Abbreviation: ASD = Autism Spectrum Disorder; RCT = Randomized Controlled Trial; PUFAs = Polyunsaturated Fatty Acids; ABC-H = Aberrant Behavior Checklist–Hyperactivity Subscale; GARS = Gilliam Autism Rating Scale; N = number of participants; g/day = grams per day; mg/day = milligrams per day.

**Table 22:** Secondary Research and Translational Evidence for Omega-3 Fatty Acids.

Reference	Scope/Dataset/Population	Study Type	Dose/Formulations Evaluated	Core Outcomes Evaluated	Key Synthesized Results	Study Limitations/Risk of Bias/Notes
Bent et al., 2009 [302]	Pediatric ASD populations	Systematic Literature Review	Standardized Omega-*3* frameworks	Core autism features, hyperactivity indices, safety	Concluded that baseline data showed insufficient evidence to verify absolute efficacy; noted positive directional trends for hyperactivity and stereotypy mitigation.	Qualitative review framework; limited by the systematic scarcity and small sample sizes of available primary randomized controlled trials (RCTs) at that time.
James et al., 2011 [303]	Pediatric ASD cohorts	Cochrane Systematic Review	Variable Omega-3 oral protocols	Global behavioral scales, communication indices	Highlighted an overall lack of robust supportive evidence due to high trial heterogeneity; confirmed excellent safety and compound tolerability.	Meta-analytical pooling constrained by highly diverse primary trial methodologies; strongly emphasized the imperative need for large-scale RCTs.
Cheng et al., 2017 [306]	Children with ASD (Pooled sample from 6 RCTs)	Systematic Review & Meta-analysis	Standardized Omega-3 regimens	Hyperactivity subscales, lethargy, stereotyped behaviors	Documented modest but statistically significant pooled improvements in hyperactivity, lethargy, and stereotyped behavior domains.	Marked methodological heterogeneity across the primary included trials; small individual study sample sizes limit generalizability.
Horvath et al., 2017 [307]	Pediatric ASD cohorts (Pooled clinical trial datasets)	Systematic Review & Meta-analysis	Variable Omega-3 dosing parameters	Core ASD symptoms, sensory profiles, safety thresholds	Confirmed excellent compound tolerability and long-term clinical safety; noted limited or negligible pooled efficacy on core autism symptoms.	Qualitative narrative synthesis elements; clinical trials reviewed showed high baseline variability in treatment duration and diagnostic scales.
Donovan et al., 2020 [311]	Maternal exposure & pediatric offspring	Systematic Literature Review	N/A (Review framework)	Long-term cognitive development tracking	Reported that maternal supplementation during pregnancy favorably trends toward better cognitive scores and mental processing in offspring.	N/A (Narrative Review); entirely descriptive synthesis; preventive evidence for specific ASD or ADHD behavioral phenotypes remains inconclusive.
de Andrade Wobido et al., 2022 [309]	Children and adolescents with ASD (Pooled clinical datasets)	Systematic Review & Meta-analysis	Combined Omega-3 and Omega-6 matrices	Aberrant Behavior Checklist (ABC) scores	Reported improvements in selected ABC behavioral outcomes following combined omega-3/omega-6 supplementation.	Analysis limits the capacity to isolate pure omega-3 efficacy due to combined PUFA approach; outcomes vary significantly by fatty acid ratios.
Liu et al., 2023 [310]	Pediatric ADHD populations (Pooled multi-center datasets)	Systematic Review & Meta-analysis	Long-term regimens (Higher EPA vs. DHA content)	Attention parameters, executive function, hyperactivity	Reported potential effects on selected behavioral and cognitive outcomes, with some findings suggesting that EPA-predominant formulations may be associated with greater effects in specific domains; effects on overall core ADHD symptoms remained inconsistent.	Substantial heterogeneity in EPA/DHA composition, treatment duration, baseline fatty-acid status, and outcome measures; evidence does not establish an optimal formulation or consistent efficacy for core ADHD symptoms.
Sumra et al., 2025 [312]	Children with ASD aged 2–18 years; 11 studies included	Systematic Review	Heterogeneous EPA/DHA formulations and intervention durations	Cognitive flexibility, executive functioning, working memory	Reported potentially favorable findings for selected cognitive domains, including memory, attention, and executive functioning; however, no conclusive overall benefit was established.	Considerable heterogeneity in study designs, doses, intervention duration, and outcome assessment; limited statistical power of available studies; no quantitative meta-analysis performed.
Jia et al., 2025 [313]	Individuals with ASD; 11 studies, N = 416	Systematic Review & Meta-analysis	Variable omega-3 formulations and treatment protocols	Overall ASD symptoms and behavioral domains including hyperactivity, stereotypy, communication, and emotional difficulties	Omega-3 supplementation produced a small, non-significant overall effect, with no statistically significant benefit across the evaluated symptom subdomains compared with control conditions.	Considerable heterogeneity in treatment protocols and participant characteristics; available evidence does not establish clinically meaningful efficacy for overall or core ASD symptoms.
Abbasi et al., 2025 [314]	Individuals with ASD; 7 published meta-analyses included	Umbrella Review of Meta-analyses	Variable omega-3 formulations, doses, and treatment durations	Core and non-core ASD manifestations, including hyperactivity, irritability, stereotypy, social withdrawal/lethargy, and cluttering speech	Overall effects were outcome- and subgroup-dependent. Significant reductions in hyperactivity and cluttering speech were identified in participants ≤8 years in subgroup analyses, whereas other ASD outcomes did not show consistent benefit; ≤1000 mg/day was associated with increased stereotypy/restricted and repetitive behaviors in one analysis.	Findings derive from previously published meta-analyses and heterogeneous primary trials; significant effects were largely subgroup-specific and should not be interpreted as evidence of broad efficacy for core ASD symptoms.
Fu et al., 2026 [315]	Children and adolescents with ADHD; 7 RCTs providing 10 analytical estimates	Systematic Review and Biomarker-Stratified Meta-analysis	Heterogeneous EPA/DHA formulations; intervention durations generally 12–24 weeks	ADHD-related behavioral, cognitive, and academic outcomes according to baseline omega-3 biomarker status	Overall analysis showed a small effect (SMD 0.21), but sensitivity analysis using one estimate per parent trial was non-significant. Larger effects were observed in study-defined low-baseline omega-3 subgroups (SMD 0.52), whereas normal/high or unstratified groups showed no significant effect.	Only 7 RCTs; heterogeneous biomarker matrices and definitions of low baseline status; several subgroup analyses were post hoc. Findings are preliminary and hypothesis-generating and do not yet support routine biomarker-guided supplementation.

Abbreviations: ASD = Autism Spectrum Disorder; ADHD = Attention-Deficit/Hyperactivity Disorder; RCT = Randomized Controlled Trial; ABC = Aberrant Behavior Checklist; PUFA = Polyunsaturated Fatty Acids; EPA = Eicosapentaenoic Acid; DHA = Docosahexaenoic Acid; SMD = Standardized Mean Difference; N = number of participants; N/A = Not Applicable; mg/day = milligrams per day.

**Table 23:** Clinical Studies Evaluating Probiotics in Neurodevelopmental Disorders.

Reference	Population (N)	Study Type	Strain/Intervention & Duration	Core Outcomes Evaluated	Key Clinical Results	Study Limitations/Risk of Bias/Notes
Pärtty et al., 2015 [352]	Children followed from infancy to 13 years (N = 75 at follow-up; LGG n = 40, placebo n = 35)	Randomized placebo-controlled early-life intervention with long-term follow-up	*Lactobacillus rhamnosus* GG (1 × 10^10^ CFU/day), administered during the first 6 months of life	ADHD and Asperger syndrome diagnoses at 13 years; gut microbiota composition	ADHD and/or Asperger syndrome was diagnosed in 6/35 children in the placebo group and 0/40 in the LGG group (*p* = 0.008), suggesting a possible association between early-life probiotic exposure and later neurodevelopmental outcomes. |	Small follow-up sample and very low number of diagnostic events; neurodevelopmental outcomes were assessed many years after the intervention; findings are hypothesis-generating and do not establish a preventive effect of LGG.
Kang et al., 2017 [347]	Children with ASD (N = 18)	Open-Label Clinical Trial	Microbiota Transfer Therapy (MTT) for 7–8 weeks	Gastrointestinal symptoms, core ASD behavioral phenotypes	Reported sustained improvements in gastrointestinal symptoms and ASD-related behavioral measures during follow-up.	Landmark microbiome study but constrained by a small sample size (N = 18); open-label baseline configuration lacking a parallel placebo control group.
Shaaban et al., 2018 [348]	Children with ASD (N = 30)	Prospective Open-Label Clinical Trial	Multi-strain probiotic formulation for 3 months	Gastrointestinal parameter tracking, behavioral rating indices	Reported improvements in gastrointestinal symptoms and behavioral measures following probiotic supplementation.	Small sample size (N = 30); open-label design lacking placebo control validation; single-center exploratory framework.
Liu et al., 2019 [371]	Children and adolescents with ASD (N = 71 completed)	Randomized Controlled Trial (Double-blind)	*Lactobacillus plantarum* PS128 for 4 weeks	Hyperactivity subscales, opposition/defiance indices	PS128 was associated with improvement in opposition/defiance behaviors; significant improvement in the SNAP-IV total score compared with placebo was observed particularly in younger children aged 7–12 years.	Short intervention duration (4 weeks); male-only sample; several findings were age- or outcome-specific and should not be interpreted as evidence of broad improvement in core ASD symptoms.
Sanctuary et al., 2019 [350]	Children with ASD and comorbid GI symptoms (N = 10)	Pilot Intervention Study	Probiotic matrix combined with bovine colostrum	Gastrointestinal transit function, behavioral parameters	Reported improvements in selected gastrointestinal and behavioral measures following the combined intervention.	Extremely small sample size N = 10; combined matrix formulation makes it impossible to isolate the independent therapeutic effect of the probiotic strain.
Arnold et al., 2019 [351]	Children with ASD, anxiety, and GI symptoms (N = 13 randomized; N = 10 completed)	Randomized placebo-controlled crossover pilot trial	VISBIOME multi-strain probiotic, 8 weeks probiotic and 8 weeks placebo separated by a 3-week washout	GI-related quality of life, anxiety, parent-selected target symptoms, gut microbiota	Primary GI quality-of-life and anxiety outcomes did not differ significantly between probiotic and placebo; a parent-selected GI target symptom improved significantly during probiotic treatment.	Very small pilot sample; crossover design with evidence of carryover effects; insufficient statistical power for definitive efficacy conclusions.
Li et al., 2025 [354]	Children with ASD (N = 50)	Randomized Clinical Trial	*Bifidobacterium longum* subsp. *longum* CCFM1077	CARS symptom severity scores, anxiety scales	Achieved significant global symptom reductions driven by a targeted drop in baseline CARS scores and anxiety phenotypes.	Evaluation of a novel single strain; long-term symptom retention data are lacking; potential indicator for precision psychobiotic applications.
Rojo-Marticella et al., 2025 [364]	Children and adolescents with ADHD (N = 38) or ASD (N = 42), aged 5–16 years	Randomized, controlled probiotic intervention trial	*Lactiplantibacillus plantarum* + *Levilactobacillus brevis* for 12 weeks	Behavioral regulation parameters, dual-diagnosis tracking	Improvements in selected symptom measures were observed, including hyperactivity–impulsivity in younger children with ASD; effects were outcome- and subgroup-specific rather than evidence of broad improvement across ASD or ADHD symptoms.	Pilot-scale study; diagnostic groups analyzed separately; several findings were subgroup-specific; limited sample size and short intervention duration.

Abbreviations: ASD = Autism Spectrum Disorder; ADHD = Attention-Deficit/Hyperactivity Disorder; LGG = Lactobacillus rhamnosus GG; CFU = Colony-Forming Units; MTT = Microbiota Transfer Therapy; GI = Gastrointestinal; SNAP-IV = Swanson, Nolan, and Pelham Rating Scale-IV; CARS = Childhood Autism Rating Scale; N = number of participants; n = number of participants in a subgroup.

**Table 24:** Secondary Research and Mechanistic Evidence for Probiotics.

Reference	Scope/Pooled Dataset/Model	Study Type	Formulations & Interventions Evaluated	Core Outcomes Evaluated	Key Synthesized Results	Study Limitations/Risk of Bias/Notes
Ng et al., 2019 [356]	Pediatric ASD cohorts	Systematic Review	Variable probiotic regimens	Gastrointestinal pathways, core behavioral domains	Provided mixed and highly variable evidence across cohorts; confirmed general safety and tolerability of targeted supplementation.	Marked by extensive methodological heterogeneity across the included primary literature; small sample footprints in individual cohorts.
Wang et al., 2020 [353]	Children with ASD vs. healthy controls	Translational Microbiome Analysis	*Bifidobacterium longum* compositional tracking	Gut microbiota architecture, short-chain fatty acids (SCFAs)	Demonstrated that a targeted increase in *Bifidobacterium* abundance directly correlates with a significant reduction in pathogenic *Clostridium* populations.	Strictly observational and translational baseline design; establishes physiological biomarker rationale without implementing an interventional trial.
Wong et al., 2021 [33]	Neurodevelopmental disorders	Neurological Literature Review	N/A (Review framework)	Microbiota–gut–brain axis signaling networks	Reviewed biological mechanisms supporting the plausibility of gut-directed therapeutic strategies	N/A (Narrative Review); entirely descriptive, qualitative overview; lacks meta-analytical dataset pooling or quantitative risk grading.
Sgritta et al., 2019 [372]	Genetic, environmental (including VPA), and idiopathic mouse models of ASD-like social deficits	Preclinical animal study	*Lactobacillus reuteri*	Social behavior, vagal signaling, oxytocin-related signaling, VTA synaptic plasticity	*L. reuteri* selectively rescued social deficits across several ASD mouse models, including VPA-exposed mice; effects required intact vagal signaling and oxytocin receptor signaling	Preclinical models; no direct evidence of clinical efficacy in ASD.
Duque et al., 2021 [355]	Fecal microbiota-derived in vitro model from children with ASD	In vitro mechanistic study	*Limosilactobacillus reuteri + Bifidobacterium longum*, *GOS*, and *synbiotic combination*	Gut microbiota composition, microbial metabolic activity, SCFAs, ammonium	Probiotic, prebiotic, and synbiotic interventions modified microbial composition and metabolic activity; prebiotic and synbiotic treatments increased SCFAs and reduced ammonium concentrations	In vitro SHIME^®^ model; no behavioral or clinical outcomes; findings do not establish clinical efficacy.
Song et al., 2022 [357]	Children with ASD (Pooled analysis of 9 controlled trials)	Systematic Review & Meta-analysis	Probiotics and prebiotic matrices	Global ASD clinical severity, gastrointestinal symptoms	Found no statistically significant overall pooled effect on global ASD severity, but noted selective improvements in specific behavioral subscales.	Conflicting and highly variable subgroup findings; high baseline heterogeneity across the primary pooled trial frameworks.
Patel, 2022 [358]	Pediatric neurodevelopmental cohorts	Narrative Systematic Synthesis	Variable commercial probiotic strains	Behavioral adaptation, gastrointestinal distress	Highlighted highly inconsistent clinical evidence across the literature regarding core behavioral symptom mitigation.	Strongly emphasizes a critical lack of cross-study standardization in dosing, cell counts, and primary outcome metrics.
He et al., 2023 [359]	Children with ASD (Pooled data from randomized trials)	Systematic Review & Meta-analysis	Multi-strain probiotic formulations	Objective behavioral symptoms, adaptive functioning	Subgroup analyses suggested potentially greater effects with multi-strain formulations; however, overall effects remained heterogeneous and the finding does not establish superiority of multi-strain over single-strain probiotics.	Pooled analysis remains constrained by varying treatment durations and unequal baseline diagnostic score severities.
Rahim et al., 2023 [360]	Neurodevelopmental and intellectual disabilities	Umbrella Review & Meta-analysis	Probiotics, prebiotics, and synbiotic protocols	Gastrointestinal regulation, behavioral symptom clusters	Suggested moderate, statistically significant beneficial effects on target gastrointestinal distress parameters and selective behavioral outcomes.	Exceptionally broad evaluation scope; high inter-study heterogeneity due to the pooling of distinct pre-, pro-, and synbiotic formulations.
Lewandowska-Pietruszka et al., 2023 [361]	Pediatric neurodevelopmental syndromes	Comprehensive Literature Review	N/A (Review framework)	Intestinal dysbiosis, systemic inflammatory markers	Reported recurrent alterations in the relative abundance of several bacterial taxa, including *Lactobacillus* and *Bifidobacterium*, across ASD studies, while emphasizing substantial inter-study variability.	Qualitative narrative overview format; focused on identifying therapeutic targets without grading long-term clinical trial validation data.
Feng et al., 2023 [373]	ASD biomarker pathways	Translational Literature Review	Probiotic administration models	Gut–brain axis communication, neuroimmune signaling	Outlined distinct behavioral and gastrointestinal improvements mediated by the downregulation of pro-inflammatory cytokine pathways.	Strictly narrative and mechanistic interpretation; lacks original primary human database pooling or meta-analytical verification.
Kotowska et al., 2024 [374]	Children and adolescents with ASD (12 RCTs; N = 630)|Systematic Review & Meta-analysis of RCTs	Various probiotic strains, doses, and durations	Core ASD symptoms, including social communication and restricted	repetitive behaviors	A small/borderline pooled beneficial effect on core ASD symptoms was identified, particularly in parallel-group trials; other pooled outcomes did not show significant between-group differences	Nine included studies were judged at high risk of bias; substantial heterogeneity in interventions and outcome measures; overall evidence was considered insufficient to support probiotics for core ASD symptoms with high certainty.
Zeng et al., 2024 [363]	Children with ASD (Pooled analysis of randomized controlled trials)	Meta-analysis of RCTs	Standardized probiotic regimens	Gastrointestinal distress scores, core autism symptoms	Reported modest beneficial effects on gastrointestinal and behavioral outcomes, with effect estimates varying across outcome domains	Constrained by variable compliance rates across original cohorts; highlights the immediate need for long-term follow-up tracking.
Soleimanpour et al., 2024 [362]	Individuals with ASD (8 studies; pooled N = 318; approximately 1.5–20 years)	Systematic Review & Meta-analysis	Heterogeneous probiotic strains, doses, and treatment durations	ASD behavioral symptoms and related clinical outcomes	Pooled analysis suggested improvement in behavioral symptom measures with probiotic supplementation compared with control conditions.	Small number of included studies; heterogeneous strains, doses, intervention durations, and behavioral scales; findings should be interpreted cautiously and do not establish strain-specific or broad clinical efficacy.
Barba-Vila et al., 2025 [367]	Children and adolescents with ASD	Systematic Literature Review	Variable probiotics and synbiotic matrices	Behavior regulation, gastrointestinal comfort, neuroinflammation	Concluded that the clinical evidence remains heterogeneous while discussing neuroinflammatory and microbiota–gut–brain mechanisms as potential pathways underlying probiotic effects.	Narrative and qualitative limits; strongly reinforces the urgent, clear imperative for large-scale, strictly standardized human RCTs.
Sun et al., 2025 [368]	Diverse pediatric neurodevelopmental cohorts	Narrative Literature Review	N/A (Review framework)	Neuroinflammation markers, adaptive behaviors	Highlighted promising therapeutic trends regarding behavioral stabilization, but global data remain inconclusive for routine clinical guidelines.	Qualitative assessment of an emerging translational field; definitive clinical dosing recommendations cannot yet be extracted.
Gao et al., 2025 [369]	Children with ASD and comorbid intellectual disability	Systematic Review	Standardized probiotic interventions	Core and secondary ASD symptom scales	Documented small absolute overall therapeutic effects on core social communication metrics, alongside high safety profiles.	Highly restricted by the limited number of primary multi-center trials available; highlights the immediate need for more rigorous protocols.
Huang et al., 2025 [365]	Individuals with ASD (8 randomized placebo-controlled trials; N = 465)	Systematic Review & Meta-analysis	Single- and multi-strain probiotic interventions	Overall ASD behavioral symptoms and subgroup outcomes	Probiotics were associated with a small improvement in total ASD symptoms (SMD −0.19, 95% CI −0.38 to −0.01); subgroup signals were observed in predominantly preschool populations and interventions lasting >3 months.	Certainty of evidence was low; small pooled effect size; heterogeneous populations and probiotic regimens; subgroup findings require cautious interpretation.
Tang et al., 2025 [366]	Children with ASD (8 randomized studies)	Systematic Review & Meta-analysis	Heterogeneous probiotic formulations, including single- and multi-strain interventions; variable treatment durations	ASD behavioral symptom severity; gastrointestinal symptoms	Probiotic interventions were associated with a small improvement in behavioral symptom severity (SMD = −0.251, 95% CI −0.466 to −0.037; *p* = 0.04). No significant pooled effect was observed for gastrointestinal symptoms (SMD = −0.048, 95% CI −0.399 to 0.303; *p* = 0.41).	Limited number of studies and generally small samples; heterogeneity in probiotic formulations, intervention durations, and assessment scales. Only three studies contributed data to the GI analysis. Further adequately powered RCTs are required.
Chan et al., 2026 [370]	Children and adolescents with ASD (14 clinical studies; N = 924; 10 RCTs)	Systematic Review	Single-strain and multi-strain probiotics, with heterogeneous formulations, doses, treatment durations, and some combination interventions	Core ASD domains, including social/communication functioning, restricted and repetitive behaviors, sensory/cognitive processing, and adaptive functioning	Findings across clinical studies were inconsistent, with some studies reporting improvements in selected ASD-related domains while others found no significant effects. Overall, the available evidence did not establish consistent efficacy of probiotic supplementation for core ASD symptoms.	Marked heterogeneity in probiotic strains, doses, intervention duration, study designs, and outcome measures; inclusion of randomized, non-randomized, and open-label studies; dietary and concurrent behavioral interventions were not consistently controlled. Narrative synthesis was used because quantitative pooling was not considered appropriate.

Abbreviations: ASD = Autism Spectrum Disorder; ADHD = Attention-Deficit/Hyperactivity Disorder; RCT = Randomized Controlled Trial; VPA = Valproic Acid; SCFAs = Short-Chain Fatty Acids; GOS = Galacto-Oligosaccharides; SHIME = Simulator of the Human Intestinal Microbial Ecosystem; VTA = Ventral Tegmental Area; SMD = Standardized Mean Difference; CI = Confidence Interval; GI = Gastrointestinal; N = number of participants; N/A = Not Applicable.

**Table 25:** Clinical Studies Evaluating Vitamin Supplementation in ASD.

Reference	Population (N)	Study Type	Dose & Duration	Primary Endpoints	Key Results	Study Limitations/Risk of Bias/Notes
Frye et al., 2013 [411]	Children with ASD (N = 37 completed)	Open-Label Clinical Intervention Study	Subcutaneous methyl-B12 75 µg/kg every 3 days plus oral folinic acid 400 µg twice daily for 3 months	Transmethylation/transsulfuration metabolites, glutathione redox status, adaptive/behavioral measures	Treatment was associated with improvements in selected methylation/transsulfuration biomarkers and behavioral measures.	Open-label design; no placebo group; combined methyl-B12/folinic-acid intervention prevents attribution of effects specifically to methyl-B12; small sample.
Hendren et al., 2016 [412]	Children with ASD (N = 57 randomized, 50 completed)	Randomized, Double-Blind, Placebo-Controlled Trial (RCT)	Subcutaneous *methyl-B12* injection (75 µg/kg) every 3 days for 8 weeks	Clinical Global Impressions-Improvement (CGI-I) scale, Vineland Adaptive Behavior Scales (VABS)	Methyl-B12 was associated with improvement on the clinician-rated CGI-I compared with placebo, whereas several secondary behavioral measures did not show significant between-group differences; treatment was generally well tolerated.	Moderate overall sample size executed within a single-center framework; relatively brief longitudinal follow-up to evaluate long-term maintenance.
Frye et al., 2016 [408]	Children with ASD and comorbid language impairment (N = 48)	Randomized, Double-Blind, Placebo-Controlled Trial (RCT)	High-dose oral folinic acid, 2 mg/kg/day (maximum 50 mg/day) for 12 weeks	Verbal communication assessments (CELF scales), folate receptor alpha autoantibody (FRα-Ab) status	The treatment effect on verbal communication was greater in FRα-Ab-positive participants.	Small-scale study population (N = 48); limited interventional window; primary selection criteria restricted exclusively to specific language-impaired phenotypes.
Kerley et al., 2017 [405]	42 children with ASD. (38 children completed the trial)	Double-blind, randomised, placebo-controlled trial	2000 IU vitamin D3 supplementation or placebo daily for 20 weeks.	ASD symptoms (ABC, SRS, DD-CGAS), serum 25(OH)D	Increase in 25(OH)D but no effect on the primary endpoint. Improvement in self-care on DD-CGAS (*p* = 0.02) and decreased inappropriate speech in the placebo group (*p* = 0.08).	Small sample; modest baseline deficiency; single dose regimen; limited power to detect subgroup effects.
Petruzzelli et al., 2020 [404]	Children with ASD (n = 54) vs. children with other neurological/psychiatric disorders (n = 36)	Cross-sectional case–control study	Serum 25(OH)D	Vitamin D status and ASD diagnosis	Mean 25(OH)D was lower in ASD; vitamin D deficiency was associated with ASD status	Cross-sectional observational design; non-neurotypical comparison group; no causal or therapeutic inference.

Abbreviations: ASD = Autism Spectrum Disorder; RCT = Randomized Controlled Trial; methyl-B12 = Methylcobalamin (Vitamin B12); CGI-I = Clinical Global Impressions–Improvement; VABS = Vineland Adaptive Behavior Scales; CELF = Clinical Evaluation of Language Fundamentals; FRα-Ab = Folate Receptor Alpha Autoantibodies; ABC = Aberrant Behavior Checklist; SRS = Social Responsiveness Scale; DD-CGAS = Developmental Disabilities–Children’s Global Assessment Scale; 25(OH)D = 25-Hydroxyvitamin D; IU = International Units; N = number of participants; n = number of participants in a subgroup; µg/kg = micrograms per kilogram; mg/kg/day = milligrams per kilogram per day; mg/day = milligrams per day.

**Table 26:** Secondary Research, Observational, and Mechanistic Evidence for Vitamins.

Reference	Scope/Dataset/Population	Study Type	Dose/Analysis Evaluated	Core Outcomes Evaluated	Key Synthesized Results	Study Limitations/Risk of Bias/Notes
Schmidt et al., 2012 [406]	Children aged 2–5 years in the CHARGE study: ASD n = 429, developmental delay n = 130, typically developing controls n = 278 (N = 837)	Population-based case–control study	Maternal periconceptional folic acid intake estimated from vitamins, supplements, and fortified cereals	ASD and developmental-delay diagnosis; maternal folic acid intake; one-carbon metabolism genotypes	Maternal folic acid intake ≥600 μg/day during the first month of pregnancy was associated with lower odds of ASD compared with <600 μg/day (adjusted OR 0.62, 95% CI 0.42–0.92); associations varied according to selected one-carbon metabolism genotypes	Retrospective assessment of maternal intake; observational case–control design; residual confounding and recall bias possible; association does not establish prevention or causality.
Surén et al., 2013 [415]	Norwegian Mother and Child Cohort Study (MoBa), N = 85,176 children	Population-based prospective cohort study	Maternal folic acid supplementation from 4 weeks before to 8 weeks after conception	Specialist-confirmed ASD diagnoses	Periconceptional folic acid use was associated with lower odds of autistic disorder (adjusted OR 0.61, 95% CI 0.41–0.90); no significant association was established for Asperger syndrome or PDD-NOS	Observational exposure; residual confounding possible; findings do not demonstrate a causal preventive effect.
Altun et al., 2018 [401]	ASD n = 60 + healthy controls n = 45 = N = 105	Case–Control Observational Study	Comprehensive serum vitamin profile analysis	Vitamin D, B6, and folate concentration levels	Reported lower serum concentrations of vitamins D, B6, and folate in the ASD group compared with controls.	Strictly correlational and cross-sectional design; non-interventional framework prevents the determination of direct causal therapeutic mechanisms.
Lee et al., 2021 (epub 2019) [402]	Stockholm Youth Cohort; maternal sample: 449 ASD cases/574 controls; neonatal sample: 1399 ASD cases/1607 controls; paired maternal–neonatal sample: 340 ASD cases/426 controls	Register-based cohort study with nested biomarker case–control analyses	Maternal and neonatal serum/blood-spot 25-hydroxyvitamin D [25(OH)D] concentrations	Maternal and neonatal 25(OH)D status; later ASD diagnosis; analyses stratified by co-occurring intellectual disability	Maternal 25(OH)D was not associated with ASD overall. Among Nordic-born mothers, maternal insufficiency was associated with higher ASD odds. Neonatal 25(OH)D <25 nmol/L was associated with higher ASD odds than ≥50 nmol/L (OR 1.33, 95% CI 1.02–1.75). Associations differed in analyses stratified by co-occurring ID.	Observational biomarker study; residual confounding cannot be excluded; subgroup estimates had lower precision; findings indicate associations and do not establish causality or benefit from vitamin D supplementation.
Principi et al., 2020 [403]	Pediatric neuroimmune networks	Comprehensive Literature Review	Vitamin *D* immunomodulatory action	Developmental vitamin D status, ASD risk, neurodevelopmental and immune mechanisms	Reviewed evidence linking developmental and maternal vitamin D deficiency with neurodevelopmental, immune, and biological mechanisms potentially relevant to ASD, while emphasizing that available evidence was insufficient to establish causality or definitive preventive/therapeutic effects.	N/A (Narrative Review); narrative synthesis format with a narrow immunomodulatory focus; lacks statistical pooling of primary outcomes.
Rossignol & Frye, 2021 [409]	Children with ASD and language deficits	Systematic Review & Meta-analysis	*Folinic acid*/Leucovorin administration	Communication performance, attention, stereotypy	Synthesized evidence suggesting potential improvements in communication and selected behavioral outcomes with folinic acid, particularly in FRα-Ab-positive subgroups; findings remain limited by small studies and heterogeneous designs	Marked by standardized dosage variability across historical cohorts; requires wider multi-center confirmation to define global guidelines.
Jennings et al., 2022 [414]	ASD; nutritional and metabolic literature involving amino acids, B vitamins, and choline	Narrative Review	B vitamins, amino acids, and choline; heterogeneous nutritional exposures/interventions	ASD risk, one-carbon metabolism, neurotransmitter synthesis, and core ASD-related outcomes	Reviewed potential relationships between B-vitamin status and ASD-related biological and clinical features, including the role of vitamin B6 as a cofactor in neurotransmitter metabolism; clinical supplementation evidence remained limited and inconsistent.	Broad narrative review encompassing multiple nutrients; heterogeneous evidence base; limited controlled evidence supporting B6 supplementation specifically.
Roufael et al., 2023 [407]	One-carbon pathways in neurodevelopment	Systematic Review	Folate-dependent metabolic cascades	Methylation efficiency, transsulfuration	Reviewed evidence of folate–methionine cycle abnormalities in ASD, including elevated homocysteine and reduced folate and vitamin B12 levels in some populations, and discussed potential nutritional and metabolic interventions.	Heterogeneous observational and interventional evidence; metabolic abnormalities may be influenced by dietary selectivity and nutritional status; limited ability to establish causality or treatment efficacy.
Jiang et al., 2024 [416]	Children with autism spectrum disorder	Systematic Review & Meta-analysis	Standardized Vitamin *D* formulations	Global ASD symptom clusters, irritability	Reported mixed pooled findings across ASD symptom domains; some analyses suggested improvement in selected outcomes, but results were inconsistent and sensitive to study characteristics	Constrained by varying compliance rates across pooled trial datasets; optimal therapeutic dosing windows remain undefined due to cross-study variations.
Zwierz et al., 2025 [413]	Methylation biomarkers in pediatrics	Comprehensive Literature Review	Methylcobalamin (Methyl-B12) protocols	Glutathione production, adaptive behavioral scores	Reviewed evidence linking vitamin B12 status, one-carbon metabolism, homocysteine, and redox regulation to ASD, while noting limited and heterogeneous interventional evidence.	N/A (Literature Synthesis); narrative overview of updated data; requires larger, standardized, multi-center randomized controlled trials to unify clinical dosing.
Avram et al., 2025 [417]	Prenatal to pediatric ASD populations; vitamin D, B12 and homocysteine datasets	Systematic Review & Meta-analysis	Biomarker exposure/status rather than standardized therapeutic intervention	Vitamin D, B12, homocysteine, ASD risk and metabolic profiles	Lower prenatal vitamin D status was associated with higher ASD risk, while relationships involving B12 and homocysteine were complex and exposure-dependent	Predominantly observational evidence; biomarker associations cannot establish treatment efficacy or causal benefit from supplementation.
Claussen & Pahr-Hosbach, 2026 [418]	Children with ASD; observational studies and placebo-controlled vitamin D trials	Critical Systematic Review	Heterogeneous vitamin D doses and treatment durations	Serum 25(OH)D status and ASD-related clinical outcomes	Observational associations were more consistent than treatment effects; placebo-controlled trials did not establish vitamin D as a generally effective ASD-targeted intervention.	Small and heterogeneous RCT evidence base; variable baseline vitamin D status, doses, duration, and outcome measures
Bandini et al., 2026 [419]	Maternal vitamin D status during pregnancy; 15 studies evaluating ASD and/or ADHD outcomes	Systematic Review and Dose–Response Meta-analysis	Maternal serum 25(OH)D exposure	Offspring ASD and ADHD risk	Higher maternal vitamin D status was associated with modestly lower ASD and ADHD risk; ASD risk decreased in dose–response analyses with increasing maternal 25(OH)D concentrations.	Observational exposure data; residual confounding cannot be excluded; findings do not demonstrate that vitamin D supplementation prevents ASD or ADHD.

Abbreviations: ASD = Autism Spectrum Disorder; ADHD = Attention-Deficit/Hyperactivity Disorder; RCT = Randomized Controlled Trial; CHARGE = Childhood Autism Risks from Genetics and Environment; MoBa = Norwegian Mother, Father and Child Cohort Study; PDD-NOS = Pervasive Developmental Disorder–Not Otherwise Specified; OR = Odds Ratio; CI = Confidence Interval; 25(OH)D = 25-Hydroxyvitamin D; ID = Intellectual Disability; FRα-Ab = Folate Receptor Alpha Autoantibodies; B6 = Vitamin B6; B12 = Vitamin B12; N = number of participants; n = number of participants in a subgroup; N/A = Not Applicable; μg/day = micrograms per day; nmol/L = nanomoles per liter.

**Table 27:** Clinical Studies Evaluating Magnesium (±Vitamin B6) in Neurodevelopmental Disorders.

Reference	Population (N)	Study Type	Dose & Duration	Primary Endpoints	Key Results	Study Limitations/Risk of Bias/Notes
Martineau et al., 1985 [447]	Children with autism (N = 60)	Four crossed-sequential double-blind therapeutic trials	Vitamin B6, magnesium, and combined B6–Mg protocols,	Behavioral measures; urinary homovanillic acid (HVA); evoked potentials (EPs)	Behavioral improvement was reported with combined B6–Mg and was accompanied by decreased urinary HVA and changes in EP amplitude/morphology; comparable changes were not observed with B6 or Mg alone	Historical study with a complex crossed-sequential design and limited reporting according to contemporary trial standards; findings were not subsequently confirmed by adequately powered modern RCTs.
Martineau et al., 1988 [448]	Children with autism previously identified as responsive/sensitive to B6–Mg (N = 11)	Open middle-term study	Combined vitamin B6–magnesium treatment for 8 weeks	Clinical, electrophysiological, and biochemical measures	Changes in clinical, electrophysiological, and biochemical measures were reported during treatment in this preselected responder subgroup	Very small sample; open design; participants were selected for previous sensitivity to B6–Mg, introducing substantial selection bias and severely limiting generalizability to unselected ASD populations.
Mousain-Bosc et al., 2006 [449]	Children with ADHD (N = 40)	Prospective uncontrolled clinical intervention	Magnesium 6 mg/kg/day + vitamin B6 0.6 mg/kg/day for ≥8 weeks	ADHD-related clinical symptoms and erythrocyte magnesium concentrations	Clinical symptoms improved during Mg–B6 supplementation and erythrocyte magnesium concentrations increased; symptoms reportedly worsened after treatment discontinuation.	Uncontrolled design; no randomized placebo group; small sample; combined Mg–B6 intervention prevents attribution of effects specifically to magnesium; regression to the mean and expectancy effects cannot be excluded.
Mousain-Bosc et al., 2006 [452]	Children with pervasive developmental disorder/autistic symptoms (N = 33)	Prospective uncontrolled clinical intervention	Magnesium 6 mg/kg/day + vitamin B6 0.6 mg/kg/day; ≥6-month follow-up	Clinical/behavioral symptoms and erythrocyte magnesium concentrations	Mg–B6 supplementation was associated with improvement in selected clinical and behavioral symptoms together with increased erythrocyte magnesium concentrations; symptoms reportedly worsened in some children following treatment discontinuation.	Uncontrolled, non-randomized design; small sample; combined Mg–B6 intervention prevents attribution of effects specifically to magnesium; reliance on clinical/behavioral assessments and absence of a placebo group substantially limit causal interpretation.
Khan et al., 2021 [453]	Children aged 2–12 years with ASD and hyperactivity; 70 enrolled, 50 analyzed per protocol (Mg + B6 n = 27; placebo n = 23)	Randomized, double-blind, placebo-controlled trial	Daily Mg + B6 for 3 months; age-dependent doses: Mg 50–200 mg/day + vitamin B6 25–100 mg/day	ADCL domains: general observation, cognition, emotion, social behavior, communication and sensory deficits	Greater overall improvement was reported in the Mg + B6 group than placebo, with significant effects reported particularly for emotion and cognition	Small sample; substantial difference between ITT enrollment and analyzed sample; combined Mg + B6 intervention prevents magnesium-specific attribution; single-center study
Adams et al., 2018 [193]	Children and adults with ASD (N = 67; treatment n = 37, non-treatment n = 30); additional neurotypical comparison group n = 50	Randomized controlled, single-blind 12-month trial	Comprehensive nutritional and dietary intervention including vitamin/mineral supplementation; 12 months.	Autism symptoms, adaptive functioning, nonverbal IQ, nutritional and metabolic measures	The comprehensive nutritional and dietary intervention was associated with improvements in several nutritional, metabolic, and neurodevelopmental outcomes compared with the control condition.	Not a magnesium-specific intervention; no placebo; participants were not blinded; multicomponent protocol prevents attribution of effects to magnesium or any individual nutrient

Abbreviations: ASD = Autism Spectrum Disorder; ADHD = Attention-Deficit/Hyperactivity Disorder; RCT = Randomized Controlled Trial; B6 = Vitamin B6; Mg = Magnesium; B6–Mg = Vitamin B6–Magnesium; HVA = Homovanillic Acid; EPs = Evoked Potentials; ADCL = Autism Developmental Checklist; ITT = Intention-to-Treat; IQ = Intelligence Quotient; N = number of participants; n = number of participants in a subgroup; mg/kg/day = milligrams per kilogram per day; mg/day = milligrams per day.

**Table 28:** Secondary Research, Observational, and Mechanistic Evidence for Magnesium.

Reference	Scope/Pooled Dataset/Population	Study Type	Dose/Analysis Evaluated	Core Outcomes Evaluated	Key Synthesized Results	Study Limitations/Risk of Bias/Notes
Nye & Brice, 2005 [450]	Pediatric ASD cohorts Three small randomized/crossover studies in ASD/PDD (total N = 33)	Cochrane Systematic Review	Combined *Vitamin B6* and Magnesium therapy	Global ASD symptoms, language development, safety indices	Concluded that baseline evidence remained insufficient to recommend routine clinical use due to low statistical power and methodological flaws across early trials.	Qualitative Cochrane design framework; heavily constrained by the systematic small sample sizes and high risk of bias in historical primary literature.
Murza et al., 2010 [451]	Children and adolescents with ASD	Brief literature review/research-status review	Variable Magnesium and Vitamin B6 interventions	Behavioral modification metrics, communication scales	Reconfirmed that available cumulative datasets remained inconclusive despite acknowledging selective, isolated clinical behavioral benefits.	Narrative synthesis constraints; high methodological heterogeneity in dosing guidelines and diagnostic scoring scales across the reviewed cohorts.
Mousain-Bosc et al., 2011 [454]	Neurodevelopmental populations (ASD/ADHD)	Mechanistic & Translational Review	N/A (Review framework)	Neurotransmission pathways, membrane stabilization, behavior	Discussed a possible relationship between altered magnesium status, NMDA-receptor-mediated excitability, neurotransmission, and behavioral abnormalities in neurodevelopmental disorders.	Narrative/mechanistic review; does not establish intracellular magnesium deficiency as a causal mechanism in ASD or ADHD and provides no quantitative estimate of supplementation efficacy.
Skalny et al., 2020 [446]	148 boys aged 4–9 years old including 44 children with ADHD, 40 pediatric patients with ASD, 32 patients with both ADHD and ASD, and 32 healthy neurotypical children.	Case-Control Biomarker Study	Serum, hair, and urinary elemental magnesium analysis	Systemic vs. tissue magnesium distribution patterns	Reported differences in magnesium-related biomarker profiles across children with ASD and/or ADHD, suggesting altered magnesium status or distribution in these populations	Cross-sectional observational design; peripheral magnesium biomarkers do not directly establish intracellular or central nervous system magnesium deficiency, causality, or therapeutic responsiveness to supplementation
Almohmadi et al., 2024 [455]	Pediatric neurodevelopmental cohorts	Narrative/Integrative Review	Magnesium profiles and synergistic nutritional factors	Neuroinflammation pathways, gastrointestinal motility, behavioral regulation	Outlined an integrative physiological perspective linking altered magnesium homeostasis to chronic neuroinflammation, GI dysfunction, and behavioral dysregulation.	N/A (Integrative Review); high qualitative variance across the compiled literature; lacks standardized statistical data pooling or formal risk-of-bias grading.
Kalejahi & Noorazar, 2025 [456]	Children with ADHD; studies of magnesium status and supplementation	Systematic Review	Heterogeneous magnesium biomarker assessments and supplementation protocols	Magnesium status; ADHD symptoms; response to magnesium supplementation	Reviewed evidence suggesting that altered magnesium status may occur in some children with ADHD and that supplementation has shown potential symptom benefits in selected studies; however, the evidence was heterogeneous and insufficient to establish magnesium as an effective stand-alone ADHD treatment.	Small and heterogeneous primary studies; variable magnesium biomarkers, formulations, doses, and co-supplementation; limited high-quality placebo-controlled evidence.
Angulo Sepúlveda et al., 2026 [457]	Magnesium supplementation in ASD and ADHD	Systematic Review	Heterogeneous magnesium interventions, including combined supplementation	Behavioral and neurodevelopmental outcomes in ASD and ADHD	Synthesized available clinical evidence concerning magnesium supplementation in ASD and ADHD; potential benefits were reported in some studies, but the evidence base remained heterogeneous and heavily dependent on small or methodologically limited studies and combined interventions	Limited number and quality of primary studies; heterogeneous populations, formulations and outcomes; frequent co-administration with vitamin B6 or other nutrients limits magnesium-specific conclusions.

Abbreviations: ASD = Autism Spectrum Disorder; ADHD = Attention-Deficit/Hyperactivity Disorder; PDD = Pervasive Developmental Disorder; NMDA = N-Methyl-D-Aspartate; GI = Gastrointestinal; N = number of participants; N/A = Not Applicable.

**Table 29:** Interventions for biological pathway.

Biological Pathway	Main Interventions
**Oxidative Stress Reduction**	NAC, Curcumin, Resveratrol, CoQ10, Vitamins, Omega-3
**Neuroinflammation Modulation**	Curcumin, Omega-3, Probiotics, Resveratrol, Saffron (mechanistic associations)
**Mitochondrial Dysfunction**	L-Carnitine, CoQ10, NAC, Resveratrol
**Glutamatergic/GABAergic Balance**	Magnesium, *NAC*, Saffron
**Serotonergic/Dopaminergic Regulation**	Saffron, Phosphatidylserine, *Bacopa*
**Gut–Brain Axis Modulation**	Probiotics, Omega-3, Curcumin
**Synaptic Plasticity/BDNF/NGF**	*Bacopa*, *Hericium erinaceus*, Omega-3, Resveratrol
**Methylation/One-Carbon Metabolism**	Folates, B12, B6

Abbreviations: NAC = N-Acetylcysteine; CoQ10 = Coenzyme Q10; GABA = Gamma-Aminobutyric Acid; BDNF = Brain-Derived Neurotrophic Factor; NGF = Nerve Growth Factor; B12 = Vitamin B12; B6 = Vitamin B6.

**Table 30:** Potential Biological/Clinical Phenotypes Relevant to Future Personalized Approaches.

Biological/Clinical Phenotype	Potentially Most Relevant Interventions
**Oxidative stress/mitochondrial dysfunction**	*NAC*, *CoQ10*, L-carnitine, Resveratrol
**Neuroinflammatory phenotype**	Curcumin, Omega-3, Probiotics
**Hyperactivity/irritability**	Saffron, *NAC*, Magnesium, Omega-3
**Cognitive impairment/executive dysfunction**	*Bacopa*, Phosphatidylserine, *Hericium*
**Sleep dysregulation/anxiety**	Saffron, Magnesium, Passiflora
**GI dysfunction/dysbiosis**	Probiotics, Omega-3
**Folate metabolism abnormalities**	Folinic acid, B12, B6

Proposed intervention–phenotype relationships are hypothesis-generating and do not represent validated predictive biomarkers or established treatment-selection criteria. Abbreviations: NAC = N-Acetylcysteine; CoQ10 = Coenzyme Q10; GI = Gastrointestinal; B12 = Vitamin B12; B6 = Vitamin B6.

## Data Availability

No new data were created or analyzed in this study. Data sharing is not applicable to this article.

## Abbreviations

The following abbreviations are used in this manuscript:

ID	Intellectual disability
ASD	Autism spectrum disorder
ADHD	Attention-deficit/hyperactivity disorder
